# Clinical Spectrum, Radiological Findings, and Outcomes of Severe Toxoplasmosis in Immunocompetent Hosts: A Systematic Review

**DOI:** 10.3390/pathogens12040543

**Published:** 2023-03-31

**Authors:** John Layton, Danai-Christina Theiopoulou, David Rutenberg, Amro Elshereye, Yumeng Zhang, John Sinnott, Kami Kim, Jose G. Montoya, Despina G. Contopoulos-Ioannidis

**Affiliations:** 1 Stanford Prevention Research Center, Stanford, CA 94304, USA; 2 School of Medicine, University of Crete, 71003 Heraklion, Greece; 3 Department of Medicine, Division of Infectious Disease and International Medicine, Morsani College of Medicine, University of South Florida, Tampa, FL 33602, USA; 4 Dr Jack S. Remington Laboratory for Specialty Diagnostics, Palo Alto Medical Foundation, Palo Alto, CA 94301, USA; 5 Department of Pediatrics, Division of Infectious Diseases, Stanford University School of Medicine, Stanford, CA 94305, USA; 6 Meta Research Innovation Center at Stanford, Stanford, CA 94305, USA

**Keywords:** severe toxoplasmosis, immunocompetent host, pulmonary toxoplasmosis, cardiac toxo-plasmosis, CNS toxoplasmosis, disseminated toxoplasmosis, fatal toxoplasmosis

## Abstract

Background: Accumulating evidence suggests that toxoplasmosis in immunocompetent hosts can be severe and life-threatening. Methods: We performed a systematic review of severe toxoplasmosis cases in immunocompetent patients to gain insight into the epidemiology, clinical characteristics, radiological findings, and outcomes of these cases. We classified severe toxoplasmosis as cases with the symptomatic involvement of target organs (the lungs, central nervous system (CNS), and heart), disseminated disease, prolonged disease (>3 months), or a fatal outcome. Our primary analysis focused on cases published from 1985–2022 to avoid confounding with cases in AIDS patients. Results: We identified 82 pertinent articles (1985–2022) with a total of 117 eligible cases; the top five countries for these cases were French Guiana (20%), France (15%), Colombia (9%), India (9%), and Brazil (7%). Overall, 44% (51/117) of cases had pulmonary involvement, 39% (46/117) CNS, 31% (36/117) cardiac, 24% (28/117) disseminated disease, 2% (2/117) had prolonged disease, and 8% (9/117) of patients died. More than one organ was involved in 26% (31/117) of cases. Eighty-four percent (98/117) of cases occurred in the context of a recent acute primary *Toxoplasma* infection; for the remaining, the exact timing of infection was unclear. Genotyping data were very sparse. Among those reporting genotyping data, 96% (22/23) were caused by atypical non-type II strains; one case was caused by a type-II strain. Only half of the cases reported risk factors. The most common risk factors were eating raw/undercooked meat or eating game meat (47% (28/60)), drinking untreated water (37% (22/60)), or living in a toxoplasmosis high-prevalence area (38% (23/60)). For the 51 pulmonary cases, the main clinical presentation was pneumonia or pleural effusions in 94% (48/51) and respiratory failure in 47% (24/51). For the 46 CNS cases, the main clinical presentation was encephalitis in 54% (25/46), meningitis in 13% (6/46), focal neurologic findings in 24% (11/46), cranial nerve palsies in 17% (8/46), Guillain–Barre syndrome or Miller Fisher syndrome in 7% (3/46), and Brown–Sequard syndrome in 2% (1/46) of cases; more than one clinical manifestation could also be present. Among the 41 CNS cases reporting the CNS imaging findings, 68% (28/41) had focal supratentorial lesions and 7% (3/41) had focal infratentorial lesions. Brain abscess-like/mass-like lesions were seen in 51% (21/41) of cases. For the 36 cardiac cases, the main clinical presentation was myocarditis in 75% (27/36), pericarditis in 50% (18/36), heart failure and/or cardiogenic shock in 19% (7/36), and cardiac arrhythmias in 22% (8/36); more than one manifestation could also be present. Illness was critical in 49% (44/90) of cases intensive care unit care was needed in 54% (29/54) of cases among those reporting this information, and 9 patients died. Conclusion: The diagnosis of severe toxoplasmosis in immunocompetent hosts can be challenging. Toxoplasmosis should be considered in the differential diagnosis of immunocompetent patients presenting with severe illness of unclear etiology with pulmonary, cardiac, CNS, or multiorgan involvement/failure, or prolonged febrile illness, even in the absence of common exposure risk factors or common manifestations of toxoplasmosis (e.g., fever, mononucleosis-like illness, lymphadenopathy, and chorioretinitis). Fatal outcomes can also rarely occur in immunocompetent patients. Prompt initiation of anti-*Toxoplasma* treatment can be lifesaving.

## 1. Background

Toxoplasmosis is a protozoal disease with worldwide distribution caused by the parasite *Toxoplasma gondii* (*T. gondii*) [1,2,3]. *T. gondii* infections cause significant morbidity and mortality worldwide with a wide spectrum of clinical manifestations both in immunocompromised and immunocompetent hosts. Severe fulminant and even life-threatening toxoplasmosis has been widely reported in congenitally infected offspring, immunocompromised patients with profound immune deficiencies, AIDS patients, transplant patients [4,5,6], and patients receiving chemotherapy, immunosuppressive medications, or novel biologic immunomodulatory agents [7,8]. In immunocompromised patients, severe toxoplasmosis is usually the result of the reactivation of latent infection in the host or the donor transplant organ. Acute primary infections acquired via the oral route can also occur in immunocompromised patients. In immunocompetent hosts, acute primary toxoplasmosis has been primarily viewed either as asymptomatic or minimally symptomatic with fever and mononucleosis-like symptoms, with/or without lymphadenopathy. However, acute primary infections in pregnant women can have devastating sequelae and can lead to congenital toxoplasmosis with severe neurologic or ocular manifestations and even death [9].

An accumulating body of evidence suggests that toxoplasmosis in immunocompetent hosts can cause severe and life-threatening infections. Acute *Toxoplasma* infections in immunocompetent hosts in certain tropical areas, with the atypical more virulent *T. gondii* strains and/or with a high parasite load, have been reported to cause severe disease, including disseminated disease and death [10,11,12]. Most cases of disseminated toxoplasmosis in immunocompetent hosts were originally reported from French Guyana and included pneumonia, myocarditis, encephalitis, hepatitis and myositis, and some of those also had fatal outcomes [10,11,12]. Unusual clinical presentations in immunocompetent hosts have also been reported after eating raw meat or wild game meat in diverse country settings [13,14]. High attack rates and severe clinical manifestations have also been reported in the setting of community outbreaks from the ingestion of *T. gondii*-contaminated municipal water [15].

We performed a systematic review of severe toxoplasmosis cases reported in the literature in immunocompetent hosts to gain insight into the epidemiology, clinical characteristics, radiological manifestations, and outcomes of these cases. Prior reviews had included a limited number of cases, were published more than 1–3 decades ago, and had focused only on specific organ systems or on cases from the Amazonian region only. [16,17,18,19]. We focused on severe toxoplasmosis cases in immunocompetent hosts with the symptomatic involvement of target organs (the lungs, central nervous system (CNS), and heart), disseminated disease, prolonged illness >3 months, or fatal outcomes. 

We wanted to increase the awareness among clinicians that in immunocompetent patients, toxoplasmosis should be considered in the differential diagnosis when patients present with severe pneumonia, acute respiratory failure, myocarditis, pericarditis, heart failure, arrhythmias, encephalitis, focal neurologic findings, rapidly progressing fulminant disease with multiorgan failure, and/or prolonged febrile illnesses of unknown origin, even in the absence of common exposure risk factors or common manifestations of toxoplasmosis (e.g., fever, mononucleosis-like illness, lymphadenopathy, and chorioretinitis). It is important to recognize that fatal outcomes can occur in immunocompetent hosts. Early diagnosis and prompt initiation of anti-*Toxoplasma* treatment can be life-saving.

## 2. Methods

**Figure 1:**
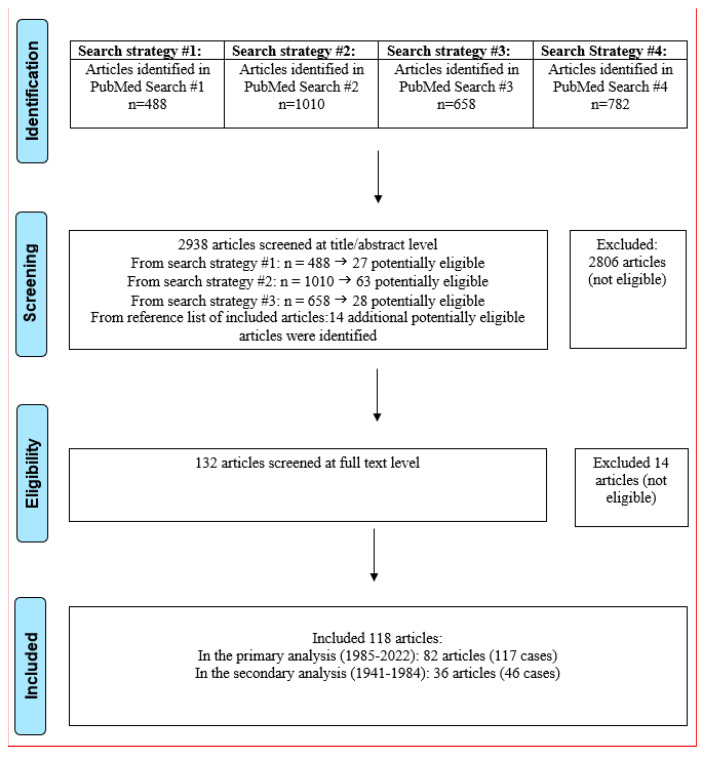
Search strategy 1: (toxoplasmosis OR toxoplasma gondii OR t.gondii) AND (severe OR fatal OR lethal OR disseminated OR fulminant OR multisystem OR muti-organ OR shock) AND (immunocompetent OR non immunocompromised OR not immunocompromised OR healthy). Filters: Human. Search Date: 22 August 2022. Search strategy 2: (toxoplasmosis OR toxoplasma gondii OR t.gondii OR toxoplasm*) AND (pulmonary OR lung OR myocarditis OR myopericarditis OR encephalitis OR brain OR cerebral OR central nervous system OR intracrania* OR intra-cranial OR seizures OR mental status changes OR myelopathy OR spinal cord OR acute disseminated encephalomyelitis OR ADEM OR hemophagocytic OR hepatitis OR nephritis OR nephrotic OR myositis OR pyomyositis OR complications OR atypical OR unusual) AND (immunocompetent OR non immunocompromised OR not immunocompromised OR healthy OR without immunosupression) Filters: Human. Search Date: 22 August 2022. Search strategy 3: (toxoplasmosis OR toxoplasma gondii OR t.gondii OR toxoplasm*) AND (pulmonary OR lung OR myocarditis OR myopericarditis OR encephalitis OR brain OR cerebral OR central nervous system OR intracrania* OR intra-cranial OR seizures OR mental status changes OR myelopathy OR spinal cord OR acute disseminated encephalomyelitis OR ADEM OR hemophagocytic OR hepatitis OR nephritis OR nephrotic OR myositis OR pyomyositis OR complications OR atypical OR unusual) AND (child (ti) OR adult (ti) OR woman (ti) OR patient(ti)) Filters: Human. Search Date: 22 August 2022. Search strategy 4: ((toxoplasmosis OR toxoplasma gondii OR t.gondii OR toxoplasma*) AND (pulmon* (ti) OR chest (ti) OR cardiac(ti) OR cardio*(ti) OR intensive care(ti) OR intubat*(ti) OR respiratory failure(ti) OR respiratory support(ti) OR neurol* (ti) OR neur* (ti) OR liver(ti) OR renal(ti) OR kidney(ti))) NOT search strategy #1, OR #2 OR #3.

We performed a systematic review of articles published in PubMed using three different search strategies (Appendix A). The reference list of eligible papers and pivotal prior review papers were also screened. The last search was performed on 22 August 2022. Details about our screening process are shown in the PRISMA flow chart (Figure 1). Article screening for eligibility at the title/abstract level was performed by one investigator (DCI); potentially eligible papers were retrieved in full text and further screened for eligibility for inclusion by 3 independent investigators (DCI, JL, and DT). Data extraction was performed by JL/DCI and DT/DCI. Discrepancies were solved by consensus (DCI/JGM/JL/DT). We followed the PRISMA guidelines for reporting of systematic reviews (Appendix A–PRISMA checklist). Individual study-level data are available from the authors upon request.

### 2.1. Inclusion Criteria

Eligible for inclusion were cases that fulfilled all the following criteria: (a) Severe disease, defined as cases with symptomatic involvement of the three target organs, namely the lungs, CNS, or heart; disseminated disease; prolonged illness of >3 months duration, or fatal outcome; (b) proper ascertainment of the diagnosis of toxoplasmosis, with toxoplasmosis being the most likely etiology of the reported severe clinical manifestations; and (c) an immunocompetent host. The diagnosis of toxoplasmosis was considered properly ascertained if supported by serologic, molecular, histopathologic or direct examination of body fluids methods, and/or mice sub-inoculation results. The clinical response to anti-*Toxoplasma* therapy was also used to support the diagnosis. Cases were considered pertaining to immunocompetent hosts if the patient was HIV negative, without underlying immunodeficiency (e.g., malignancy, chronic immunosuppressive medications, etc.) For the few cases not reporting HIV test results, information from long-term follow-up was also used to ascertain the immunocompetent status of the host (e.g., no findings or signs/symptoms of underlying immunodeficiency during long-term follow-up).

Articles of all study designs were considered eligible. When more than one publication from the same investigators was identified—with overlapping patient cases—we kept only the latest article with the largest number of cases reported. For articles published in languages other than English, French, or Spanish, we used Google translation to extract pertinent information.

In our primary analysis, we focused only on cases published between 1985 and 2022 to minimize confounding from cases in AIDS (acquired immunodeficiency syndrome) patients. Early AIDS cases were sporadically reported several years before 1980; the Center for Disease Control released the first definition for AIDS in 1982, the human immunodeficiency virus (HIV) genome was first isolated and described in 1983 [20,21], and the Food and Drug Administration (FDA) approved the first commercial HIV-ELISA test in 1985 [22].

In a secondary analysis, we separately reported the summary findings from the articles published before 1985 to be comprehensive. From these papers, we extracted only limited information (e.g., the title, year, country, and main clinical manifestations of severe toxoplasmosis), acknowledging that some early AIDS cases unavoidably could have been included in those cases.

### 2.2. Exclusion Criteria

We excluded cases describing severe complications of ocular toxoplasmosis or congenital toxoplasmosis, as these have been extensively reviewed previously [23,24,25,26,27]. Cases focusing only on well-known manifestations of toxoplasmosis (e.g., fever, lymphadenopathy, hepatitis, and myositis) were also excluded, unless these cases also had pulmonary, CNS, cardiac involvement, disseminated disease, prolonged illness duration (>3 months), or fatal outcomes.

### 2.3. Data Extraction

From each eligible case, we extracted information on the following: Country of authors, country where the *Toxoplasma* infection was most likely acquired, total number of cases reported per article (each eligible case was recorded separately), patient’s age, duration of symptoms (from onset of symptoms to admission), method for ascertainment of the toxoplasmosis diagnosis, type of toxoplasmosis infection (e.g., acute primary *Toxoplasma* infection or unclear timing of infection-based on provided information), type of implicated *T. gondii* strain (type II vs. non-type II atypical strain), reported risk factors, methods for ascertainment of immunocompetent status, category of severe toxoplasmosis manifestations (pulmonary, CNS, cardiac, disseminated disease, fatal disease, or prolonged illness), critical illness status and/or need for care in intensive care unit (ICU), presentation of clinical signs and symptoms, clinical manifestations and laboratory abnormalities throughout the clinical course, imaging findings from lungs, the CNS, or heart, type of anti-*Toxoplasma* treatment, patient’s outcome (improvement/cure or death), and time interval to the resolution of symptoms.

### 2.4. Analyses

We used descriptive statistics (*n*, %) to describe the epidemiologic, clinical, laboratory, and imaging findings of severe toxoplasmosis in immunocompetent hosts. We also created compilation lists for the presenting signs and symptoms, main clinical manifestations, and imaging findings (pulmonary, CNS, and cardiac).

## 3. Results

### 3.1. Primary Analysis (1985–2022)

**Figure 2:**
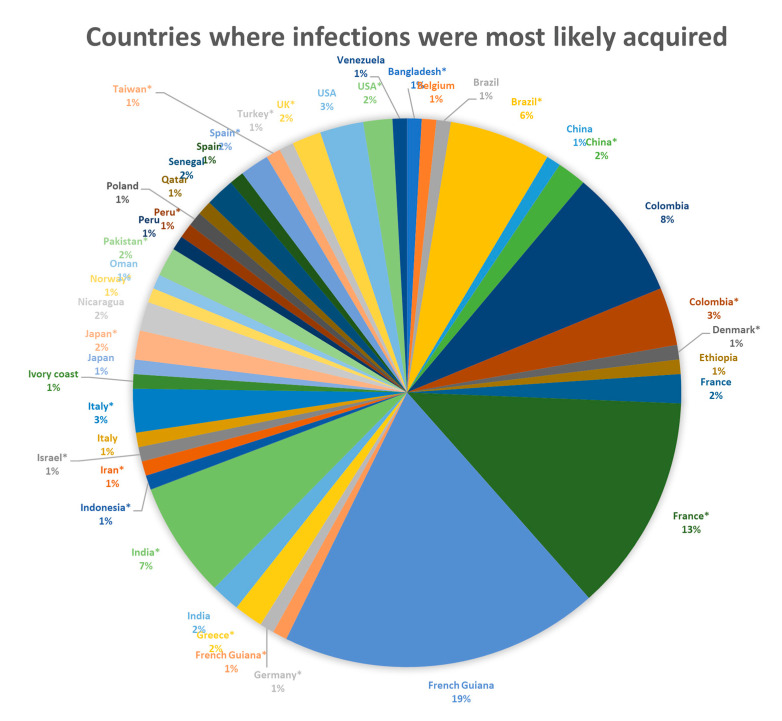
Countries where infections likely occurred (when information was missing, we imputed the country of authors, marking the country with an *).

After screening 2938 articles, we identified 82 eligible articles published between 1985 and 2022 (Appendix B), with a total of 117 eligible cases of severe toxoplasmosis in immunocompetent hosts (Figure 1). Cases were reported from 33 different countries (Figure 2). The top 5 countries where infections were most likely acquired included French Guiana (20%), France (15%), Colombia (9%), India (9%), and Brazil (7%) (Table 1, Appendix C-Table A1 and Table A2). We also identified five cases from North America (four with myocarditis [28,29,30,31] and one with encephalitis [32]); in two of those cases, the infections were acquired in the US [29,32]. Eighty-four percent of cases pertained to adults and 16% to children (Table 1). Moreover, 44% (51/117) of cases had pulmonary involvement, 39% (46/117) CNS, 31% (36/117) cardiac, 24% (28/117) disseminated disease (23 cases with disseminated disease also had pulmonary involvement, 14 cardiac, 9 pulmonary and cardiac, and 5 CNS involvement), and 2% (2/117) of cases had a prolonged duration of >3 months. Moreover, 26% (31/117) of cases had more than one target organ system involved.

### 3.2. Duration of Symptoms

The onset of symptoms (among the 83/117 cases reporting this information) was acute, <3 weeks prior to admission, in 75% (62/83) of cases; (acute/or subacute onset of symptoms < 3 months was reported in 93% (77/83) of cases). Respectively, acute onset of symptoms < 3 weeks represented 81% (25/31) of pulmonary cases, 66% (21/32) of CNS cases, 87% (20/23) of cardiac cases, and 85% (11/13) of disseminated cases; (acute/or subacute onset of symptoms < 3 months was reported in 97% (30/31) of pulmonary cases, 91% (29/32) of CNS cases, 100% (23/23) of cardiac cases, and 100% (13/13) of disseminated cases) (Table 1).

### 3.3. Diagnostic Methods

Serology was used for the diagnosis of toxoplasmosis in 95% (111/117) of cases, molecular methods in 19% (22/117), and histopathology (from tissue biopsies or autopsy) in 21% (24/117) of cases. The response to anti-*Toxoplasma* therapy also supported the diagnosis of toxoplasmosis in 80% (88/110) of cases, while for the remaining, this was unclear (not reported). More than one diagnostic method was used for 84% (98/117) of cases (Table 1).

### 3.4. Type of Infection

For 84% (98/117) of cases, severe toxoplasmosis occurred in the context of a recent acute primary *Toxoplasma* infection; for the remaining, the exact timing of infection was unclear (Table 1).

Genotyping data were sparse. Among those reporting genotyping data, 96% (22/23) were caused by atypical non-type II strains and one case was caused by a type-II strain (Table 1 and Appendix C-Table A3). This type II strain-associated infection was a case of spinal toxoplasmosis in a 31-year-old patient from France who presented with Brown–Sequard syndrome [33].

### 3.5. Risk Factors

Only half (60/117) of the cases reported risk factors for toxoplasmosis. The most common risk factors were eating raw or undercooked meat or game meat (47% (28/60)), drinking untreated water (37% (22/60)), or living in a high-prevalence toxoplasmosis region (38% (23/60)). In 32% (19/60) of cases, multiple risk factors were reported (Table 1). Examples of cases associated with the consumption of infected game meat included cases after eating venison [28], wild boar meat [33], maipouri meat from French Guiana [34], or after travel across the Greenland glacier during which undercooked meat was consumed [3]. Examples of cases associated with the drinking of untreated water included cases in hunters/forest policemen (a case from China [35], a case from the US after recent camping in Tennessee [29], and six cases from Colombia [36]), and an outbreak of cases from drinking contaminated community water (e.g., six cases in a community outbreak along the Maroni river [10]). Examples of cases in high-prevalence regions included 23 severe cases reported from French Guiana [10,11,12,34,37,38,39,40]. Several severe cases were also reported in military personnel, including (a) a case of acute myocarditis in a 20-year-old military fireman in France [41]; (b) a case of pneumonia in a 37-year-old in a military operation in the Peruvian Amazon [42]; (c) a military outbreak with six cases in military personnel deployed in rural areas in Colombia [36]; (d) a case of pneumonia with respiratory failure and disseminated disease in a 35-year old in a military operation in the forests of French Guyana (likely from drinking chemically disinfected river water) [38]; (e) a case of diffuse encephalitis with fatal outcome in a 35-year-old in a military operation in the Amazonian forest (in the context of an outbreak in additional military personnel) [39]; and (f) a case of pneumonia in a 32-year-old missionary in the jungle of Venezuela [43]. Some of the severe cases also occurred in the context of family outbreaks, with other family members reporting less severe disease than the index cases (e.g., cases in Venezuela [43] or the US [44]).

### 3.6. Ascertainment of Immunocompetent Status

Almost all cases published after 1985 were HIV-negative patients. There were 16 cases (14%) that did not report HIV test results; however, in those cases, underlying immunodeficiency was excluded based on information from a long-term follow-up. All ambiguous cases were excluded.

### 3.7. Toxoplasmic Manifestations

The main manifestations of the 51 pulmonary cases were pneumonia and/or pleural effusions in 94% (48/51) and respiratory failure in 47% (24/51) of cases. (Table 2) The main manifestations of the 46 CNS cases were encephalitis in 54% (25/46), meningitis in 13% (6/46), focal neurologic findings in 24% (11/46), cranial nerve palsies in 17% (8/46), Guillain–Barre/or Miller–Fisher syndrome in 7% (3/46), and spinal lesion/Brown–Sequard syndrome in 2% (1/46) (Table 2). The main manifestations of the 36 cardiac cases were myocarditis in 75% (27/36), pericarditis in 50% (18/36), heart failure or cardiogenic shock in 19% (7/36), and arrhythmias in 22% (8/36); several cases had more than one manifestation (Table 2).

Illness was critical in 49% (44/90) and ICU care was needed in 54% (29/54) of cases among those reporting. Nine patients died (8%).

Classic manifestations of toxoplasmosis (e.g., eye findings, lymphadenopathy, fever, and malaise) were not always reported. Ocular findings were reported only in 34% (18/53), lymphadenopathy in 74% (46/62), fever in 86% (87/101), and malaise in 68% (50/74) of cases; for the remaining cases (64, 55, 16, and 43 cases, respectively), this information was not reported. The presence of ocular findings and the lymphadenopathy provided the clues that led to the diagnosis of toxoplasmosis in 34% and 74% of cases, respectively.

### 3.8. Clinical Findings

Reported clinical findings and signs and symptoms of severe toxoplasmosis in immunocompetent hosts included any combination of the following general, respiratory, CNS, cardiac, and other findings: (a) Fever, chills, flu-like symptoms, anorexia, malaise, night sweats, and weight loss; (b) chest pain, cough, dyspnea, hypoxia, pneumonia, pleural effusions, acute respiratory failure, and the need for ICU care and/or mechanical ventilation; (c) confusion, agitation, mental status changes, memory loss, cognitive decline, rapid neurologic deterioration, emotional lability, personality changes, encephalitis, acute disseminated encephalomyelitis (ADEM), anti N-methyl-D-aspartate receptor encephalitis (NMDA), findings of brain-abscess/mass-like lesions, focal neurologic findings, spastic hemiparesis/quadriparesis, pyramidal symptoms, meningitis, meningoencephalitis, rhombencephalitis, cerebellar ataxia, headaches, seizures, central and peripheral neuropathies/nerve palsies, nystagmus, dysarthria, Guillain–Barre syndrome, Miller–Fisher syndrome (ataxia, ophthalmoplegia, areflexia), hydrocephalus, ventriculitis, and findings of spinal cord lesions (Brown–Sequard syndrome); (d) myocarditis, pericarditis, cardiac tamponade, myopericarditis, acute chest pain mimicking acute myocardial infarction, acute heart failure, cardiogenic shock, tachyarrhythmias, atrioventricular (AV) block, and AV dissociation; or (e) other findings, e.g., lymphadenopathy, myalgias, muscle weakness, myositis, arthralgias, abdominal pain, nausea, vomiting, diarrhea, dysphagia, ocular findings with blurry vision, decreased visual acuity, photophobia, chorioretinitis, papillitis, conjunctival injection, fever of unknown origin (FUO), hemophagocytic syndrome (HLH), [45,46] hepatitis, idiopathic thrombocytopenic purpura (ITP), nephrotic syndrome, nephritic syndrome, or skin rashes.

### 3.9. Laboratory Findings

Laboratory abnormalities reported in severe toxoplasmosis cases in immunocompetent hosts included, among others, leukopenia [30,35,38], lymphopenia [47,48,49], lymphocytosis [44,50], eosinophilia [51] (mild or severe), thrombocytopenia [35,52], thrombocytosis, anemia [35,53], transaminitis, elevated inflammatory markers, hyperferritinemia [35], elevated LDH [3,54], hyponatremia [34,47], elevated lipase [34], elevated cardiac biomarkers or elevated creatine-phosphokinase (CPK). A transiently low CD4 count [3,17,55] and CD4/CD8 ratio has been reported in the acute phase of severe acute toxoplasmosis in some non-HIV immunocompetent patients, with normalization after anti-*Toxoplasma* treatment [38,56].

### 3.10. Imaging Findings

#### 3.10.1. Lung Imaging

The most frequently reported lung imaging findings (among the 38/51 pulmonary cases reporting these findings) included (a) ground glass opacities or bilateral diffuse interstitial or alveolar infiltrates (82% (31/38)), (b) pleural effusions (47% (18/38)), (c) focal consolidations (16% (6/38)), or (d) nodular opacities (13% (5/38)). Mediastinal lymphadenopathy was reported in 11% (4/38) of cases (Table 3, Table 4, and Appendix C-Table A4).

#### 3.10.2. CNS Imaging

The CNS imaging findings (among the 41/46 CNS cases reporting these findings) showed focal supratentorial lesions in 68% (28/41) and focal infratentorial lesions in 7% (3/41) of cases; 2 cases had spinal lesions (5%) (Table 3, Table 4, and Appendix C-Table A5). Lesions had brain abscess/mass-like appearance in 51% (21/41) of cases, diffuse encephalitis appearance in 17% (7/41), meningitis in 10% (4/41), and meningoencephalitis in 5% (2/41).

#### 3.10.3. Cardiac Imaging

The cardiac imaging findings on cardiac ECHO, cardiac MRI, and/or EKG (among the 34/36 cardiac cases reporting these findings) showed myocarditis in 68% (28/34), pericarditis in 47% (16/34), myopericarditis in 34% (11/34), low cardiac function in 45% (13/34), or cardiac arrhythmia in 29% (10/34) of cases (Table 3, Table 4, and Appendix C-Table A6).

### 3.11. Anti-Toxoplasma Therapy

The types of anti-*Toxoplasma* treatments and the duration of treatment varied significantly across studies. The majority of cases were treated with pyrimethamine + sulfadiazine (55% (64/117)) or trimethoprim/sulfamethoxazole (14% (16/117)); however, non-standard anti-*Toxoplasma* therapies or monotherapies (e.g., spiramycin, azithromycin, erythromycin, or clindamycin) were also given in some cases (Appendix C-Table A7).

### 3.12. Outcomes

In total, 80% (88/110) of cases of severe toxoplasmosis in immunocompetent patients in whom the diagnosis was promptly considered and treatment was promptly initiated had favorable outcomes, with the improvement or resolution of symptoms and radiographic and laboratory abnormalities at the time of last follow-up (for 7/117 cases the outcome was not reported). In 12% (13/110) of cases, symptoms had improved or resolved without anti-*Toxoplasma* treatment or before anti-*Toxoplasma* treatment was initiated. Fatal outcomes were reported for 8% (9/110) of cases [10,11,35,39,54,57,59,60,61]; among those were four cases with disseminated disease and multiorgan failure, four had encephalitis, and one had a brain mass-like lesion. Eight fatal cases occurred in adults and one in a child [60] (Table 2).

The cases that improved or resolved without anti-*Toxoplasma* treatment or before anti-*Toxoplasma* treatment support the hypothesis for an immunologic mechanism for these manifestations (Table 2). These 13 cases included (a) a case of a 28-year-old from Indonesia with myopericarditis who initially improved with anti-inflammatory medications but subsequently relapsed [47]; anti-*Toxoplasma* therapy eventually led to complete resolution; (b) a case of a 59-year-old from the US with a history of eating game meat, with acute myocarditis and cardiogenic shock and multiorgan involvement that resolved without anti-*Toxoplasma* therapy (the diagnosis was made late, after the development of eye findings) [28]; (c,d) two cases from Colombia, [62] one of a 44-year-old with disseminated multiorgan disease, pneumonia respiratory failure, and myopericarditis and one of a 67-year old with pneumonia and acute respiratory failure (in both cases, the diagnosis was made late, after the development of eye findings); (e,f,g) three cases from France with acute myopericarditis that resolved with anti-inflammatory treatment only, without subsequent relapse [63,64,65,66]; (h) a case of a 76-year-old from Spain with ADEM during an acute *Toxoplasma* infection that resolved with steroids only [67]; (i) a pediatric case from Greece with myocarditis and polymyositis that resolved with steroids only [68]; (j) a case of a 30-year-old from Spain with acute CNS toxoplasmosis (Miller–Fisher syndrome) [69]; (k) a case of a 61-year-old from Spain with CNS toxoplasmosis and seizures [70]; (l) a case of pneumonia in a missionary in the Venezuela jungle—presumably from an Amazonian *T.gondii* strain—that eventually self-resolved after a prolonged febrile illness for 7 months (prior to anti-*Toxoplasma* treatment) [43]; and (m) a case of a 48-year-old from the US with myocarditis, polymyositis, and disseminated disease [31] (Table 2).

The nine fatal cases included (a) a case of acute CNS toxoplasmosis in a 14-year-old boy from India with brain mass-like lesions in the brainstem who died despite anti-*Toxoplasma* treatment for 2 weeks [60]; (b) a case of a 41-year-old male from India with acute CNS toxoplasmosis with multiple focal hypointense cerebral lesions, in whom the diagnosis of toxoplasmosis was made late and the patient died [57]; (c) a case of a 44-yearold male from Colombia, with multiorgan failure, pneumonia, respiratory failure, and CNS toxoplasmosis [54]; (d) a case of a 41-year old male from China with disseminated toxoplasmosis and multiorgan failure [35]; (e) a case of a 56-yearold from French Guiana with pneumonia and altered levels of consciousness [10]; (f) a case of an 18-year-old from French Guiana with disseminated disease, pneumonia, acute respiratory failure, and myopericarditis [11]; (g) a case of a 23-year-old male from Oman, with acute CNS toxoplasmosis, who presented with encephalitis and posterior fossa mass, who died after the neurosurgical procedure that established the diagnosis of toxoplasmosis [59]; (h) a case of 35-year-old military personnel on a military operation in Amazonian forest who died from diffuse encephalitis (this case occurred in the context of a military outbreak in additional military personnel from the same operation) [39]; and (i) a case of a 69-year-old from Croatia with rapidly progressing dementia due to multiple brain lesions in the basal ganglia. [61] In all the fatal cases, the duration of symptoms was <2 weeks prior to admission except for the last case by Habek et al. [61] who had a 2-month history of cognitive decline prior to admission with hyperintense brain lesions in both thalami and putamina who died from Gram-negative sepsis; the diagnosis of toxoplasmosis in this patient was made postmortem at the autopsy [61].

### 3.13. Time to Resolution

Data on the time to resolution of symptoms were very sparse. Among those reporting this information, symptoms were resolved in <4 weeks in 64% (23/36) of cases (Table 2).

A compilation list of clinical vignettes for the 117 included cases is shown in Appendix C-Table A8.

### 3.14. Secondary Analysis (1941–1984)

An additional 36 articles with 46 cases of severe toxoplasmosis were identified and published between 1941 and 1984. Of these cases, 17% (8/46) had pulmonary involvement, 26% (12/46) had CNS involvement, 35% (16/46) had cardiac involvement, and 13% (6/46) had disseminated disease (Appendix C-Table A9 and Table A10).

## 4. Discussion

This is the largest comprehensive systematic review of all severe toxoplasmosis cases with pulmonary, cardiac, CNS, disseminated disease, or fatal outcomes in immunocompetent patients to date. We identified 117 cases of severe toxoplasmosis in immunocompetent hosts published between 1985 and 2022 and reported from 33 countries. Forty-four percent of cases had pulmonary involvement, and half of those developed respiratory failure. Thirty-nine percent of cases had CNS involvement, with ~70% of those having focal supratentorial lesions and ~50% brain-abscess/mass-like lesions. Furthermore, 31% of cases had cardiac involvement with ~75% of those having myocarditis, 50% pericarditis, ~20% heart failure or cardiogenic shock, and ~20% cardiac arrhythmias. More than one target organ system was involved in approximately one-third of cases; disseminated disease was reported in one-quarter of cases. Critical illness and the need for ICU admission were reported for almost half of the cases reporting this information. Nine patients died.

Approximately 75% of cases had an acute onset of symptoms <3 weeks prior to admission, and for >90% of cases, the onset of symptoms was <3 months from admission. Classic exposure risk factors or classic clinical manifestations of toxoplasmosis (e.g., fever, malaise, lymphadenopathy, and chorioretinitis) were not always present. Almost all cases occurred in the context of a recently acquired acute primary toxoplasmosis infection. Genotyping data were very sparse, and the majority of the genotyped cases were from non-type-II strains. Nevertheless, a severe case from a type II strain was also reported. Only half of the cases reported risk factors for toxoplasmosis. Outbreaks among military personnel and hunters and family outbreaks were also reported. Not all members in these outbreaks experienced the same disease severity as the index cases. The reporting of these cases from so many diverse countries supports the hypothesis that severe toxoplasmosis in immunocompetent hosts is more widely encountered than previously thought and not limited to the Amazonian region.

Most immunocompetent patients (~80%) with acute severe toxoplasmosis had an excellent response to anti-*Toxoplasma* treatment if the diagnosis was promptly considered and anti-*Toxoplasma* therapy was initiated early. Few cases (~12%) improved or resolved without or before anti-*Toxoplasma* treatment. However, 8% of cases (*n* = 9) had a fatal outcome; in four of those, the diagnosis of toxoplasmosis was made only postmortem. Prompt diagnosis and treatment can be lifesaving in immunocompetent hosts.

Our team recently identified another case of severe toxoplasmosis in a young immunocompetent patient presenting with severe community-acquired pneumonia and progressive respiratory failure after the ingestion of infected venison procured in the southern United States. The initial diagnosis of disseminated toxoplasmosis was made by plasma metagenomics cell-free DNA (Karius test) and further confirmed by serologies, PCR in bronchoalveolar lavage (BAL) and blood, and liver biopsy histopathology [71]. Initial empiric treatment with trimethoprim/sulfamethoxazole was lifesaving [71].

### 4.1. Clinical Suspicion of Toxoplasmic Pneumonia

Pulmonary toxoplasmosis in immunocompetent hosts should be suspected in patients with dyspnea, shortness of breath, cough, respiratory distress, hypoxia, respiratory failure, and the need for intubation in the absence of alternative explanation and in the absence of a response to empiric therapies.

### 4.2. Clinical Suspicion of Toxoplasmic Cardiac Involvement

Cardiac toxoplasmosis should be suspected in patients with dyspnea, acute chest pain mimicking acute myocardial infarction, palpitations, myocarditis, pericarditis, heart failure, or cardiogenic shock. The classic presentation of toxoplasmic myocarditis with heart failure shortly after an acute *Toxoplasma* infection likely represents an autoimmune mechanism rather than direct tissue damage. These cases usually have favorable outcomes and can also occasionally self-resolve even before the initiation of anti-*Toxoplasma* therapy [29,65]. Cases with more prolonged courses and frequent relapses have also been reported (usually in the absence of targeted anti-*Toxoplasma* therapy), implicating direct cardiac tissue damage from *T. gondii* in those cases [65,72]. The optimal management of toxoplasmic pericarditis/myopericarditis remains unclear, but the majority of the cases were managed with typical anti-*Toxoplasma* therapy with a favorable outcome.

### 4.3. Clinical Suspicion of Toxoplasmic CNS Involvement

CNS toxoplasmosis in immunocompetent hosts should be suspected in patients with mental status changes, confusion, agitation, delirium, personality changes, rapidly progressive dementia [61], myoclonus, and seizures with or without fever. Focal neurologic deficits may be absent. Symptoms of CNS toxoplasmosis in some patients were initially thought to be due to brain tumor, and the diagnosis of toxoplasmosis was made only after the brain lesion was surgically removed [60]. The three major clinical patterns of CNS toxoplasmosis are [73] (a) diffuse encephalopathy (with or without seizures), (b) meningoencephalitis, and (c) mass lesions (single or multiple). Cases with ADEM [74], hydrocephalus and ventriculitis [75], central facial nerve palsies [76], Guillain–Barre syndrome (GBS) [37,77], or acute polyradiculoneuropathies have also been reported. The cerebrospinal fluid (CSF) findings can show CSF pleocytosis and elevated CSF protein (up to 100 mg/dl). However, CNS toxoplasmosis should also be considered even in the absence of CSF pleocytosis, or just with mild pleocytosis [58], particularly so in the presence of elevated CSF protein [61]. Low CSF glucose has also been occasionally reported [78]. Low-level peripheral serum eosinophilia (~15%) may be seen, although rare cases with significant peripheral eosinophilia (~50%) have also been reported [51]. The majority of CNS toxoplasmosis cases responded to anti-*Toxoplasma* treatment. Rare cases with seizures were reported to have self-resolved without anti-*Toxoplasma* therapy [70]. There were also rare cases with fatal outcomes.

In some cases, the toxoplasmosis diagnosis was suspected only after the development of ocular findings or lymphadenopathy; however, these two findings were present in only ~30% and 74% of cases reporting this information.

### 4.4. Differential Diagnosis

Toxoplasmic pneumonia can mimic pulmonary edema, atypical pneumonia, or *Pneumocystis jirovecii* (PJP) pneumonia with ground glass opacities, bilateral diffuse interstitial or alveolar infiltrates, and pleural effusions. Cardiac toxoplasmosis can mimic myocarditis or myopericarditis of infectious or postinfectious etiologies, myocarditis of metabolic or oncologic causes, and acute myocardial infarction. CNS toxoplasmosis can mimic CNS lymphoma, bacterial or fungal brain abscess, CNS granulomatous diseases (e.g., tuberculosis/tuberculomas), rapid progressive neurodegenerative disease, Alzheimer’s disease, diffuse Lewy body disease, Creutzfeldt–Jakob (CJD) disease, and metabolic encephalopathy (uremic, hepatic, and hypoglycemic).

### 4.5. Risk Factors for Severe Toxoplasmosis

Factors associated with severe acute toxoplasmosis in immunocompetent hosts include the virulence of the *T. gondii* strain, the parasite load (inoculum effect), the parasite stage (oocysts vs. tissue cysts), host genetic characteristics, and/or the delay in initiation of anti-*Toxoplasma* therapy [11,42,55]. Poor host adaptation to atypical virulent *T. gondii* strains (e.g., Amazonian strains) may also explain differences in the severity of clinical manifestations [79,80,81,82,83]. Although the pathophysiologic mechanism for the increased virulence of the Amazonian strains has not been completely understood, the more virulent Amazonian strain [84] may cause more severe disease via enhanced invasiveness, interference with Th1 host immune responses [34], and dissemination to multiple organs, with lung involvement common in those cases.

Variations in the severity of the disease in common source family outbreaks [44,55] or military outbreaks among military personnel [38,39]—where the implicated *T. gondii* strains were apparently the same—support the role of the parasite load in the differential observed disease severity. A massive parasite load can overwhelm the immune response and lead to severe toxoplasmosis [55]. The transient decrease in CD4 counts [3,17,38,55,56] observed early in the course of certain cases of severe toxoplasmosis likely contributed to the observed severity. Of note, in all these cases, the initially low CD4 counts normalized after the initiation of anti-*Toxoplasma* therapy. Otherwise, there is no known drug resistance reported for *T. gondii* [85].

A detailed exposure history should always be obtained. However, the absence of classic risk factors should not exclude the diagnosis of toxoplasmosis if there is compatible clinical presentation, particularly if patients do not respond to empiric therapies. *T. gondii* is a ubiquitous parasite, and 50% of patients with acute toxoplasmosis do not report risk factors based on surveys of pregnant women who gave birth to infants with congenital toxoplasmosis [86,87]. Moreover, the fact that the severe toxoplasmosis cases reported in this systematic review came from 33 different countries further supports our hypothesis that the problem is more widespread than originally thought.

### 4.6. Diagnostic Methods

Serological tools, histopathologic examination of tissue biopsies, molecular diagnostics from blood, CSF, other body fluids or tissue biopsies, metagenomic cell-free DNA from blood and/or CSF, and direct examination for tachyzoites of body fluids (BAL, CSF, and ocular fluids) may help to establish the diagnosis of toxoplasmosis and should be promptly sent. Although serologies are the diagnostic methods of choice, physicians should be aware that in very recent acute *Toxoplasma* infections, only molecular diagnostics (from blood, other body fluids, or tissues) may be positive initially, as antibody immune responses may be delayed.

### 4.7. Prior Reviews

Prior reviews were either non-systematic reviews, had focused only on single organ systems (e.g., toxoplasmic pneumonia or CNS disease), focused only on Amazonian cases, or were published more than 1–3 decades ago [12,16,17,18,50,61]. Pomeroy et al. [16] (1992) reviewed 13 cases of severe pulmonary toxoplasmosis in immunocompetent hosts, published between 1942 and 1987. Leal et al. [17] (2007) reported six cases of pulmonary toxoplasmosis in immunocompetent hosts and Di Gassi et al. [50] (2014) reported three such cases. Graham et al. [18] (2021) reviewed seven cases of CNS toxoplasmosis in immunocompetent hosts, including four patients with fatal outcomes due to delayed presentation to the hospital, difficulty diagnosing the infection, and lack of anti-*Toxoplasma* treatment. Habek et al. [61] (2009) reviewed eight such cases. Bossi et al. [12] (2002) reviewed three cases of acute disseminated toxoplasmosis from French Guiana, acquired either from eating raw or undercooked game meat or the ingestion of oocyst-contaminated water in the forest. Jungle wild cats may play a major role in the *T. gondii* cycle in the Amazonian region. Zhou et al. (2021) [19] had reviewed six cases of cardiac toxoplasmosis in immunocompetent patients.

Our work is the most comprehensive and up-to-date systematic review on this topic. We applied rigorous criteria for the validation of the immunocompetent status of the host and the ascertainment that toxoplasmosis was the most likely explanation for the described clinical symptomatology. Our primary analysis was focused only on cases published on or after 1985 to exclude possible confounding from AIDS cases that were not properly diagnosed early during the AIDS pandemic.

Some study limitations should be acknowledged. It is possible that some cases of toxoplasmosis with prolonged illness might have been missed; however, we believe that the yield of our search for severe cases with pulmonary, CNS, or cardiac involvement, disseminated disease, or fatal outcomes was very high. Furthermore, some cases of severe toxoplasmosis in the setting of outbreaks could have been missed, although our search strategy would have captured severe outbreak cases that their respective papers mentioned in their title or abstract. Moreover, the list of cases published before 1985, included only in our secondary summary analysis, unavoidably, might have also contained some AIDS cases.

## 5. Conclusions

The diagnosis of severe toxoplasmosis in immunocompetent hosts is challenging, due to the low clinical suspicion among healthcare providers. However, cases of severe toxoplasmosis in immunocompetent hosts have been reported from many diverse countries, supporting the hypothesis that this condition is more widely encountered than previously thought and not limited to the Amazonian region. Toxoplasmosis should be considered in the differential diagnosis of immunocompetent patients who present with severe disease with pulmonary, CNS or cardiac involvement, disseminated disease with multiorgan failure, or FUO. Detailed exposure history should always be obtained. However, the classic risk factors and common manifestations of toxoplasmosis (mononucleosis-like illness, lymphadenopathy, and chorioretinitis) may not be always present; their absence thereof should not exclude the diagnosis. Anti-*Toxoplasma* treatment can be lifesaving.

## Additional Information

For assistance in the laboratory diagnosis and management of toxoplasmosis the Remington’s Lab (Jack S. Remington Laboratory for Specialty Diagnostics, Palo Alto, CA, USA) can be contacted at https://www.sutterhealth.org/services/lab-pathology/toxoplasma-serology-laboratory, remingtonlab@sutterhealth.org, 650-853-4828 (Pacific Time) for regular hours, and 650-438-4427 cell phone (JG Montoya) for urgent questions/consults.

## Figures and Tables

**Table 1:** Characteristics of severe toxoplasmosis cases in immunocompetent hosts published between 1985 and 2022.

	N (or N/Total; %)
**Articles**	82
**N of severe toxoplasmosis cases**	117
**Ages**	
Adults	98/117 (84%)
Pediatric	19/117 (16%)
**Top 5/33 countries** (where infection most likely acquired) *	
French Guiana	23/117 (20%)
France	17/117 (15%)
Colombia	12/117 (9%)
India	10/117 (9%)
Brazil	8/117 (7%)
**Duration of symptoms prior to admission** (among those reporting)	**N cases/Total reporting this information (%)**
*All*	
<3 weeks (acute)	62/83 (75%)
<3 months (acute or subacute)	77/83 (93%)
*Pulmonary*	
<3 weeks (acute)	25/31 (81%)
<3 months (acute or subacute)	30/31 (97%)
*CNS*	
<3 weeks (acute)	21/32 (66%)
<3 months (acute or subacute)	29/32 (91%)
*Cardiac*	
<3 weeks (acute)	20/23 (87%)
<3 months (acute or subacute)	23/23 (100%)
*Disseminated*	
<3 weeks (acute)	11/13 (85%)
<3 months (acute or subacute)	13/13 (100%)
**Diagnostic methods**	
Serology	111/117 (95%)
Molecular	22/117 (19%)
Histopathologic	24/117 (21%)
More than one diagnostic method	98/117 (84%)
Based on response to anti-*Toxoplasma* therapy	88/110 (80%) (unclear for 7)
**Type of infection** (cases)	
Acute Primary Toxoplasmosis	98/117 (84%)
Unclear	19/117 (16%)
**Type of implicated *T. gondii* strains genotyped in** (strains)	
Non-type II atypical strains	22/23 (96%)
Type II strain	1/23 (4%)
No genotyping/Not reporting genotyping data	94/117 (80%)
**Reporting risk factor**	60/117 (51%)
**Risk factors**	
Eating raw or undercooked meat or eating game meat	28/60 (47%)
Living in high prevalence area (e.g., French Guiana)	23/60 (38%)
Drinking untreated water	22/60 (37%)
Contact with cats	14/60 (23%)
Travel to high prevalence area (Central/South America/Amazonian region)	8/60 (13%)
Contact with soil (gardening)	2/60 (3%)
Multiple risk factors	19/60 (32%)
Not reported risk factors	57/117 (49%)
**Outbreaks of severe toxoplasmosis cases** (among those reporting risk factors)	
Military personnel outbreak	13/60 (22%)
Family outbreak (e.g., outbreak during a humanitarian mission in Venezuela, or in family in the US returning from international Travel)	2/60 (3%)

Footnote: * Imputed the country of authors for country of infection when country of infection was not reported (shown with *).

**Table 2:** Manifestations of severe toxoplasmosis cases in immunocompetent hosts.

Clinical Manifestations of Severe Toxoplasmosis	N Cases/Total Applicable Cases (%)
***Pulmonary*** *^a^*	51/117 (44%)
Pneumonia	48/51 (94%)
Respiratory failure	24/51 (47%)
***CNS*** *^a^*	46/117 (39%)
Encephalitis	25/46 (54%)
Meningitis	6/46 (13%)
Focal Neurologic findings	11/46 (24%)
Cranial nerve palsies	8/46 (17%)
Guillain Barre syndrome	3/46 (7%)
Spinal lesion	1/46 (2%)
***Cardiac*** *^a^*	36/117 (31%)
Myocarditis	27/36 (75%)
Pericarditis	18/36 (50%)
Heart failure	7/36 (19%)
Arrhythmia	8/36 (22%)
***Disseminated disease*** *^b^*	28/117 (24%)
***FUO > 3 months*** *^c^*	2/117 (2%)
More than one organ system involved	31/117 (27%)
**Severity of Illness** (among those reporting)	
Critical Illness	44/90 (49%)
ICU admission	29/54 (54%)
Fatal Outcome	9/110 (8%)
**Outcomes** (among those reporting)	
Improvement/resolution (at last follow up)	88/110 (80%)
Resolution without or before anti-*Toxoplasma* treatment	13/110 (12%)
Fatal outcome	9/110 (8%)
**Time to resolution** (among those reporting)	
Resolution <4 weeks from treatment	23/36 (64%)

Abbreviations: CNS: Central nervous system, FUO: Fever of unknown origin; GBS: Guillain–Barre Syndrome; ICU: Intensive care unit. ^a^ More than one clinical manifestation per organ system could be present (e.g., encephalitis and focal neurologic findings). Total sum per category exceeds 100%. ^b^ Disseminated disease with pulmonary involvement: 82% (23/28) of cases. Disseminated disease with cardiac involvement: 50% (14/28) of cases. Disseminated disease with both cardiac and pulmonary involvement: 32% (9/28) of cases. Disseminated disease with CNS involvement: 18% (5/28) of cases. More than one organ was involved in 26% (31/117) of cases. ^c^ FUO for 5 months 1 case, and FUO for 6 months 1 case.

**Table 3:** Types of imaging findings.

	N Cases/Total Applicable Cases Reporting This Information (%)
**Lung Imaging Findings** ^a^ (reported in 75% (38/51) of pulmonary cases)	
Diffuse bilateral infiltrates (ground glass opacities, interstitial/alveolar infiltrates)	31/38 (82%)
Nodular lesions/opacities	5/38 (13%)
Focal localized consolidations	6/38 (16%)
Pleural effusions	18/38 (47%)
Mediastinal LADP	4/38 (11%)
**CNS Imaging Findings** ^a^ (reported in 41/46 CNS cases)	
*By appearance*	
Abscess like/mass-like focal lesions	21/41 (51%)
Diffuse encephalitis	7/41 (17%)
Meningitis	4/41 (10%)
Meningoencephalitis	2/41 (5%)
*By location*	
Focal supratentorial lesions	28/41 (68%)
Focal infratentorial lesions	3/41 (7%)
Spinal lesions	2/41 (5%)
**Cardiac Imaging Findings** ^a^ (reported in 34/36 cardiac cases)	
Myocarditis	23/34 (68%)
Pericarditis	16/34 (47%)
Myopericarditis	11/34 (34%)
Low cardiac function/heart failure	13/34 (45%)
Cardiac rhythm abnormalities	10/34 (29%)

Footnote ^a^ More than one imaging finding could have been present per toxoplasmosis case-category (total sum per category exceeds 100%).

**Table 4:** Compilation list of imaging findings.

Imaging Findings	
**Lung imaging findings** *	Diffuse ground glass opacities (GGO) Pulmonary interstitial infiltrates (unilateral or bilateral)/interstitial syndrome /interstitial pneumonia Reticular/alveolar infiltrates Peribroncho-vascular thickening Septal thickening Pulmonary nodules/nodular opacities (multiple, unilateral or bilateral) Nodular cavities Focal areas of consolidation (unilateral) Atelectasis Paratracheal/mediastinal lymphadenopathy Pleural effusions (large or small, bilateral or unilateral) Diffuse pulmonary infiltrates and/or mediastinal lymphadenopathy intensely metabolically active in PET scan.
**CNS imaging findings** ^£^	Multifocal white matter and/or grey matter lesions T2 hyperintense lesions/T1 hypointense lesions (unifocal or multifocal), in the frontoparietal, temporal, periventricular region, basal ganglia, pons or corticomedullary junction ^€^ FLAIR high signal lesions Ring-enhancing lesions (single or multiple, unilateral or bilateral) Peripherally enhancing lesions (with or without central diffusion restriction in DWI sequences) Open ring-enhancing lesions Mass-like lesions/abscess-like lesions/ granuloma-like lesions/ tuberculoma-like lesions Micro-abscess like lesions (with perilesional edema) Mushroom-shaped lesions (with irregular rim enhancement) Perivascular enhancing lesions with vasogenic edema and adjacent hemorrhagic focus Midline shift ADEM-like lesions Cerebellar lesions/ rhombencephalitis Hydrocephalus/ Ventriculitis Leptomeningeal enhancement Brain calcifications Intraspinal/intramedullary ring enhancing lesion Normal brain MRI (e.g., case of NMDA encephalitis associated with acute toxoplasmosis)
**Cardiac imaging findings** ^±^	Myocarditis picture with late gadolinium enhancement; subepicardial enhancement and/or myocardial necrosis in cardiac MRI Pericarditis findings with hyperechoic pericardium/ pericardial effusions (small or large, with or without cardiac tamponade) in cardiac MRI Cardiomegaly Cardiac function with poor contractility; hypokinesia of ventricular walls; low ejection fraction, heart failure EKG with AV block, AV dissociation, bundle branch block; tachyarrhythmia, ventricular fibrillation

Abbreviations: ADEM: Acute disseminated encephalomyelitis; AV: Atrioventricular; EKG: Electrocardiogram; DWI: Diffusion weighted images; FLAIR-MRI: Fluid attenuated inversion recovery axial magnetic resonance imaging; LADP: Lymphadenopathy; MRI: Magnetic resonance imaging; anti-NMDA-encephalitis: Anti N methyl D aspartate receptor encephalitis. * The pulmonary imaging findings can mimic pulmonary edema/congestive heart failure, atypical pneumonia, PJP pneumonia, and lymphangitis. ^£^ The CNS imaging findings can mimic CNS lymphoma, bacterial or fungal brain abscess, CNS tuberculosis/tuberculomas, rapid progressive neurodegenerative disease, Alzheimer’s disease, diffuse Lewy body disease, Creutzfeldt–Jakob (CJD) disease, and metabolic encephalopathy. ^€^ There is a predilection for the basal ganglia [57,58], but lesions in all brain parts, including in the spinal cord (more rarely), have been reported. The intensity of contrast enhancement correlates with the leukocyte count and is more intense in immunocompetent patients [18]. ^±^ The cardiac imaging findings can mimic myocarditis, pericarditis from infectious, postinfectious, metabolic oncologic causes, or acute myocardial infarction.

## Data Availability

Individual study-level data are available upon request by the corresponding author (Contopoulos-Ioannidis, email: dcontop@stanford.edu).

## Appendix A

Search strategies and PRISMA checklist

**Strategy 1**: “Toxoplasmosis AND “Severe OR Fatal OR Lethal OR Disseminated OR Fulminant OR multisystem OR multiorgan OR Shock” AND (immunocompetent OR healthy OR not immunocompromised).

**Strategy 2**: “(toxoplasmosis OR toxoplasma gondii OR t.gondii OR toxoplasm*) AND (pulmonary OR lung OR myocarditis OR myopericarditis OR encephalitis OR brain OR cerebral OR central nervous system OR intracrania* OR intra-cranial OR seizures OR mental status changes OR myelopathy OR spinal cord OR acute disseminated encephalomyelitis OR ADEM OR hemophagocytic OR hepatitis OR nephritis OR nephrotic OR myositis OR pyomyositis OR complications OR atypical OR unusual) AND (immunocompetent OR non immunocompromised OR not immunocompromised OR healthy OR without immunosupression)”.

**Strategy 3**: “(toxoplasmosis OR toxoplasma gondii OR t.gondii OR toxoplasm*) AND (pulmonary OR lung OR myocarditis OR myopericarditis OR encephalitis OR brain OR cerebral OR central nervous system OR intracrania* OR intra-cranial OR seizures OR mental status changes OR myelopathy OR spinal cord OR acute disseminated encephalomyelitis OR ADEM OR hemophagocytic OR hepatitis OR nephritis OR nephrotic OR myositis OR pyomyositis OR complications OR atypical OR unusual) AND (child (ti) OR adult (ti) OR woman (ti) OR man (ti) OR patient(ti))”.

**PRISMA Checklist**

**Table:**

Section and Topic	Item #	Checklist Item	Location Where Item Is Reported
**TITLE**			
Title	1	Identify the report as a systematic review.	Page 1
**ABSTRACT**			
Abstract	2	See the PRISMA 2020 for Abstracts checklist.	Page 1–2
**INTRODUCTION**			
Rationale	3	Describe the rationale for the review in the context of existing knowledge.	Page 2–3
Objectives	4	Provide an explicit statement of the objective(s) or question(s) the review addresses.	Page 2–3
**METHODS**			
Eligibility criteria	5	Specify the inclusion and exclusion criteria for the review and how studies were grouped for the syntheses.	Page 4–5
Information sources	6	Specify all databases, registers, websites, organisations, reference lists and other sources searched or consulted to identify studies. Specify the date when each source was last searched or consulted.	Page 3
Search strategy	7	Present the full search strategies for all databases, registers and websites, including any filters and limits used.	Page 4 (Appendix A, Page 18)
Selection process	8	Specify the methods used to decide whether a study met the inclusion criteria of the review, including how many reviewers screened each record and each report retrieved, whether they worked independently, and if applicable, details of automation tools used in the process.	Page 3
Data collection process	9	Specify the methods used to collect data from reports, including how many reviewers collected data from each report, whether they worked independently, any processes for obtaining or confirming data from study investigators, and if applicable, details of automation tools used in the process.	Page 5
Data items	10a	List and define all outcomes for which data were sought. Specify whether all results that were compatible with each outcome domain in each study were sought (e.g., for all measures, time points, analyses), and if not, the methods used to decide which results to collect.	Page 5
	10b	List and define all other variables for which data were sought (e.g., participant and intervention characteristics, funding sources). Describe any assumptions made about any missing or unclear information.	Page 5
Study risk of bias assessment	11	Specify the methods used to assess risk of bias in the included studies, including details of the tool(s) used, how many reviewers assessed each study and whether they worked independently, and if applicable, details of automation tools used in the process.	N/A
Effect measures	12	Specify for each outcome the effect measure(s) (e.g., risk ratio, mean difference) used in the synthesis or presentation of results.	N/A
Synthesis methods	13a	Describe the processes used to decide which studies were eligible for each synthesis (e.g., tabulating the study intervention characteristics and comparing against the planned groups for each synthesis (item #5)).	N/A
	13b	Describe any methods required to prepare the data for presentation or synthesis, such as handling of missing summary statistics, or data conversions.	N/A
	13c	Describe any methods used to tabulate or visually display results of individual studies and syntheses.	N/A
	13d	Describe any methods used to synthesize results and provide a rationale for the choice(s). If meta-analysis was performed, describe the model(s), method(s) to identify the presence and extent of statistical heterogeneity, and software package(s) used.	N/A
	13e	Describe any methods used to explore possible causes of heterogeneity among study results (e.g., subgroup analysis, meta-regression).	N/A
	13f	Describe any sensitivity analyses conducted to assess robustness of the synthesized results.	N/A
Reporting bias assessment	14	Describe any methods used to assess risk of bias due to missing results in a synthesis (arising from reporting biases).	N/A
Certainty assessment	15	Describe any methods used to assess certainty (or confidence) in the body of evidence for an outcome.	N/A
**RESULTS**			
Study selection	16a	Describe the results of the search and selection process, from the number of records identified in the search to the number of studies included in the review, ideally using a flow diagram.	Page 3
	16b	Cite studies that might appear to meet the inclusion criteria, but which were excluded, and explain why they were excluded.	N/A
Study characteristics	17	Cite each included study and present its characteristics.	Appendix B, page 21–25, Appendix C-Table A10, page 54–55
Risk of bias in studies	18	Present assessments of risk of bias for each included study.	N/A
Results of individual studies	19	For all outcomes, present, for each study: (a) summary statistics for each group (where appropriate) and (b) an effect estimate and its precision (e.g., confidence/credible interval), ideally using structured tables or plots.	N/A
Results of syntheses	20a	For each synthesis, briefly summarize the characteristics and risk of bias among contributing studies.	N/A
	20b	Present results of all statistical syntheses conducted. If meta-analysis was done, present for each the summary estimate and its precision (e.g., confidence/credible interval) and measures of statistical heterogeneity. If comparing groups, describe the direction of the effect.	N/A
	20c	Present results of all investigations of possible causes of heterogeneity among study results.	N/A
	20d	Present results of all sensitivity analyses conducted to assess the robustness of the synthesized results.	N/A
Reporting biases	21	Present assessments of risk of bias due to missing results (arising from reporting biases) for each synthesis assessed.	N/A
Certainty of evidence	22	Present assessments of certainty (or confidence) in the body of evidence for each outcome assessed.	N/A
**DISCUSSION**			
Discussion	23a	Provide a general interpretation of the results in the context of other evidence.	Page 14–15
	23b	Discuss any limitations of the evidence included in the review.	Page 17
	23c	Discuss any limitations of the review processes used.	Page 17
	23d	Discuss implications of the results for practice, policy, and future research.	Page 17
**OTHER INFORMATION**			
Registration and protocol	24a	Provide registration information for the review, including register name and registration number, or state that the review was not registered.	N/A
	24b	Indicate where the review protocol can be accessed, or state that a protocol was not prepared.	N/A
	24c	Describe and explain any amendments to information provided at registration or in the protocol.	N/A
Support	25	Describe sources of financial or non-financial support for the review, and the role of the funders or sponsors in the review.	Page 17
Competing interests	26	Declare any competing interests of review authors.	Page 18
Availability of data, code and other materials	27	Report which of the following are publicly available and where they can be found: template data collection forms; data extracted from included studies; data used for all analyses; analytic code; any other materials used in the review.	Page 18

## Appendix B

**References of Included Articles (1985–2022) (Primary Analysis).**

1. Abhilash KP, Roshine MK, Vandana K, Varghese GM. A probable case of acquired toxoplasmosis presenting as pyrexia of unknown origin in an immunocompetent individual. Int J Infect Dis. 2013 Nov;17(11):e1067-8. doi: 10.1016/j.ijid.2013.03.024. Epub 2013 May 28. PMID: 23726282.
2. Aksoy A, Tanir G, Ozkan M, Oguz M, Yildiz YT. Acute disseminated encephalomyelitis associated with acute Toxoplasma gondii Infection. Pediatr Neurol. 2013 Mar;48(3):236-9. doi: 10.1016/j.pediatrneurol.2012.11.004. PMID: 23419476.
3. Akturk HK, Sotello D, Ameri A, Abuzaid AS, Rivas AM, Vashisht P. Toxoplasma Infection in an Immunocompetent Host: Possible Risk of Living with Multiple Cats. Cureus. 2017 Mar 19;9(3):e1103. doi: 10.7759/cureus.1103. PMID: 28435763; PMCID: PMC5398893.
4. Alapatt JP, Kutty RK, Jose B, Gopi P. A case of cerebral toxoplasmosis in a pregnant non-immunocompromised patient. Neurol Neurochir Pol. 2009 Jul-Aug;43(4):391-5. PMID: 19742399.
5. Azarpira, Negar; Torabineghad, Simin; Rad, Hanieh; Taghipour, Musa Ventriculitis: A Case of Cerebral Toxoplasmosis in Immunocompetent Child. Neurosurgery Quarterly: August 2014 - Volume 24 - Issue 3 - p 159-160 doi: 10.1097/WNQ.0b013e31828cc5e1
6. Basit KA, Nasir S, Vohra E, Shazlee MK. Toxoplasmosis in an Immunocompetent Patient. Pak J Med Sci. 2018 Nov-Dec;34(6):1579-1581. doi: 10.12669/pjms.346.15016. PMID: 30559827; PMCID: PMC6290221.
7. Beltrame A, Venturini S, Crichiutti G, Meroni V, Buonfrate D, Bassetti M. Recurrent seizures during acute acquired toxoplasmosis in an immunocompetent traveller returning from Africa. Infection. 2016 Apr;44(2):259-62. doi: 10.1007/s15010-015-0821-7. Epub 2015 Jul 14. PMID: 26168861.
8. Bhattarai, Kumud, Anjali Bisht, Bhawesh Thapa and Ananta Bhakta Uprety. Toxoplasma gondii Pneumonia in an Immunocompetent Host: A Case Report. JNMA: Journal of the Nepal Medical Association 59 (2021): 84 - 87.
9. Bossi P, Paris L, Caumes E, Katlama C, Danis M, Bricaire F. Severe acute disseminated toxoplasmosis acquired by an immunocompetent patient in French Guiana. Scand J Infect Dis. 2002;34(4):311-4. doi: 10.1080/00365540110080124. PMID: 12064700.
10. Bossi, Philippe & Caumes, Eric & Paris, Luc & Darde, Marie Laure & Bricaire, Francois. (1999). Toxoplasma gondii-Associated Guillain-Barre’ Syndrome in an Immunocompetent Patient. Journal of clinical microbiology. 36. 3724-5. 10.1128/JCM.36.12.3724-3725.1998.
11. Bousquet A, Bigaillon C, Dumitrescu N, Larréché S, Godreuil C, Mestiri R, Le Caruyer N, Mérens A, Andriamanantena D. Acute myocarditis in an immunocompetent young man: Don’t forget Toxoplasma gondii. Int J Cardiol. 2016 Jul 1;214:358-9. doi: 10.1016/j.ijcard.2016.03.155. Epub 2016 Apr 1. PMID: 27085128.
12. Cai X, Zhou H, Xie Y, Yu D, Wang Z, Ren H. Anti-N-methyl-D-aspartate receptor encephalitis associated with acute Toxoplasma gondii infection: A case report. Medicine (Baltimore). 2018 Feb;97(7):e9924. doi: 10.1097/MD.0000000000009924. PMID: 29443773; PMCID: PMC5839864.
13. Calore EE, Minkovski R, Khoury Z, Seguro AC, Perez Calore NM, Cavaliere MJ. Skeletal muscle pathology in 2 siblings infected with Toxoplasma gondii. J Rheumatol. 2000 Jun;27(6):1556-9. PMID: 10852291.
14. Candolfi E, de Blay F, Rey D, Christmann D, Treisser A, Pauli G, Kien T. A parasitologically proven case of Toxoplasma pneumonia in an immunocompetent pregnant woman. J Infect. 1993 Jan;26(1):79-81. doi: 10.1016/0163-4453(93)96968-v. PMID: 8454890.
15. Chandenier J, Jarry G, Nassif D, Douadi Y, Paris L, Thulliez P, Bourges-Petit E, Raccurt C. Congestive heart failure and myocarditis after seroconversion for toxoplasmosis in two immunocompetent patients. Eur J Clin Microbiol Infect Dis. 2000 May;19(5):375-9. doi: 10.1007/s100960050498. PMID: 10898141.
16. Chang HJ, Hong CT, Hu CJ, Chan L. Central nervous system toxoplasmosis in an immunocompetent individual. Kaohsiung J Med Sci. 2019 Apr;35(4):248-249. doi: 10.1002/kjm2.12036. Epub 2019 Mar 19. PMID: 30887680.
17. Chiappe Gonzalez AJ, Vargas Matos I, Massucco Revoredo V. Acute toxoplasmosis complicated with myopericarditis and possible encephalitis in an immunocompetent patient. IDCases. 2020 Apr 24;20:e00772. doi: 10.1016/j.idcr.2020.e00772. PMID: 32395428; PMCID: PMC7210424.
18. Cortés AD, Aguirre N. Severe disseminated acute toxoplasmosis in an adult immunocompetent patient from the Colombian Pacific. Biomedica. 2018 Aug 1;38(0):19-23. doi: 10.7705/biomedica.v38i0.4087. PMID: 30184374.
19. Cortés DA, Aguilar MC, Rios HA, Rodriguez FJ, Montes KV, Gómez-Marín JE,, de-la-Torre A. Severe acute multi-systemic failure with bilateral ocular toxoplasmosis in immunocompetent patients from urban settings in Colombia: Case reports. Am J Ophthalmol Case Rep. 2020 Mar 13;18:100661. doi: 10.1016/j.ajoc.2020.100661. PMID: 32195446; PMCID: PMC7078491.
20. Cottrell AJ. Acquired toxoplasma encephalitis. Arch Dis Child. 1986 Jan;61(1):84-5. doi: 10.1136/adc.61.1.84. PMID: 3954425; PMCID: PMC1777557.
21. Couvreur J, Thulliez P. Toxoplasmose acquise à localisation oculaire ou neurologique: 49 cas [Acquired toxoplasmosis of ocular or neurologic site: 49 cases]. Presse Med. 1996 Mar 16;25(9):438-42. French. PMID: 8685192.
22. Darde’ ML, Villena I, Pinon JM, Beguinot I. Severe toxoplasmosis caused by a Toxoplasma gondii strain with a new isoenzyme type acquired in French Guyana. J Clin Microbiol. 1998 Jan;36(1):324. doi: 10.1128/JCM.36.1.324-324.1998. PMID: 9431981; PMCID: PMC124868.
23. De Salvador-Guillouet F, Ajzenberg D, Chaillou-Opitz S, Saint-Paul MC, Dunais B, Dellamonica P, Marty P. Severe pneumonia during primary infection with an atypical strain of Toxoplasma gondii in an immunocompetent young man. J Infect. 2006 Aug;53(2):e47-50. doi: 10.1016/j.jinf.2005.10.026. Epub 2005 Dec 15. PMID: 16352339.
24. Demar M, Ajzenberg D, Maubon D, Djossou F, Panchoe D, Punwasi W, Valery N, Peneau C, Daigre JL, Aznar C, Cottrelle B, Terzan L, Darde’ ML, Carme B. Fatal outbreak of human toxoplasmosis along the Maroni River: epidemiological, clinical, and parasitological aspects. Clin Infect Dis. 2007 Oct 1;45(7):e88-95. doi: 10.1086/521246. Epub 2007 Aug 27. PMID: 17806043.
25. Demar M, Hommel D, Djossou F, Peneau C, Boukhari R, Louvel D, Bourbigot AM, Nasser V, Ajzenberg D, Darde ML, Carme B. Acute toxoplasmoses in immunocompetent patients hospitalized in an intensive care unit in French Guiana. Clin Microbiol Infect. 2012 Jul;18(7):E221-31. doi: 10.1111/j.1469-0691.2011.03648.x. Epub 2011 Sep 29. PMID: 21958195.
26. Desguerre I, Pedespan JM, Buissonnie’re, Couvreur J, Ponsot G. [Acquired cerebral toxoplasmosis in an non-immunosuppressed child]. Arch Fr Pediatr. 1993 Apr;50(4):339-42. French. PMID: 8379823.
27. de Souza Giassi K, Costa AN, Apanavicius A, Teixeira FB, Fernandes CJ, Helito AS, Kairalla RA. Tomographic findings of acute pulmonary toxoplasmosis in immunocompetent patients. BMC Pulm Med. 2014 Nov 25;14:185. doi: 10.1186/1471-2466-14-185. PMID: 25420956; PMCID: PMC4254211.28. Duffield JS, Jacob AJ, Miller HC. Recurrent, life-threatening atrioventricular dissociation associated with toxoplasma myocarditis. Heart. 1996 Nov;76(5):453-4. doi: 10.1136/hrt.76.5.453. PMID: 8944597; PMCID: PMC484583.
29. Filipowicz A, Coca MN, Blair BM, Chang PY. Acute myocarditis with cardiogenic shock and multiple organ failure, followed by bilateral panuveitis masquerading as endogenous endophthalmitis, due to *Toxoplasma gondii* in an immunocompetent patient. Retin Cases Brief Rep. 2021 Sep 1;15(5):575-580. doi: 10.1097/ICB.0000000000000855. PMID: 30664080.
30. Galli-Tsinopoulou A, Kyrgios I, Giannopoulou EZ, Gourgoulia S, Maggana I, Katechaki E, Chatzidimitriou D, Evangeliou AE. Acquired toxoplasmosis accompanied by facial nerve palsy in an immunocompetent 5-year-old child. J Child Neurol. 2010 Dec;25(12):1525-8. doi: 10.1177/0883073810370480. PMID: 21148450.
31. Groh M, Faussart A, Villena I, Ajzenberg D, Carme B, Demar M, Joly V, Houze S, Simon S, Aubert D, Charlois-Ou C, Yeni P. Acute lung, heart, liver, and pancreatic involvements with hyponatremia and retinochoroiditis in a 33-year-old French Guianan patient. PLoS Negl Trop Dis. 2012;6(10):e1802. doi: 10.1371/journal.pntd.0001802. Epub 2012 Oct 25. PMID: 23145185; PMCID: PMC3493371.
32. Gupta A, Raja A, Mahadevan A, Shankar SK. Toxoplasma granuloma of brainstem: a rare case. Neurol India. 2008 Apr-Jun;56(2):189-91. PMID: 18688147.
33. Guerot E, Assayag P, Morgant C, Hess M, ValÃ¨re PE. Manifestations pericardiques de la toxoplasmose [Pericardial manifestations of toxoplasmosis]. Arch Mal Coeur Vaiss. 1992 Jan;85(1):109-11. French. PMID: 1550430.
34. Habek M, Ozretić D, Zarković K, Djaković V, Mubrin Z. Unusual cause of dementia in an immunocompetent host: toxoplasmic encephalitis. Neurol Sci. 2009 Feb;30(1):45-9. doi: 10.1007/s10072-008-0007-5. Epub 2009 Jan 16. PMID: 19148571.
35. Harbada RK, Sorabjee JS, Surya N, Jadhav KA, Mirgh S. Cerebellar Toxoplasmosis in an Immunocompetent Patient with G6PD Deficiency. J Assoc Physicians India. 2016 Aug;64(8):79-82. PMID: 27762116.
36. Henao-Martinez AF, Franco-Paredes C, Palestine AG, Mon-toya JG. Symptomatic acute toxoplasmosis in returning travelers. Open Forum Infect Dis 2018;5:ofy058.
37. Hoti YU, Aziz A, Ishaque K, Abbas S, Ud Din TS. Intra-cranial Toxoplasmosis in an Immunocompetent Female. J Coll Physicians Surg Pak. 2016 Jun;26(6 Suppl):S39-41. PMID: 27376217.
38. IñíguezC, JericÃ³ I, Pascual LF, Cuesta J, Morales F. Síndrome de Miller-Fisher asociado a infección por toxoplasma [Miller-Fisher syndrome associated with toxoplasmosis]. Neurologia. 1997 Apr;12(4):172-4. Spanish. PMID: 9235026.
39. Jimenez-Caballero PE, Serviá M, Marsal-Alonso C. Encefalomielitis aguda diseminada por toxoplasmosis en un paciente de edad avanzada [Acute disseminated encephalomyelitis due to toxoplasmosis in an elderly patient]. Rev Neurol. 2010 Apr 1;50(7):445-7. Spanish. PMID: 20387217.
40. Kanno A, Suzuki Y, Minami M, Ogawa K, Oishi M, Kamei S. A healthy, 81-year-old woman with toxoplasmic encephalitis. Geriatr Gerontol Int. 2012 Oct;12(4):759-61. doi: 10.1111/j.1447-0594.2012.00863.x. PMID: 22998383.
41. Kaushik RM, Mahajan SK, Sharma A, Kaushik R, Kukreti R. Toxoplasmic meningoencephalitis in an immunocompetent host. Trans R Soc Trop Med Hyg. 2005 Nov;99(11):874-8. doi: 10.1016/j.trstmh.2005.06.017. PMID: 16111730.
42. Leal FE, Cavazzana CL, de Andrade HF Jr, Galisteo A Jr, de Mendonça JS, Kallas EG. Toxoplasma gondii pneumonia in immunocompetent subjects: case report and review. Clin Infect Dis. 2007 Mar 15;44(6):e62-6. doi: 10.1086/511871. Epub 2007 Feb 8. PMID: 17304443.
43. Leroy J, Houzé S, Dardé ML, Yéra H, Rossi B, Delhaes L, Gabriel F, Loubet P, Deleplancque AS, Senneville E, Ajana F, Sendid B, Malvy D. Severe toxoplasmosis imported from tropical Africa in immunocompetent patients: A case series. Travel Med Infect Dis. 2020 May-Jun;35:101509. doi: 10.1016/j.tmaid.2019.101509. Epub 2019 Nov 9. PMID: 31712179.
44. Lescop J, Brinquin L, Schill H, Soulié D, Sarrazin JL, Cordoliani YS. Encéphalite toxoplasmique diffuse chez un sujet non immunodéprimé [Diffuse toxoplasmic encephalitis in a non-immunosuppressed patient]. J Radiol. 1995 Jan;76(1):21-4. French. PMID: 7861364.
45. Lesenne M, Asseman P, Fortier B, de Groote P, Bauchart JJ, Millaire A, Haftel Y, Thery C. Myocardite aiguë à toxoplasme simulant un infarctus. A propos d’un cas [Acute myocarditis caused by toxoplasmosis simulating infarction. Apropos of a case] Arch Mal Coeur Vaiss. 1996 Jul;89(7):923-5. French. PMID: 8869256.
46. Li ZH, Guo FY, Wang ZQ, Cui J. Intracranial inflammatory granuloma caused by toxoplasmosis. Pathog Glob Health. 2014 Jul;108(5):255-9. doi: 10.1179/2047773214Y.0000000147. PMID: 25175876; PMCID: PMC4153827.
47. Lima KDF, Queiroz ALG, Teixeira HS, Bonsi VM, Inada BSY, Lancellotti CLP, Ba êta AM. An atypical case of neurotoxoplasmosis in immunocompetent patient. Radiol Case Rep. 2021 May 1;16(7):1766-1769. doi: 10.1016/j.radcr.2021.04.013. PMID: 34007399; PMCID: PMC8111245.
48. Luis Eduardo Pino, Jorge Enrique Salinas, Myriam Consuelo LÃ³pez, Descripcion de un brote epidemico de toxoplasmosis aguda en pacientes inmunocompetentes miembros de las fuerzas militares de Colombia durante operaciones de selva, Infectio, Volume 13, Issue 2, 2009, Pages 83-91, ISSN 0123-9392, https://doi.org/10.1016/S0123-9392(09)70729-5.
49. Lyngberg KK, Vennervald BJ, Bygbjerg IC, Hansen TM, Thomsen OO: Toxoplasma pericarditis mimicking systemic lupus erythrematous: Diagnostic and treatment difficulties in one patient. Ann Med 1992; 24: 337-340
50. Lévêque MF, Chiffré D, Galtier C, Albaba S, Ravel C, Lachaud L, Iemmi A, Flori P, Sterkers Y. Molecular diagnosis of toxoplasmosis at the onset of symptomatic primary infection: A straightforward alternative to serological examinations. Int J Infect Dis. 2019 Feb;79:131-133. doi: 10.1016/j.ijid.2018.11.368. Epub 2018 Dec 4. PMID: 30529368.
51. Manwani N, Ravikumar K, Viswanathan V, Rao SM, Mahadevan A. Acquired Toxoplasmosis Presenting with a Brainstem Granuloma in an Immunocompetent Adolescent. Indian Pediatr. 2016 Feb;53(2):159-61. doi: 10.1007/s13312-016-0813-4. PMID: 26897153.
52. Mariani M, Pagani M, Inserra C, De Servi S. Complete atrioventricular block associated with toxoplasma myocarditis. Europace. 2006 Mar;8(3):221-3. doi: 10.1093/europace/euj046. Epub 2006 Feb 13. PMID: 16627444.
53. Martinot M, Greigert V, Farnarier C, Darde ML, Piperoglou C, Mohseni-Zadeh M, Tarabeux J, Guffroy A, Villard O, Vely F. Spinal cord toxoplasmosis in a young immunocompetent patient. Infection. 2020 Apr;48(2):299-302. doi: 10.1007/s15010-019-01380-9. Epub 2019 Dec 9. PMID: 31820319.
54. McCabe, Remington Rev Infect Dis 1987 (Ref 20) Clinical Spectrum of 107 cases of toxoplasmic lymphadenopathy
55. Michel O. Thorax 1986 (ref 18) Acute pulmonary toxoplasmosis with alveolitis of T suppressor lymphocyte type (dci got pdf)
56. Micheli R, Perini A, Duse M. Hemidystonia secondary to acquired toxoplasmosis in a non-immunodeficient patient. Eur J Pediatr. 1994 Oct;153(10):731-3. doi: 10.1007/BF01954489. PMID: 7813530.
57. Montoya JG, Jordan R, Lingamneni S, Berry GJ, Remington JS. Toxoplasmic myocarditis and polymyositis in patients with acute acquired toxoplasmosis diagnosed during life. Clin Infect Dis. 1997 Apr;24(4):676-83. doi: 10.1093/clind/24.4.676. PMID: 9145743.
58. Mustafa K, Hillyard J, Nowak E, Slowikowski J, Okogbue I, Garner D. Toxoplasma myocarditis: An atypical case in an immunocompetent patient. IDCases. 2021 Sep 8;26:e01273. doi: 10.1016/j.idcr.2021.e01273. PMID: 34584844; PMCID: PMC8450265.
59. Nelwan EJ, Shakinah S, Clarissa G, Hosea FN, Herdanto DY, Pandelaki J. Rare cardiac complication of toxoplasmosis in immunocompetent host. IDCases. 2022 Jun 15;29:e01533. doi: 10.1016/j.idcr.2022.e01533. PMID: 35756700; PMCID: PMC9218370.
60. Neves Ede S, Kropf A, Bueno WF, Bonna IC, Curi AL, Amendoeira MR, Fernandes Filho O. Disseminated toxoplasmosis: an atypical presentation in an immunocompetent patient. Trop Doct. 2011 Jan;41(1):59-60. doi: 10.1258/td.2010.100228. Epub 2010 Nov 9. PMID: 21062937.
61. Nunura J, Va’squez T, Endo S, Salazar D, Rodriguez A, Pereyra S, Solis H. Disseminated toxoplasmosis in an immunocompetent patient from Peruvian Amazon. Rev Inst Med Trop Sao Paulo. 2010 Mar-Apr;52(2):107-10. doi: 10.1590/s0036-46652010000200008. PMID: 20464132.
62. Oseroff A. Toxoplasmosis associated with nephrotic syndrome in an adult. South Med J. 1988 Jan;81(1):95-6. doi: 10.1097/00007611-198801000-00022. PMID: 3336807.
63. Paruthikunnan S, Shankar B, Kadavigere R, Prabhu M, Narayanan R, Jain H. An unusual case of disseminated toxoplasmosis in a previously healthy pregnant patient: radiographic, CT, and MRI findings. Jpn J Radiol. 2014 Nov;32(11):664-9. doi: 10.1007/s11604-014-0352-7. Epub 2014 Aug 24. PMID: 25151528.
64. Paspalaki PK, Mihailidou EP, Bitsori M, Tsagkaraki D, Mantzouranis E. Polyomyositis and myocarditis associated with acquired toxoplasmosis in an immunocompetent girl. BMC Musculoskelet Disord 2001;2:8.
65. Pena HFJ, Ferreira MN, Gennari SM, de Andrade HF Jr, Meireles LR, Galisteo AJ Jr. Toxoplasma gondii isolated from a Brazilian patient with rare pulmonary toxoplasmosis has a novel genotype and is closely related to Amazonian isolates. Parasitol Res. 2021 Mar;120(3):1109-1113. doi: 10.1007/s00436-020-07008-4. Epub 2021 Jan 9. PMID: 33420622.
66. Pustorino G, Ferlazzo E, Carpentieri MS, Cianci V, Gasparini S, Campello M, Milardi GL, Gangemi A, Aguglia U. Cerebral toxoplasmosis diagnosed by brain tissue PCR analysis in an immunocompetent patient. Neurol Clin Pract. 2017 Oct;7(5):436-438. doi: 10.1212/CPJ.0000000000000364. PMID: 29620083; PMCID: PMC5874466.
67. Rafiqul Islam et al.Toxoplasmosis Presenting As P.U.O.- A Case ReportBang. Med. J. 1989; 18 (1), 30-32; file:///C:/Users/dcontop/Downloads/ToxoplasmosisPresentingAsPUO-ACaseReport-1.p
68. Ramachandran R, Radhan P, Anand R, Subramanian I, Santosham R, Sai V. CNS toxoplasmosis in an immunocompetent individual. Radiol Case Rep. 2015 Dec 7;9(1):e00031. doi: 10.2484/rcr.v9i1.908. PMID: 27141248; PMCID: PMC4838758.
69. Roubille F, Roubille C, Lattuca B, Gervasoni R, Vernhet-Kovacsik H, Leclercq F. Recent Toxoplasmosis Infection With Acute Myopericarditis and Persistent Troponin Elevation in an Immunocompetent Patient. Cardiol Res. 2012 Aug;3(4):189-191. doi: 10.4021/cr200w. Epub 2012 Jul 20. PMID: 28348686; PMCID: PMC5358212.
70. Rösch D, Handrick W, Lietz R, Blatz R, König E. Erworbene Toxoplasmose mit zerebraler Beteiligung und nachfolgender Hörstörung [Acquired toxoplasmosis with cerebral involvement and subsequent hearing loss]. Klin Padiatr. 1998 May-Jun;210(3):125-7. German. doi: 10.1055/s-2008-1043863. PMID: 9629546.71. Rostoff, P.; Mroczek-Czernecka, D.; Piwowarska, W.; Gackowski, A.; Konduracka, E.; Trzos, M.; Pasowicz, M. Elevated CA-125 level in acute heart failure due to Toxoplasma gondii perimyocarditis. *Int. J. Cardiol.* **2008**, *130*, e114–e116.
72. Sano J, Saitoh H, Kobayashi Y, Ikeda M, Kodani E, Takayama M, Kishida H, Takano T, Yano A. [Toxoplasma pericarditis without immunosuppressant disorder detected by polymerase chain reaction of pericardial fluid: a case report]. J Cardiol. 2000 Jan;35(1):47-54. Japanese. PMID: 10654250.
73. Shimoni Z, Reichman N, Raz R, Flatau E. Pulmonary infiltrate in acute toxoplasmosis. Isr J Med Sci. 1989 Jan;25(1):46-7. PMID: 2925360.
74. Silva LA, Vieira RS, Serafini LN, Carlotti CG Jr, Figueiredo JF. Toxoplasmose do sistema nervoso central em paciente sem evideªncia de imunossupressao: relato de caso [Toxoplasmosis of the central nervous system in a patient without immunosupression: case report]. Rev Soc Bras Med Trop. 2001 Sep-Oct;34(5):487-90. Portuguese. doi: 10.1590/s0037-86822001000500014. PMID: 11600917.
75. Simanaityte D, Le Gouellec N, Ajana F, Baclet N, Poissy J, Laiskonis A, Mickiene A, Senneville E, Yazdanpanah Y, Legout L. Primo-infection toxoplasmique avec atteinte pulmonaire chez un immunocompétent [Primary pulmonary toxoplasmosis in an immunocompetent patient]. Med Mal Infect. 2010 Dec;40(12):713-5. French. doi: 10.1016/j.medmal.2010.03.013. Epub 2010 Apr 21. PMID: 20413237.
76. Singhal KK, Kumar K, Mathew JL, Mewara A, Vaidya PC, Sodhi KS, Singh M. Active toxoplasmosis presenting with polymyositis and pleural effusion in a child. J Paediatr Child Health. 2021 Dec;57(12):1995-1997. doi: 10.1111/jpc.15390. Epub 2021 Feb 11. PMID: 33571377.
77. Sobanski V, Ajzenberg D, Delhaes L, Bautin N, Just N. Severe toxoplasmosis in immunocompetent hosts: be aware of atypical strains. Am J Respir Crit Care Med. 2013 May 15;187(10):1143-5. doi: 10.1164/rccm.201209-1635LE. PMID: 23675724.
78. Sugane K, Takamoto M, Nakayama K, Tada T, Yuasa T, Kurokawa K. Diagnosis of Toxoplasma meningoencephalitis in a non-AIDS patient using PCR. J Infect. 2001 Feb;42(2):159-60. doi: 10.1053/jinf.2000.0768. PMID: 11531325.
79. Sánchez T, Soriano MJ, Almarza JL, CámaraM, Paricio P. DiagnÃ³stico de toxoplasmosis cerebral en un paciente inmunocompetente [Diagnosis of cerebral toxoplasmosis in an immunocompetent patient]. Enferm Infecc Microbiol Clin. 2000 Jan;18(1):46-8. Spanish. PMID: 10721565.
80. Undseth O, Gerlyng P, Goplen AK, Holter ES, von der Lippe E, Dunlop O. Primary toxoplasmosis with critical illness and multi-organ failure in an immunocompetent young man. Scand J Infect Dis. 2014 Jan;46(1):58-62. doi: 10.3109/00365548.2013.813065. Epub 2013 Aug 1. PMID: 23902584.
81. Vastava PB, Pradhan S, Jha S, Prasad KN, Kumar S, Gupta RK. MRI features of toxoplasma encephalitis in the immunocompetent host: a report of two cases. Neuroradiology. 2002 Oct;44(10):834-8. doi: 10.1007/s00234-002-0852-5. Epub 2002 Aug 24. PMID: 12389133.
82. Yang Y, Zuo W, Hu J, Esch GW, Zuo Y. Hemophagocytic syndrome as uncommon presentation of disseminated toxoplasmosis in an immunocompetent adult from Chinese Kunming. Am J Trop Med Hyg. 2013 Jun;88(6):1209-11. doi: 10.4269/ajtmh.12-0556. Epub 2013 Mar 18. PMID: 23509123; PMCID: PMC3752825.

## Appendix C

**Supplementary Tables and Figures for Cases Included in the Primary Analysis (1985–2022).**

**Table A1:** Countries of authors.

Authors’ Countries	N	Percent
Bangladesh	1	0.9
Belgium	1	0.9
Brazil	8	6.8
China	3	2.6
Colombia	12	10.3
Croatia	1	0.9
Denmark	1	0.9
France	30	25.6
French Guiana	13	11.1
Germany	1	0.9
Greece	2	1.7
India	10	8.6
Indonesia	1	0.9
Iran	1	0.9
Israel	1	0.9
Italy	5	4.3
Japan	3	2.6
Nepal	1	0.9
Norway	1	0.9
Oman	1	0.9
Pakistan	2	1.7
Peru	2	1.7
Poland	1	0.9
Spain	3	2.6
Taiwan	1	0.9
Turkey	1	0.9
UK	2	1.7
USA	8	6.8
Total	117	100

**Table A2:** Countries of infections with imputation as country of infection as the country of authors for those 61 cases not reporting the country of infection (cases with imputed country of infection are marked with *).

Countries of Infections (Imputed Countries for Those Not Reported Shown with *)	N	Percent
Bangladesh *	1	0.85
Belgium	1	0.85
Brazil	1	0.85
Brazil *	7	5.98
China	1	0.85
China *	2	1.71
Colombia Colombia *	8 4	6.80 3.42
Croatia	1	0.85
Denmark *	1	0.85
Ethiopia	1	0.85
France	2	1.71
France *	15	12.82
French Guiana	22	18.80
French Guiana *	1	0.85
Germany *	1	0.85
Greece*	2	1.71
India	2	1.71
India *	8	6.84
Indonesia *	1	0.85
Iran *	1	0.85
Israel *	1	0.85
Italy	1	0.85
Italy *	3	2.56
Ivory coast	1	0.85
Japan	1	0.85
Japan *	2	1.71
Nicaragua	2	1.71
Norway *	1	0.85
Oman	1	0.85
Pakistan *	2	1.71
Peru	1	0.85
Peru *	1	0.85
Poland	1	0.85
Qatar	1	0.85
Senegal	2	1.71
Spain	1	0.85
Spain *	2	1.71
Taiwan *	1	0.85
Turkey *	1	0.85
UK *	2	1.71
USA	3	2.56
USA *	2	1.71
Venezuela	1	0.85
Total	117	100.00

**Table A3:** Implicated *T. gondii* strains.

Reported genotyping	23/117 (20%)	
Atypical more virulent strain non-type II strains	22/23 (96%)	No type II virulent strain, African I strain, Africa or recombinant I/III Argentinian strain, Atypical strain GUY-2004-ABE, GUY-204-ANG, GUY-2004-TER, New and unknown zymodeme 12 highly virulent, identical with reference strain AF146527, virulent ROP18 allele, Zymodeme 6 virulent strain
Type II strain	1/23 (4%)	
Reported that no genotyping was done	15/117 (13%)	
Not reported genotyping information	79/117 (68%)	

Footnote: Genotyping data were very sparse. Documentation of atypical *T. gondii* strains by genotyping was performed only in 22 cases. There was also a case of spinal cord toxoplasmosis with Brown–Sequard syndrome in a 31-year-old immunocompetent man from France, where type II strain was isolated (Martinot 2020). Moreover, a case of a 41-year-old immunocompetent man from Brazil with acute pulmonary toxoplasmosis and respiratory failure was genotyped to be from a type III strain (Leal 2007).

**Table A4:** Pulmonary imaging findings.

Toxoplasmosis Lung Imaging Findings	N
**Pulmonary Toxoplasmosis cases** CXR: bilateral alveolar infiltrates.	**31** 1
CXR (sequential): mild bilateral interstitial infiltrate at the patient’s admission to the hospital (24 September 2005)// marked heterogeneous bilateral opacity 4 days later (28 September 2005), and regression of lung involvement at a follow-up visit (26 October 2005)	1
CXR: Bilateral pulmonary infiltrates (no other info)	1
CXR on admission showed patchy areas of consolidation in RUL and mid zones and left lower zone and multiple, ill-defined nodular opacities in both lung fields// F/Up CXR 1 month later showed resolution of lung changes///High Resolution Chest CT on admission showed an area of consolidation in the anterior segment of the RUL and multiple nodules with ground-glass opacity haloes around them// F/UP HRCT after 4 months shows resolution of consolidation and nodules with bronchiectatic changes in the segment previously affected by consolidation	1
CXR: Right lung infiltrates	1
CXR: bilateral interstitial infiltrates	1
CXR: bilateral mid and lower zone infiltrates in a reticular pattern//Chest CT: ground-glass opacities with bilateral minimal pleural effusion	1
CXR: cardiomegaly	1
CXR: cardiomegaly and bilateral pleural effusions	1
CXR: diffuse interstitial infiltrate. Chest CT: showed multiple nodular cavities and pleural effusion.	1
CXR: infiltrate in the right upper lobe	1
CXR: interstitial and alveolar infiltrate.	1
CXR: interstitial infiltrates in both lungs	1
CXR: noted left opacity. The thoraco-abdomino-pelvic CT scan showed an aspect of bilateral lower lobe pneumopathy with infiltrates, a small left pleural effusion.	1
CXR: pneumonia of the left lobe.	1
CXR: reticulonodular opacities in both lower zones	1
CXR: showed CHF// 2nd CXR showed bilateral interstitial and mild alveolar airspace changes in the right lower lobe and left retrocardiac region with small bilateral effusions	1
CXR: signs of pulmonary edema with cardiomegaly	1
CXR: small right pleural effusion	1
CXR: subtle diffuse interstitial infiltrates// PET/CT scan showed uptake in the peripheral and mediastinal lymphadenopathy.	1
Chest CT: Diffuse Ground Glass Opacities opacities and peribronchovascular and septal thickening, paratracheal lymphadenopathy	1
Chest CT Diffuse Ground Glass Opacities opacities, peribronchovascular and septal thickening, and nodules:∙ Chest CT with smooth septal and peribronchovascular thickening, ground-glass opacities (3/3),atelectasis (1/3), nodules (1.0–2.5 cm) (1/3 cases), paratracheal lymphadenopathy (1/3)and pleural effusion (2/3). ///may mimic pulmonary congestion, lymphangitis, atypical pneumonia and PJP pneumonia	1
Chest CT: Bilateral interstitial syndrome// Alveolar abnormalities: (Bilateral, R > L)	1
Chest CT: diffuse Ground glass opacities, peribronchial and septal thickening, atelectasis, small bilateral effusion/may mimic pulmonary congestion, lymphangitis, atypical pneumonia and PJP pneumonia	1
Chest CT: ground glass pattern with left lower lobe pulmonary atelectasis	1
Chest CT: interstitial syndrome and mediastinal LADP	1
Chest CT: moderate bilateral interstitial infiltrates and bilateral pleural and pericardial effusions without mediastinal lymphadenopathy.	1
Chest imaging (not specified): Alveolar abnormalities: Bilateral (inferior) Interstitial abnormalities: Bilateral	1
Chest imaging (not specified): Alveolar abnormalities: Bilateral Interstitial abnormalities: Mild Pleural effusion//Cardiomegaly	1
Chest imaging (not specified): Alveolar abnormalities: Bilateral Interstitial abnormalities: Mild, bilateral	1
Chest imaging (not specified): Alveolar abnormalities: White Lung	1
Chest imaging (not specified): Bilateral alveolar interstitial syndrome Alveolar abnormalities: Bilateral Interstitial abnormalities: Bilateral Pleural effusion: L	1
Chest imaging (not specified): Bilateral alveolar interstitial syndrome//Alveolar abnormalities: Bilateral, basal//Pleural effusion: (Bilateral, L > R)//Other abnormalities: Cardiomegaly	1
Chest imaging (not specified): Bilateral alveolar interstitial syndrome; left Pleural effusion//Alveolar abnormalities: Basal, R > L//Interstitial abnormalities: Mild, bilateral	1
Chest imaging (not specified): Bilateral interstitial syndrome// Alveolar abnormalities (bilateral, R > L)//Interstitial abnormalities (bilateral)//Pleural effusion (R)// Other abnormalities (Cardiomegaly)	1
Chest imaging (not specified): Bilateral interstitial syndrome// Alveolar abnormalities: Bilateral (R > L)//Interstitial abnormalities: Bilateral	1
Chest imaging (not specified):Bilateral alveolar interstitial syndrome//Alveolar abnormalities: Bilateral Interstitial abnormalities: Bilateral (L > R)//Pleural effusion: L	1
Chest imaging: Interstitial pneumonia (not specified further)	1
Chest/Abdomen CT showed disseminated glandular hypertrophy in the neck, thorax, and abdomen and slight pericardial effusion, bilateral pleural fluid, and bilateral pulmonary interstitial infiltrates.	1
Imaging showed mediastinal (and abdominal LADP)	1
Thoraco-abdomino-pelvic tomodensitometry (TAP TDM): Micronodular infiltrates in both lung fields// PET Scan: Diffuse pulmonary infiltrates intensely metabolic	1
“US suggested a doubtful abscess in the left paraspinal and oblique muscles of abdomen, along with mild bilateral pleural effusion”. //Contrast-enhanced CT of chest and abdomen showed bilateral pleural effusion with bulky heterogeneous left posterior paraspinal muscle, but no signs of an abscess.///CXR showed bilateral costophrenic angle blunting, but the lungs and bones were normal.	1
Not reported	15
Not applicable (non-pulmonary involvement)	64
Total	117

**Table A5:** CNS imaging findings.

CNS Imaging Findings	N
**CNS toxoplasmosis cases** Brain MRI T1 images show multiple hypointense lesions involving the right frontal and left temporoparietal lobes///T2 and FLAIR images of the brain show multiple hyperintense lesions involving the right frontal and the left temporoparietal lobes/////Pre- and postcontrast brain CT images show multiple intensely enhancing lesions (star) involving the bilateral cerebral hemispheres////T2W and DWI images show new hyperintense lesions with restricted diffusion in the left thalamus and the globus pallidus///ADC image shows low signal in the left thalamus suggestive of true restriction///MR spectroscopy of the lesion involving the right frontal lobe shows elevated lipid lactate peak.	41 1
Brain CT (non-contrast): Cerebral edema, flattening of the grooves and disappearance of contrast between white matter and cortex; Brain MRI: confirmed brain edema and picture of hemorrhagic cortical infarct. (brain biopsy was not diagnostic)	1
Brain CT and MRI: normal.(patient with hemidystonia)	1
Brain CT and brain MRI were normal (in this case of NMDA encephalitis associated with acute Toxoplasmosis in this 9 yr old immunocompetent girl, who presented with personality and behavioral changes, fever, recurring seizures, headache and vomiting and whose symptoms rapidly improved (except for residual agitation) and completely resolved 2 months after a 10 ds course of anti-*Toxoplasma* treatment with azithromycin and without the need for immunotherapy///A repeat brain MRI was normal. (The EEG demonstrated 14Hz sporadic positive spikes without diffuse slow activity, and epileptiform discharges)	1
Brain CT showing multiple calcifications involving the basal ganglia and semioval centers//Brain MRI T2 weighted images showing multiple cystic lesions surrounded by brain edema, non-enhancing after contrast (in a 30 yr old immunocompetent man with a 5 yr hx of monthly frontal headache, with worsening intensity and frequency over the last month, with associated episodes of sudden falls without loss of consciousness, followed by unjustified crying, after eating poorly cooked pork meat; with EEG showing mild slowing over left temporal region// with no follow up for 20 mo, but due to daily severe headaches and secondary generalized seizures patient was readmitted)///with repeat Brain CT and MRI showing marked size increase of cystic lesions with ring enhancement and diffuse perilesional edema with mildline shift (S/P emergency surgical removal of 2 large brain cysts, histopathologically showing several bradyzoites and positive T.gondii DNA PCR//with initiation of anti- *Toxoplasma* treatment with pyrimethamine/sulfadiazine + dexamethasone and at f/up 20 mo later with resolution of Headaches and persistence of sporadic partial motor seizures)//With f/up brain MRI 20 mo after treatment showing mild decease in size of cystic lesions with reduction of perilesional edema and contrast enhancement	1
Brain CT w contrast: multiple ring enhancing lesions; moderate hydrocephalus and meningeal enhancement (first diagnosis was TB meningoencephalitis although CSF AFB stain and Cultures were negative; patient was started on anti-TB therapy + steroids--> fever, HA and irritability persisted over the next 10 ds)// After 10 ds of anti-TB treatment +Steroids Brain MRI showed: T1 isointense to hyperintense exudate in the suprasellar cistern; T2 images showed dilation of lateral, 3d and 4th ventricles w periventricular hyperintensity and multiple widely scattered ring lesions with hypointense center and hyperintense rim peripherally in b/l cerebral hemispheres including thalamus and basal ganglia (also in pons, middle cerebellar peduncles and b/l cerebellar hemispheres) associated with perifocal edema (initial diagnosis TB meningitis w multiple tuberculomas)//////After 6 weeks of anti-*Toxoplasma* treatment: all lesions showed regression in size and some lesions became calcified	1
Brain CT: disclosed an isodensity lesion encapsulated by surrounding edema//Brain MRI revealed a space-occupying lesion with isointense in T1 and hypointensity in T2-weighted imaging and adjacent cerebral edema.// Mushroom-shaped ring enhancing lesions with irregular-enhanced rim were observed in MRI after contrast injection; with perilesional brain edema in the left temporal lobe.///Postoperative brain MRI showed total excision of the lesion with no recurrence 2.5 years after surgery///(Histological examination demonstrated the presence of protozoan parasite in a pseudocyst structure and a capsule consisted of lymphocytes, plasmacytes, and macrophages among the inflammatory infiltrate tissues///A typical ring like pseudocyst structure was surrounded by different inflammatory cells)	1
Brain CT: hypodense lesions in temporo-occipital region, w vasogenic edema and adjacent hemorrhagic focus	1
Brain CT: low-grade edema in the area of the posterior skull but without lateralized displacement of the 4th ventricle.	1
Brain CT: showing hypodense lesion in left frontoparietal region//Brain MRI T1 images showing a hypodense lesion in the left parietal area.//Brain MRI T1 contrast and T2weighted image showed lesion that is contrast enhancing, while on T2 it is hyperintense.	1
Brain CT: ventriculitis in the 3d and 4th ventricles, and temporal horns of both lateral ventricles w ring enhancement.	1
Brain CT: w numerous calcifications and Hypodense areas in the frontal and parietal lobes//Brain MRI with high signal in T2 in periventricular white matter, internal capsule, and “centres semi-ovales”	1
Brain FLAIR MRI on 13 April showed a high signal lesion in the left parietal region///Brain MRI on 29 May: Axial FLAIR shows enlargement of the original lesion in addition to a new contralateral lesion///Brain MRI on 12 June: Axial FLAIR shows lesions in the bilateral temporal and frontal regions///Brain MRI on 12 June: On T2-weighted imaging, a three-layered structure with a low signal, high signal and low signal (T2 target sign) is observed from the center of the elliptic lesion in the left parietal region//Brain MRI on 4 September: Axial FLAIR shows gradual improvement along with the patient’s improvement on a consciousness level, the disappearance of involuntary movement, and lesion reduction and ultimate disappearance on head MRI. The center region has a high signal on apparent diffusion coefficient (ADC) mapping.	1
Brain MRI T1 weighted images revealed a hypointense pontine lesion with extension into the midbrain. The lesion was heterogeneously enhancing with contrast with vague ring enhancement dorsally////(He underwent microsurgical decompression and biopsy of the lesion)	1
Brain MRI T2 images showed Hyperintense lesions in both thalami and putamina and head of caudate nucleus without diffusion restriction on DWI sequences (T2 shine through) ///(69 yr old immunocompetent HIV negative male with a 2 mo hx of rapidly progressive cognitive decline with poor speech, dyslexia, dysgraphia, constructional apraxia, verbal and visual memory deficits, positive Babinski and left side and gait apraxia//CSF showed elevated protein (1.08 g/L) without pleocytosis and elevated CRP//Clinical curse complicated by gram negative sepsis and patient died, with toxoplasmic encephalitis revealed at the autopsy///Brain autopsy showed confluent small and soft partially hemorrhagic lesions in basal ganglia with few cysts containing *T.gondii* bradyzoites	1
Brain MRI T2 weighted images showing ill-defined hyperintense lesion in Right cerebellar hemisphere with perilesional edema, extending along the right foramen of Luschka///Post Contrast T1 images showing heterogeneous post-contrast enhancements with peripheral enhancing ring//(23 yr old immunocompetent male residing in Oman with a 2 wks Hx of weakness, incoordination of R upper and lower extremities, 5 ds Hx of throbbing headache, and visual blurring, ///Underwent surgical excision due to concern for malignant space occupying lesions///histopathology showed necrosis, hemorrhage, perivascular infiltrates, *T.gondii* cysts and evidence of cerebritis and encephalitis///treated with clindamycin + Azithromycin due to G6PD deficiency//post surgery neurologic status deteriorated and Brain MRI showing increase in perilesional edema and inflammation involving the brain stem (Steroid doses were increased//Patient clinically deteriorated despite anti- *Toxoplasma* treatment and died the 10th postsurgical day due to spreading *Toxoplasma* cerebritis and encephalitis)	1
Brain MRI T2-weighted sequences: hyperintensity in the brainstem affecting both cerebral peduncles, predominantly in the right ///Normal head CT///Repeat MRI at 3 months: Showing considerable reduction in the size of the lesion.	1
Brain MRI did not show any abscess lesions	1
Brain MRI on admission showed a lesion in the right globus pallidus and adjacent genu of the right internal capsule, which is a hypointense on T1-weighted images and hyperintense on T2-weighted images. //DWI and ADC (apparent diffusion coefficient) of the brain showed the lesion to have a peripheral rim of restricted diffusion, with a central area of facilitated diffusion///Fluid attenuated inversion recovery magnetic resonance images (FLAIR-MRI) on admission showed the heterogeneously hyperintense lesion in the right basal ganglia with repeat images at 5 mo f/up showing significant reduction in size///Postcontrast coronal T1- weighted images on admission show the same lesion enhancing peripherally, which on follow-up after 5 months shows significantly reduced enhancement. ///Postcontrast coronal T1-weighted images on admission showing another enhancing nodular lesion in the right superior frontal gyrus; that is not seen on follow-up	1
Brain MRI revealed hyperintense lesion in the right basal ganglia, periventricular grey matter, left frontal and parietal midbrain and pons suggestive of glioblastoma multiforme.//A repeat CT of the hear showed a significant decrease in the size of the lesion and some edema around the lesion (following treatment).	1
Brain MRI showed no edema, vascular or focal lesions (CSF analysis revealed pleocytosis (35 cells/mm3)	1
Brain MRI showing heterogeneous ring-enhancing lesion in the brainstem.///(Biopsy shows large zones of necrosis; fresh hemorrhage and dense inflammation; Thin walled veins show inflammation and necrosis of wall; Several tachyzoite forms of *Toxoplasma gondii* seen in the lesion on immunohistochemistry)	1
Brain MRI was initially normal///The second cerebral MRI showed meningeal enhancement, but no intracerebral pathology	1
Brain MRI with multiple high signal lesions bilateral in T2 (with low signal in T1). Most lesions showed “linear radiating” and nodular contrast enhancement. Also enhancing lesions in L temporal lobes appeared normal in non-contrast images)//At 6 mo f/up brain MRI: minimal residual lesion in the left parietal region on T2, which does not show contrast enhancement//	1
Brain MRI: T2 weighted image showed an insult at the left thalamus to internal capsule (the lesion had resolved at the 3 mo Brain MRI f/up)	1
Brain MRI: images suggestive of ADEM (multiple hyperintense lesions in T2 images; FLAIR pseudonodules with vascular distribution	1
Brain MRI: large hemorrhagic lesion in the R parietal region with perifocal edema and mass effect (mixed signal R periventricular lesion surrounded by edema which gives high signal in T1 and is consistent with hemorrhage); multiple lesions with high signal in T2 in L cerebral and cerebellar hemispheres; lesions with low signal or isointense in T1//Contrast enhanced images showed multiple cortical and subcortical enhancing lesions and linear enhancement in both cerebral and cerebellar hemispheres//Brain MRI: showed narrowing of medium size vessels suggestive of vasculitis//After 6 mo: small residual lesions in T2 with no contrast enhancement	1
Brain MRI: multiple ring enhancing masses in the coronal radiation, brainstem and cerebellum; EEG: normal	1
Brain MRI: normal	1
Brain MRI: normal //(15 yr old immunocompetent boy, with LADP and recurrent seizures, after vacation to Ethiopia, with EEG showing sporadic single spike or sharp-wave paroxysms//with *T.gondii* serology consistent with acute primary infection//with normalization of the EEG after 4 weeks of anti- *Toxoplasma* treatment with P/S/FA//with recurrence of seizures 5 months later, with reappearance of fatigue and cervical LADP)//Negative repeat brain MRI (excluding any abscess) (with elevated CSF protein level (250 mg/dl; with the rest of the CSF analysis normal//with negative CSF *Toxoplasma* PCR at which time a diagnosis of epilepsy was made and seizures were controlled with valproic acid (this case represented the first reported case of seizures during acute primary toxoplasmosis in immunocompetent patient)	1
Brain MRI: normal (in the context of cerebellar syndrome and rhomboenecephalitis)	1
Brain MRI: showed contrast enhanced T1-weighted images: peripheral enhancing lesion with central diffusion restriction in the left thalamus consistent with microabscess//Contrast enhanced T1 weighted image: Perivascular enhancing lesion with restricted diffusion with vasogenic edema and leptomeningeal enhancement (in Brain MRI) //Spectroscopy showed an elevated lipid lactate peak. //Perfusion MRI images demonstrated increased microvascular permeability	1
Brain MRI: small areas (in the occipitoparietal brain cortex bilaterally and in the right hemisphere of cerebellum) of low density probably related to *Toxoplasma* infection	1
Cerebro-medullar MRI: Leptomeningitis encephalic stage Absence of lesion on medullar floor	1
FLAIR MRI (Fluid-attenuated, inversion-recovery axial magnetic resonance imaging) disclosed high-intensity lesions in the periventricular white matter, corona radiata, centrum semiovale, and left parietal lobe///There was no mass effect and no contrast enhancement. All the lesions were hyperintense on both diffusion-weighted imaging and apparent diffusion coefficient images///MRI of the spinal cord revealed diffuse abnormal signal intensity between the cervical C4 to the thoracic T6 level, which showed no enhancement	1
Head CT: hydrocephalus and fronto-parietal periventricular hypodensities are observed. (in this 72 yr old immunocompetent patient from Colombia with progressive neurologic deterioration and rapid evolution of multiorgan failure and death)	1
Head CT: multiple nodular calcifications, parenchymatous, distributed along the convexity, paracortical regions and brain base. (EEG: consistent with diffuse atrophy (brain bioelectrical activity with a slow dominant frequency)	1
Head CT: no abnormalities (case with encephalitis)	1
Head CT: showed multifocal white matter lesions//Brain MRI revealed extensive bilateral cortical and subcortical concentric ring-enhancing white matter lesions throughout both cerebral hemispheres. The lesions were more prominent in left parietal and frontal lobes//Treatment with steroids for presumed multiple sclerosis caused worsening of symptoms and increase in size of the brain MRI lesions//Brain biopsy confirmed *T.gondii* infections by histopathology and PCR and treatment with Pyrimethamine/Sulfadiazine decreased the size of the brain lesions dramatically///The initial differential diagnosis was brain abscesses vs demyelinating disease (multiple sclerosis). MRI of the spine showed no abnormalities. (CSF analysis showed elevated protein levels but no cells. Myelin basic protein and oligoclonal bands in the CSF were negative. Results of visual evoked potential studies were normal bilaterally. No vegetations were noted on the transthoracic echocardiogram. Ophthalmologic examination showed no evidence of chorioretinitis. ///A stereotactic brain biopsy was done, and CNS toxoplasmosis was reported after biopsy and polymerase chain reaction analysis)	1
MRI brain showed multiple ring enhancing lesions in white and grey matter involving corpus callosum, subcortical areas and periventricular region in frontal, parietal and temporal lobes. The lesions were surrounded by vasogenic edema appreciated on coronal FLAIR image. AFB smear and MTB DNA were negative.	1
Not applicable for main toxoplasmosis manifestation (patient during the first hospitalizations mental status changes led to a Brain MRI that showed possible tiny cerebellar infarcts, while spine MRI was unremarkable AND spinal tap with CSF with mild CSF protein elevation without CSF pleocytosis)	1
Spinal MRI showed a 12-mm intramedullary C3-C4 cervical tumor on the left that had ring enhancement after a gadolinium injection. Brain Imaging done at the same time was normal. ///Three years after the infection, the patient remained in good health. A follow-up MRI showed a sequellar intramedullary image without contrast enhancement after the gadolinium injection ///(case of an immunocompetent 31 yr old with spinal cord toxoplasmosis; who had complete regression of all neurologic deficits after few weeks of pyrimethamine/sulfadiazine, and who after secondary prophylaxis x1 year had f/up MRI showing sequellar intramedullary image without contrast enhancement)	1
Not reported	18
Not applicable	58
Total	117

Abbreviations: CSF: Cerebrospinal fluid, EEG: Electroencephalogram, HIV: Human immunodeficiency, LADP: Lymphadenopathy, mo: Month, MRI: Magnetic resonance imaging, MTB: Mycobacterium tuberculosis, PCR: Polymerase chain reaction, P/S/FA: Pyrimethamine/sulfadiazine/folinic acid, yr: Year.

**Table A6:** Cardiac imaging findings.

Cardiac Cases (ECHO, Cardiac MRI, EKG)	N
Cardiac toxoplasmosis Patient died from Cardiac arrhythmia (Ventricular Fibrillation)//(Patient had CNS toxoplasmosis)	34 1
Cardiac MRI showed subepicardic enhancement during late phase of gadolinium administration in the absence of subendocardial ischemic pattern, compatible with myopericarditis. ///Cardiac MRI also revealed mild pericardial effusion with normal ventricular function.	1
Cardiac MRI: LGE (late gadolinium enhancement) demonstrated a lumpy subepicardial diffuse enhancement. Mild pericardial effusion was confirmed. These findings were concordant with the diagnosis of myocarditis//Normal ECG (with mild ST-segment elevation)//Normal CXR	1
Cardiac MRI: myocardial necrosis in several segments; but otherwise normal LV volume and LV systolic function, global mormokinetic///EKG small q wave in II, III, aVF, and V4-V6 and t wave flattening in II, III, aVF.	1
Chest CT revealed a moderate bilateral interstitial infiltrates and bilateral pleural and pericardial effusions without mediastinal lymphadenopathy.	1
Chest imaging (not specified): Alveolar abnormalities: Bilateral Interstitial abnormalities: Mild Pleural effusion//Cardiomegaly///ECHO: Pericarditis, low EF, Pulmonary Hypertension	1
Chest imaging (not specified): Bilateral alveolar interstitial syndrome//Alveolar abnormalities: Bilateral basal//Pleural effusion: (Bilateral, L > R)//Other abnormalities: Cardiomegaly////ECHO: Pericarditis, Hypokinesia, Low EF 26%	1
Chest imaging (not specified): Bilateral interstitial syndrome//Alveolar abnormalities (bilateral, R > L)//Interstitial abnormalities (bilateral)//Pleural effusion (R)//Other abnormalities (Cardiomegaly)//ECHO: pericarditis	1
Chest/abdomen CT scan showed disseminated glandular hypertrophy in the neck, thorax, and abdomen and slight pericardial effusion, bilateral pleural fluid, and bilateral pulmonary interstitial infiltrates.///ECHO was normal, except an insignificant pericardial effusion///Troponin T was continuously elevated///Telemetry detected multiple periods with atrial tachycardia and an EKG showed changing P waves	1
ECG showed complete atrioventricular (AV) block, with a narrow QRS at a rate of 32 bpm and an atrial rate of 66 bpm///Cardiac enzymes and ECHO were normal//An ergometric test showed no increase of heart rate on physical activity///Several EKGs and 24 h recordings showed constant complete AV block with a junctional rate of 32 bpm.	1
ECG: subtle lateral ST segment elevation in leads I, II, V5, and V6.///Cardiac MRI: consistent with an acute phase of biventricular myocarditis and heart failure (LVEF 45% and RVEF 41%), despite normal ECHO (cardiac MRI was markedly abnormal consistent with an acute phase of biventricular myocarditis with associated edema and late gadolinium enhancement (LGE) of the mid-myocardium; with transmural apex involvement; also w patchy enhancement of the myocardium as well as the right ventricle) //with elevated Troponin: 8.51 ng/mL with peak of 12.8	1
ECHO cardiac abnormalities (mild dilated myocardiopathy with pericarditis)	2
ECHO disclosed severe widespread left-ventricular systolic dysfunction with LVEF 33%, and a large pericardial effusion without echocardiographic signs of significant elevation of pericardial pressure//Chest CT confirmed the presence of pericardial and pleural effusions///EKG revealed sinus rhythm, low voltage in limb leads, and previously present left bundle branch block///CXR showed bilateral pleural effusions and an enlarged cardiac silhouette///Cardiac catheterization showed no significant coronary artery lesions	1
ECHO displayed moderate pericardial effusion, hyperechoic pericardium slightly detached in the back side and hypokinetic apex with estimated ejection fraction of 50% ///(The patient was discharged on day 12 because global cardiac function recovered and pericardium effusion disappeared on ECHO examination)	1
ECHO revealed an altered LVEF (35%), global hypokinesia///EKG showed sinus tachycardia	1
ECHO: systolic left ventricular dysfunction with an akinetic basal and median septum and hypokinesia of the other ventricular walls. The shortening fraction was 10% (normal 30–35%) and the ejection fraction 35% (normal 60%). There was no pulmonary hypertension, but some functional mitral valve insufficiency and grossly dilated supra-hepatic veins///ECG: sinus rhythm and a heart rate of 120 with no S-T segment abnormalities	1
ECHO: dilation of Left ventricle w poor contractility suggestive of myocardial involvement) (EMG consistent with subacute inflammatory myopathy)	1
ECHO: global hypokinesia with a shortening fraction of approximately 17% (normal 30–35%) and an ejection fraction of 36% (normal 60%) but no pericardial effusion///ECG: sinus tachycardia with diffuse S-T segment depression but no classical saddle shape	1
ECHO: low ejection fraction estimated at 30%, evidence of moderate mitral regurgitation and global hypokinesis///Right and left cardiac catheterizations: normal coronary arteries, elevated right heart pressures, pulmonary capillary wedge pressure 30 mm Hg///Endomyocardial biopsy results consistent with myocarditis of unknown etiology.///ECHO (after treatment with prednisone): normal size and function of the left ventricle with mild mitral regurgitation, mild tricuspid regurgitation, and an estimated ejection fraction of 60%	1
ECHO: massive pericardial effusion///Gallium scan: increased uptake around the heart and mediastinal regions	1
ECHO: showed revealed global hypokinesia with ejection fraction of 45% on initial presentation but dropping to 10% by hospital Day 2. Cardiac Index of 1.0. in cardiac catheterization//At presentation he was found to be in cardiogenic shock, with multiple organ failure, and was admitted to the intensive care unit. EKG: showed sinus rhythm with no changes concerning for ischemia. (Cardiac biopsy revealed fragments of myocardium with interstitial edema, patchy myocytolysis, and focal myocyte vacuolization suggestive of myocyte injury without evidence of active myocarditis. ////Clinical findings of myocarditis had resolved by the time the toxoplasmosis diagnosis was made and anti- *Toxoplasma* treatment was initiated)	1
ECHO: pericarditis	1
EKG on admission showed an incomplete right bundle branch block///ECHO and coronary angiography showed no abnormality//Cardiac MRI showed areas of myocardial edema.	1
EKG with ST elevation suggestive of acute myocardial infarction, ECHO: normal; Coronary angiography: no stenosis; Thalium scan with non-homogeneous uptake laterally in early phase with normalization in late phase-->(started spiramycin)--> F/up ECHO 4 months later normal	1
EKG/ECHO: normal////Elevated troponin (31.3 ng/L). (During his hospitalization, the patient’s clinical condition improved and levels of troponin and CRP normalized 3 days after the appearance of the cardiac symptoms and was discharged 4 days after admission with an appointment for cardiac magnetic resonance imaging. This revealed probable lateral myocarditis.)	1
EKG: concave ST elevation, ECHO: large pericardial effusion, without tamponade or evidence of myocarditis//	1
Echo: normal (day of first admission)///ECG: indicative of pericarditis (several days after first admission)///A few days later, CXR showed an enlarged heart and ECG disclosed general ST segment elevation and low voltage.	1
Echo: tricuspid and mitral insufficiency, pericardial fluid and decreased myocardial contraction	1
Heart Rhythm abnormalities/ECHO normal	1
Myocarditis-details not reported	3
On admission: irregular apical HR 70 b/min and BP 70/40 mm Hg initially, later unrecordable////ECG: (AV) dissociation, with a nodal rate of 70 b/min and an atrial rate of 40 b/min///2nd ECG: widespread T-wave inversions///3rd ECG: widespread T-wave inversions///Echo (36 hrs later): impaired LVF///Infusion of dobutamine and then noradrenaline only raised the systolic BP to 60 mm Hg///An atrial pacing wire was inserted. Wenckebach AV block occurred at an atrial pacing rate of 80 beats/minute.///Dual chamber sequential pacing at 1 10 beats/minute restored BP to 100/50 mm Hg with a brisk diuresis.	1
Tachyarrhythmia	1
Troponin I was mildly elevated at 0.26 ng/mL (0.00–0.05), but ECG was within normal limits.	1
Troponin I was mildly elevated at 0.1 ng/mL (0.00–0.05)///normal ECG	1
Not reported	7
Not applicable	76
Total	117

Abbreviations: BP: Blood pressure, CRP: c-reactive protein, Chest CT: Chest computed tomography, CXR: Chest radiograph, ECHO: Echocardiogram, ECG/ EKG: Electrocardiogram, EMG: Electromyogram, LVEF: Left ventricular ejection fraction, RVEF: Right ventricular ejection fraction.

**Table A7:** Anti- *Toxoplasma* treatments.

Treatments	N	Percent
Azithromycin	2	1.71
Azithromycin + Clindamycin	1	0.85
Clindamycin	1	0.85
Erythromycin and then Pyrimethamine + Sulfadiazine	1	0.85
Erythromycin initially, then after diagnosis Pyrimethamine + Sulfadiazine, initially, then spiramycin	1	0.85
Fansidar + Spiramycin (initially), then Pyrimethamine + Sulfadiazine, then fansidar maintenance	1	0.85
No anti-*Toxoplasma* treatment	14	11.97
Not reported	5	4.27
Not reported (TMP/SMX given later for the ocular findings (intravitreal clindamycin + dexamethasone (DXM]))	2	1.71
Pyrimethamin + Sulfadiazine +DXM (initially), then Pyrimethamine + Clindamycin	1	0.85
Pyrimethamine + Clindamycin	3	2.56
Pyrimethamine + Spiramycin	1	0.85
Pyrimethamine + clindamycin	1	0.85
Pyrimethamine +Sulfadiazine + Spiramycin	1	0.85
Pyrimethamine + Sulfadiazine	52	44.44
Pyrimethamine + Sulfadiazine + clindamycin	1	0.85
Pyrimethamine + Sulfadiazine + Spiramycin	1	0.85
Spiramycin	4	3.42
Spiramycin (initially) and then Pyrimethamine + Sulfadiazine	1	0.85
Spiramycin (initially), and then Pyrimethamine + Sulfadiazine	1	0.85
Spiramycin (initially), then Pyrimethamine + Sulfadiazine	1	0.85
TMP/SMX	11	9.40
TMP/SMX (initially) and then Pyrimethamine	1	0.85
TMP/SMX (initially), then Azithromycin + Clindamycin	1	0.85
TMP/SMX (initially), then Pyrimethamine + Sulfadiazine	1	0.85
TMP/SMX (initially), then Pyrimethamine + Sulfadiazine	1	0.85
TMP/SMX (initially), then clindamycin	1	0.85
Unspecified treatment	1	0.85
Unspecified anti-*Toxoplasma* treatment	4	3.42
Total	117	100.00

**Table A8:** Clinical Vignettes (for the 117 cases published between 1985 and 2022).

Author	Vignettes of Severe Toxoplasmosis Cases
Nelwan 2022	Acute toxoplasmic myocarditis in a 28-yr-old male who presented w fever x8 wks and chest pain x1 wk PTA; cardiac MRI showing Late Gadolinium Enhancement (LGE) areas consistent with myocardial necrosis but w normal LV systolic function; elevated cardiac enzymes (hs-Troponin, CK-MB and proBNP), transaminitis, lymphopenia, hyponatremia//Patient responded initially to steroid treatment but relapsed again within 2 wks from the first hospital admission//This led to the diagnosis of Acute Toxoplasmosis (Acute Primary Infection) and initiation of anti- *Toxoplasma* treatment with Pyrimethamine + Clindamycin x 8 weeks//clinically improved after 4 wks of anti- *Toxoplasma* treatment, also w normalization of laboratory values//
Bhattarai 2021	B/L interstitial pneumonia w respiratory distress in a 35-yr-old male with associated LADP and transaminitis (w fever, cough, respiratory distress, hypoxia requiring ICU admission; w subsequently development of b/l inguinal not tender LADP, hepatomegaly and lab values indicating transaminitis; hyperbilirubinemia//W Chest imaging findings suggestive of atypical pneumonia w CXR w b/l pulmonary infiltrates and Chest CT indicating b/l GGO with minimal b/l pleural effusions//With clinical and radiographic response to anti-*Toxoplasma* treatment (radiographic resolution of CXR findings at 4 wks f/up)
Filipowics 2021	Cardiogenic shock 2/2 acute myocarditis w multiple organ failure in a 59-yr-old female, with need for ICU admission, w CHF with ECHO showing global hypokinesia w EF 45% initially dropping to 10% by HD2; w cardiac index 1.0 in cardiac catheterization; w elevated cardiac biomarkers, transaminitis, and leukocytosis//Initially managed w steroids for presumed infectious myocarditis w CHF//Cardiogenic shock with multiorgan failure and ICU admission, ECHO with global hypokinesia (EF 10%), elevated cardiac enzymes. The toxoplasmosis diagnosis was made >1 mo after onset of cardiac symptoms, when he developed ocular inflammation; at which time cardiac findings had resolved without specific anti-*Toxoplasma* treatment//Diagnosed with Toxoplasmosis based on acute serology at the time of ocular inflammation evaluation, despite prior negative myocardial biopsy histopathology for *Toxoplasma* and negative aqueous and vitreous fluid *Toxoplasma* PCR//Started anti- *Toxoplasma* treatment and steroids at the time of ocular inflammation presentation with resolution of chorioretinitis//Treated with TMP/SMX for 6 weeks and got daily suppressive treatment for 1 year) (Additional info: Underwent Myocardial biopsy. Diagnosed as viral myocarditis. //CSF evaluation due to initially associated altered mental status showed high protein (CSF protein 61)//D/C home after 1 mo of first hospitalization with several nosocomial infections//DOI 1 mo: developed floaters, decreased visual acuity and severe vitritis and b/l endophthalmitis. Failed antifungal treatment. Improved with vitrectomy in Left eye. Vitreous aspirate *Toxoplasma* PCR negative. //*Toxoplasma* serology suggestive of API with positive *Toxoplasma* IgG/IgM and avidity testing//Review of previous myocardial biopsy was negative for *Toxoplasma* cysts//Aqueous humor fluid was *Toxoplasma* PCR negative//Had vitrectomy also in R eye with improved vision, BUT after surgery developed chorioretinitis in Left eye consistent with ocular toxoplasmosis. //Started anti- *Toxoplasma* treatment and steroids with resolution of chorioretinitis//treated with TMP/SMX for 6 weeks and got daily suppressive treatment for 1 year)/////(also nosocomial pseudomonas pneumonia, clostridium difficile colitis, AKI requiring hemodialysis)//Required prolonged hospitalization x 1 mo (w several nosocomial infections) and was d/c in a long term care facility; where he developed decreased visual acuity and floaters b/l 2 mo after onset of cardiac symptoms w new onset of b/l panuveitis/chorioritenitis; at which time the diagnosis of Toxoplasmosis was made based on positive *Toxoplasma* serology for API (with elevated *Toxoplasma* IgG and IgM titers; avidity was done but results not given)/////The study did NOT clearly report when the cardiac symptoms had resolved; but probably the cardiogenic shock had resolved before the initiation of anti- *Toxoplasma* treatment //(Despite negative reexamination of prior cardiac Bx, NOT showing *T.gondii* cysts and despite negative aqueous and vitreous fluid *T.gondii* PCR)//with complete resolution of chorioretinitis within 1 wk of anti-*Toxoplasma* treatment with TMP/SMX+ Steroids, w no recurrence of ocular or systemic symptoms
Lima 2021	Encephalitis with Right hemiparesis (but without altered level of consciousness) in a 60-yr-old male presenting with a 2 ds hx of HA and R sided weakness (R hemiparesis); with CSF mild pleocytosis (CSF WBC 15 cells/mm3), predominantly lymphomononuclear and brain MRI showing a peripheral enhancing lesion with central diffusion restriction and perivascular enhancing lesion with restricted diffusion with vasogenic edema and leptomeningeal enhancement in the white matter//w brain biopsy, revealing diffuse encephalitis with necrotic brain parenchyma and predominantly acute inflammation, AND *T.gondii* cysts with bradyzoites seen in the brain parenchyma//Anti- *Toxoplasma* treatment initiated with P/S x 6 weeks with complete clinical improvement of neurologic symptoms and MRI findings with regression of the brain lesion with no pathologic contrast enhancement
Mustafa 2021	Acute myocarditis with heart failure in a 2-yr-old male, presenting with recurrent acute chest pain over 1 week period (w the first 2 episodes having self-resolved), w EKG with subtle lateral ST segment elevation//with elevation of cardiac enzymes (peak troponin 12.8 ng/mL; unl < 0.3), mild transaminitis (AST 108 and ALT 91)//with Cardiac MRI: consistent with an acute phase of biventricular myocarditis and heart failure (LVEF 45% and RVEF 41%) (despite normal ECHO), with associated edema and late gadolinium enhancement (LGE) //with elevated Troponin: 8.51 ng/mL with peak of 12.8//With resolution of cardiac symptoms after 18 ds of anti- *Toxoplasma* treatment with Pyrimethamine + Sulfadiazine
	
Sinhal 2021	Polymyositis and b/l pleural effusion; in a 12-yr-old-male, who presented w fever, cough, HA, malaise and weight loss x 4 months PTA, with B/L pleural effusion on lung exam; who was initially diagnosed with extrapulmonary TB//with pleural fluid showing 6427 cells/mm3 with monocytic predominance (69%)//w subsequent development of swelling over left lower chest and lower back; with peripheral eosinophilia //with contrast chest CT showing b/l pleural effusion and heterogeneous left posterior muscle and paraspinal edema//With a diagnosis of acute *Toxoplasma* infection affecting the muscles and lungs made based on elevated *Toxoplasma* IgM antibodies on two occasions, two-fold rise in *Toxoplasma* IgG antibodies//Child clinically responded to anti- *Toxoplasma* treatment (given x 7 weeks)//At 3 wks f/up there was resolution of fever, weight gain and resolution of left lower chest swelling and at 7 wks f/up there was resolution of pleural effusions and normalization of eosinophilia
	
Chiappe Gonzalez 2020	Toxoplasmic myopericarditis and possible encephalitis, in a 34-yr-old male who presented w 7 ds Hx of progressive HA, photophobia, fever, weakness, myalgias, arthralgias, maculopapular rash, generalized LADP, hepatomegaly//with normal Brain MRI and CSF examination//w elevated troponin and CK-MB, transaminitis, ESR and beta 2 microglobulin//w cardiac MRI confirming asymptomatic myocarditis with subpericardial LGE and mild pericardial effusion with normal ventricular function//With positive *Toxoplasma* IgG and *Toxoplasma* IgM (and increasing *Toxoplasma* IgM in the f/up)//with positive *Toxoplasma* PCR from the LN biopsy//w clinical, Laboratory and cardiac MRI improvement in response to anti-*Toxoplasma* treatment (TMP/SMX x 4 weeks initially and then Azithromycin + Clindamcyin X another 2 wks due to TMP/SMX induced neutropenia)//With no relapse of symptoms after >12 mo of f/up
Cortes 2020	Multiorgan failure, with pneumonia/Respiratory failure requiring ICU admission (also myopericarditis, nephritis, FUO, ocular toxoplasmosis, splenomegaly and LADP); in a 44 Y M from Colombia without travel to Amazonian regions, who required prolonged hospitalizations x 32 ds //who presented w FUO, profuse sweating, HA, generalized malaise; weight loss, diarrhea////W persistence of systemic symptoms but w resolution of respiratory failure and without any need for IMV at that point-after the first 10 ds, even PRIOR to initiation of anti- *Toxoplasma* treatment ///who had complete improvement of systemic condition only after initiation of anti- *Toxoplasma* treatment with TMP/SMX///W identification of positive *Toxoplasma* IgG and IgM after the development of ocular findings; and also positive *Toxoplasma* PCR from the vitreous fluid
Cortes 2020	Pneumonia with respiratory failure requiring ICU admission (also myopathy, ocular Toxoplasmosis, LADP mediastinal and intraabdominal);in a 67-yr-old male from Bogota without previous travel to Amazonian regions////in whom the diagnosis of Toxoplasmosis was made after the development of ocular findings 2 months after initial presentation of respiratory failure (apparently with resolution of respiratory failure prior to initiation of anti- *Toxoplasma* treatment)//Details for the response to anti- *Toxoplasma* treatment with TMP/SMX were not provided (except for the response of the ocular findings but not the systemic findings)//The ocular findings (decreased of visual acuity) led to the *Toxoplasma* diagnosis; with positive *Toxoplasma* PCR in vitreous fluid ///Serotyping of *T.gondii* identified an non Type II stain
Leroy 2020	Pneumonia and pleural effusion//in a 23-yr-old immunocompetent male (HIV negative) of French nationality who had recently visited Ivory Coast and had Hx of eating Raw meat, who presented with fever and malaise, LADP, hepatosplenomegaly, myalgias, chest pain and developed pneumonia with dyspnea; with elevated inflammatory markers, mild transaminitis, high CPK//with Chest/Abdomen CT showing micronodular infiltrates in both lungs, multiple LN mesenteric and retroperitoneal//w PET scan showing diffuse pulmonary infiltrates intensely metabolic; hypermetabolic splenomegaly and LADP//With Serologic diagnosis of Toxoplasmosis 13 ds after onset of symptoms //with seroconversion from initially negative *Toxoplasma* IgG and IgM to positive *Toxoplasma* IgG and IgM, and Low avidity; and positive also *Toxoplasma* PCR in blood/BAL and bone marrow//*T.gondii* genotype *Africa 1//*Patient treated with P/S x 6 wks and had complete clinical cure without sequelae
Leroy 2020	Post Infectious Acute Cerebellar ataxia (Static cerebellar syndrome), Rhombencephalitis (CN IV, VI & IX)//in a 34-yr-old immunocompetent male (HIV negative) of Lebanese nationality; who had recently visited Senegal, w H x of eating raw meat and unwashed fruits and vegetables//who presented with fever, malaise, diplopia, headaches, motor deficits and static cerebellar syndrome/ Rhombencephalitis (CN IV, VI &IX), myalgias//w transaminitis and mild CPK elevation//Normal Brain MRI// diagnosed with Toxoplasmosis within 10 ds from onset of symptoms //with seroconversion from negative *Toxoplasma* IgG to positive *Toxoplasma* IgG, *Toxoplasma* IgM and low avidity (Blood/CSF *Toxoplasma* PCR were negative)//Patient did NOT receive anti-*Toxoplasma* treatment; Pt received ONLY Steroids x 6 wks (for Post infectious neurologic presentation)//Patient had complete clinical cure after steroid treatment
Leroy 2020	Polymyositis complicated by motor deficit of all four limbs, leptomeningitis//in a 33-yr-old immunocompetent male of French nationality, who had recently visited Senegal and Hx of eating raw meat and unwashed fruits//who presented with fever, malaise, decreased visual acuity, headaches, myalgias, dyspnea//w significant transaminitis (AST/ALT 2268/1619 IU/L; unl <40) and significantly elevated CPK (77,000 IU/L; unl < 190)//w Brain MRI showing leptomeningitis//w Serologic diagnosis of Toxoplasmosis made ~1 month after onset of symptoms with positive *Toxoplasma* IgG, *Toxoplasma* IgM, *Toxoplasma* IgA and low avidity//Positive *Toxoplasma* PCR from muscle biopsy and aqueous humour//With serotyping of *T.gondii* strain Africa or Recombinant I/III//With Muscle Biopsy showing granulomatous inflammation and myocyte necrosis with bradyzoites positive also with immunostaining for *Toxoplasma* //Patient treated with P/S x 6 weeks and due to the chorioretinitis got also TMP/SMX x 1 year///Patient had complete clinical cure of systemic symptoms; except for the persistence of decrease in visual acuity, despite treatment
Martinot 2020	CNS- Brown-Sequard syndrome //in a 31-yr-old patient with spinal cord toxoplasmosis//Spinal MRI showed a 12-mm intramedullary C3–C4 cervical tumor on the left that had ring enhancement after a gadolinium injection, with normal Brain Imaging at the same time/////who had complete regression of all neurologic deficits after few weeks of pyrimethamine/sulfadiazine, and who after secondary prophylaxis x1 year had f/up MRI showing sequellar intramedullary image without contrast enhancement)
Chang 2019	CNS toxoplasmosis with Rapid progressive cognitive decline within 1 month//in a 64-yr-old immunocompetent man //who presented with fever and mental status changes, wide-base gait and right -side deviation//Brain MRI showed lesion in Let internal capsule and thalamus////Spinal tap showed elevated opening pressure; CSF protein was elevated (50 mg/dl) without CSF pleocytosis //Positive *Toxoplasma* IgG in blood and CSF (no results for *Toxoplasma* IgM in serum were given)////Started on TMP/SMX, but his condition deteriorated; until he was switched to Pyrimethamine; with regression also of brain MRI lesions on f/up MRI //(patient had regularly fed with cats for years)
Leveque 2019	Myopericarditis//in a 23-yr-old immunocompetent man from France, presented with a 2 ds Hx of intense chest pain typical of pericarditis//ECHO and EKG were normal but troponin was elevated (31.3 ng/l; unl < 14) and CRP was elevated too//Toxoplasma serology was negative, but *Toxoplasma* PCR in blood was positive//Cardiac symptoms self-resolved with anti-inflammatory medications after 3 ds; WITHOUT anti- *Toxoplasma* treatment//Cardiac MRI at f/up showed evidence of myocarditis //(Blood *Toxoplasma* PCR became negative after 2 wks of anti- *Toxoplasma* treatment and *Toxoplasma* IgG and IgM became positive (confirming recent seroconversion)
Basit, Nasir et al. 2018	CNS Toxoplasmosis infection ///20-yr-old immunocompetent male (HIV negative) from Pakistan, with 3 wks Hx of fever and Unilateral weakness, with gradual development of ALOC, neck stiffness, hypertonia, weakness in R upper and lower libs; CSF without pleocytosis or elevated protein//Brain MRI with multiple ring enhancing lesions in white and grey matter, with surrounding vasogenic edema on FLAIR images//Had high positive *Toxoplasma* IgG and IgM//Started treatment with TMP/SMX + Steroids//F/up MRI 2 wks later showed decrease in size of brain lesions and of the surrounding edema///
Cai 2018	NMDA encephalitis///in a 9-yr-old immunocompetent who presented with seizures, headaches and vomiting CSF and Brain MRI initially normal and self-recovered without any treatment //1 weeks later development of unexplained personality and behavior changes, recurrence of seizures and fever//Repeated CSF with lymphocytic pleocytosis and positive anti-NMDAR antibody. //Had positive *Toxoplasma* IgM and IgG.//Diagnosed with anti-NMDA encephalitis associated with acute acquired *toxoplasma* gondii infection//Treated with 10 days azithromycin with substantial recovery from clinical symptoms (immunotherapy was been refused)//F/up 2 mo later patient completely asymptomatic
Cortes 2018	Disseminated disease, with rapid neurologic deterioration and multiorgan failure that led to death in a 72-yr-old immunocompetent patient from Chocó, Colombia; who presented with a 12-day hx of fever, headache//with Brain CT showing hydrocephalus and fronto-parietal periventricular hypodensities //with histopathologic findings at autopsy showing tissue cysts morphologically suggestive of being bradyzoites of *Toxoplasma* gondii; confirmed by immunohistochemistry in heart, brain, and striated muscle.
Henao-Martinez, Montoya 2018	Myocarditis (and mild transaminitis and bilateral retinitis)//in a 29-yr-old immunocompetent woman (HIV negative) from the US (after a trip to Nicaragua x 10 ds; ate undercooked meat during the trip)//presented with acute onset of fever, retroorbital headache, myalgias and rash//LADP also developed 12 ds later//, with transaminitis, anemia and lymphocytosis//Continued fevers, malaise and had new onset of blurry vision within 1 mo into her illness// *Toxoplasma* IgG and IgM were high positive; *Toxoplasma* IgA and IgE high positive//Troponin was mildly elevated (0.26; unl < 0.05 ng/mL)//Started TMP/SMX; and switched to Clindamycin (without Pyrimethamine) due to severe nausea with TMP/SMX //Got anti- *Toxoplasma* treatment for 8 wks and symptoms gradually resolved (including resolution of retinal findings), & troponin normalization (for ocular findings also received intravitreal clindamycin)
Henao-Martinez, Montoya 2018	Myocarditis and Transaminitis//in a 30-yr-old immunocompetent male from the US (after a family trip to Nicaragua x 10ds, having eaten undercooked meat during the trip)//presented with fatigue, myalgias and bilateral conjunctivitis; transaminitis//Had high positive *Toxoplasma* IgG, High positive *Toxoplasma* IgM and igA and low avidity//mildly elevated troponin (0.1 ng/mL; unl < 0.05)//Due to persistence of symptoms got TMP/SMX x 2 weeks and his symptoms resolved completely//(*Per authors conclusion: Acute toxoplasmosis remains an important diagnostic consideration among travelers with acute illness//Acute toxoplasmosis in immunocompetent patients with evidence of end organ injury is an indication for anti- Toxoplasma* treatment)//
Akturk, Sotello et al. 2017	Toxoplasmic encephalitis with multiple bilateral ring enhancing brain lesions in an immunocompetent 32-yr-old male with increasing agitation and confusion, personality changes and Hx of recurrent headaches with onset of HA few months PTA//Brain CT showing multifocal white matter lesions.//with Brain MRI showing extensive bilateral cortical and subcortical concentric ring-enhancing white matter lesions throughout both cerebral hemispheres; with lesions were more prominent in left parietal and frontal lobes//Treatment with steroids for presumed multiple sclerosis caused worsening of symptoms and increase in size of the brain MRI lesions//Subsequently, brain biopsy confirmed *T.gondii* infections by histopathology and PCR and treatment with Pyrimethamine/Sulfadiazine decreased the size of the brain lesions dramatically (*case of severe Toxoplasmosis due to inappropriate administration of high dose steroids for mis-diagnosis for multiple Sclerosis- At the time of Toxoplasma serology the serologic profile was non-acute; however given the clinical presentation few months earlier, the Toxoplasma serology results at the time of testing are not inconsistent with an acute infection -few months earlier-at the time of clinical presentation)*
Pustorino 2017	Cerebral Toxoplasmosis //in a 30-yr-old immunocompetent man (HIV negative) from Italy with a 5 yr hx of monthly frontal headache, with worsening intensity and frequency over the last 1 month, with associated episodes of sudden falls without loss of consciousness, followed by unjustified crying//30 yr old immunocompetent man with Hx of after eating poorly cooked pork meat ///Brain CT showing multiple calcifications involving the basal ganglia and semioval centers//Brain MRI T2 weighted images showing multiple cystic lesions surrounded by brain edema, non enhancing after contrast///EEG showing mild slowing over left temporal region//no follow up for 20 mo, but due to daily severe headaches and secondary generalized seizures was readmitted wth Brain CT and MRI showing marked size increase of cystic lesions with ring enhancement and diffuse perilesional edema with midline shift//S/P emergency surgical removal of 2 large brain cysts, histopathologically showing several bradyzoites and positive *T.gondii* DNA PCR//with initiation of anti-*Toxoplasma* treatment with pyrimethamine/sulfadiazine + Dexamthasonze (later changed to pyrimethamine/clindamycin due to rash with sulfadiazine) (not more clinical details given)//A 20 mo f/up there was resolution of Headaches and persistence of sporadic partial motor seizures and f/up brain MRI showing mild decease in size of cystic lesions with reduction of perilesional edema and contrast enhancement
Beltrame, Venturini et al. 2016	Seizures secondary to toxoplasmosis////15-yr-old immunocompetent boy, with LADP and recurrent seizures, after vacation to Ethiopia, with EEG showing sporadic single spike or sharp-wave paroxysms//with *T.gondii* serology consistent with acute primary infection//Brain MRI was normal//with normalization of the EEG after 4 weeks of anti- *Toxoplasma* treatment with P/S/FA//with recurrence of seizures 5 months later, with reappearance of fatigue and cervical LADP//with negative repeat brain MRI///with elevated CSF protein level (250 mg/dl; with the rest of the CSF analysis normal//with negative CSF *Toxoplasma* PCR at which time a diagnosis of epilepsy was made and seizures were controlled with valproic acid (this case represented the first reported case of seizures during acute primary toxoplasmosis in immunocompetent patient)
Bousquet 2016	Acute myocarditis/ in a 20 yr old immunocompetent military man in Begin, with 2 ds Hx of headaches, myalgias and malaise (afebrile)//EKG with incomplete RBBB, very elevated Troponin and CPK; (suspected Acute MI but coronary angiography was normal)//Cardiac MRI: areas of myocardial edema//*Toxoplasma* serology showed seroconversion (initially positive *Toxoplasma* IgM, negative Ig and 2 wks later, positive *Toxoplasma* IgG and IgM)// treated with TMP/SMX x ~2 weeks//Patient fully recovered (in response to treatment) with no complications (except for significant asthenia × 3 month) (He was considered unfit for the operational field)
Harbada 2016	Toxoplasma cerebritis and encephalitis///23-yr-old immunocompetent male residing in Oman with a 2 wks Hx of weakness, incoordination of R upper and lower extremities, 5 ds Hx of throbbing headache, and visual blurring//Brain MRI T2 weighted images showing ill-defined hyperintense lesion in Right cerebellar hemisphere with perilesional edema, extending along the right foramen of Luschka///Post Contrast T1 images showing heterogeneous post-contrast enhancements with peripheral enhancing ring ///Underwent surgical excision due to concern for malignant space occupying lesions///histopathology showed necrosis, hemorrhage, perivascular infiltrates, *T.gondii* cysts and evidence of cerebritis and encephalitis///treated with Clindamycin + Azithromycin due to G6PD deficiency//post surgery neurologic status deteriorated and Brain MRI showing increase in perilesional edema and inflammation involving the brain stem (Steroid doses were increased//Patient clinically deteriorated despite anti- *Toxoplasma* treatment and died the 10th postsurgical day due to spreading *Toxoplasma* cerebritis and encephalitis)
Hoti 2016	Toxoplasmic encephalitis //in a 45-yr-old immunocompetent HIV negative woman from Pakistan, with headaches and vomiting x 2 months; with confusion, behavioral problems, weakness in R upper limb//Brain CT showing a hypodense lesion in left Frontoparietal region; with Brain MRI T1 images showing a hypodense lesion in left parietal area; contrast enhancing in T1 contrast images; and T2 hyperintense//S/P total surgical excision of the mass due to concern for neoplasm; with Brain histopathology showing inflammatory features of toxoplasmosis in cerebral cortex and grey matter, with bradyzoite encased within cysts; Positive *Toxoplasma* IgG and IgM//Post surgery her Right upper limb weakness grossly improved and speech and higher motor functions remained intact//Was treated with TMP/SMX x 14 ds and 6 months post OP her condition was stable//No immunosuppression concern at 1 year of f/up.
Manwani 2016	CNS toxoplasmosis//in a 14-yr-old immunocompetent boy (HIV negative × 2) from India with a 10 ds Hx of difficulty closing R eye, deviation of angle of the mouth on the left, nasal regurgitation of feeds and unsteady wide-based gait; central R facial nerve palsy, with involvement also of the IX and X cranial nerves//Brain MRI showing a hypodense lesion in the pons extending into the medulla, with heterogeneous enhancement with contrast, with vague ring enhancement dorsally, with initially concern for Tuberculoma//Due to progressive increasing difficulty in swallowing, absent gag reflex and R hemiparesis, underwent surgical excision of the brain mass that showed zones of necrosis, thrombosed vessels and aggregate large histiocytes with a single *T.gondii* bradyzoite with positive *Toxoplasma* immunostaining//Positive *Toxoplasma* IgM//Despite initiation of TMP/SMX there was NO neurologic improvement within 1 week of treatment; w subsequent development brain stem dysfunction and eventually cardiac arrest and death 2 weeks after anti- *Toxoplasma* treatment initiation.
Azarpira 2014	ABSTRACT: Cerebral toxoplasmosis is the most important opportunistic infection in patients with acquired immunodeficiency syndrome. However, it is a rare finding in immunocompetent persons. The patient was a 14 yr-old boy who presented with headache and vertigo. Toxoplasma serology revealed raised IgG antibody level. No primary or secondary immune deficiency was found. Radiologic examination revealed ventriculitis. Microscopically, the lesion consisted of reactive gliosis and calcification. After antitoxoplasma treatment, the patient condition improved. Brain toxoplasmosis usually presented as periventricular/basal ganglial lesions. We report a case of a child patient with an atypical pattern of toxoplasma encephalitis, presenting with ventriculitis.
Li 2014	CNS toxoplasmosis//in a 33-yr-old immunocompetent male from China with 9 month Hx of dysphagia and numbness of left limb x 1 mo; with decreased sensation in R upper and lower extremities, expressive aphasia, positive Babinski on the R, papilledema, //Brain CT showing an isodense lesion with surrounding edema//Brain MRI showing a space occupying lesion, isointense in T1 and Hypointense in T2 and adjacent cerebral edema//Post contrast MRI showing mushroom-shaped lesions with irregular enhanced rim //Serum and CSF *Toxoplasma* IgG were positive//Underwent total mass removal and his preoperative neurologic symptoms improved//Histopathology of the excised brain mass showed parasite within a pseudocyst structure; with positive *Toxoplasma* immunostaining//Was treated with Azithromycin (initially IV and then PO × 4 weeks) with dramatic clinical and radiologic improvement; with no recurrence in f/up MRI 2.5 yrs post OP)
Paruthikunnan 2014	Disseminated toxoplasmosis with pulmonary and CNS involvement//in a 28-yr-old immunocompetent (HIV negative) pregnant woman from India, with intermittent fever and cough × 1 month; with elevated inflammatory markers, Eosinophilia (18%)//Patient had an abortion at the time of high fever//CXR showed multiple ill-defined nodular opacities in both lungs and patchy areas of consolidation in R upper and mid zones//Chest CT showing an area of consolidation the RUL and multiple nodules with GGO haloes around them-There was no pleural effusion and no significantly enlarged mediastinal LN//Due to high suspicion for TB initially; patient was treated with anti-TB therapy///Subsequently patient became drowsy with deteriorating level of consciousness//Brain MRI showed an irregular ring enhancing lesion in the R globus pallidus and R internal capsule; Hypointense on T1 and Hyperintense in T2 images/ with ADC (apparent Diffusion coefficient) images showing that the lesion has a peripheral rim of restricted diffusion with a central area of facilitated diffusion//Postcontrast coronal T1-weighted images on admission show the same lesion enhancing peripherally and another enhancing nodular lesion in the right superior frontal gyrus //Excisional biopsy of a Cervical LN showed T. gondii Trophozoites on histopathology//Strongly Positive *Toxoplasma* IgG and IgM//Started on anti- *Toxoplasma* treatment with P/S and Clindamycin and her clinical condition improved significantly in response to therapy//F/up CXR after 1 mo of anti- *Toxoplasma* therapy showed resolution of lung opacities//Examination of placenta and abortus showed the presence of placental and congenital toxoplasmosis in the fetus///F/up Chest CT 4.5 mo later also showed resolution of consolidation and nodules with bronchiectatic changes seen in the segment previously affected by consolidation//Repeat Brain MRI after 5 months also showed near complete resolution of the brain lesion, with some gliotic changes in the R basal ganglia
Ramachandran 2014	CNS toxoplasmosis//in a 42-yr-old immunocompetent male (HIV negative) from India, with Headache and neck pain x 1 week; weight loss X 1 month, but NO fever; with elevated inflammatory markers//with Brain MRI showing multiple large bilateral lesions that appeared hypotense in T1 images and hyperintense in T2/FLAIR sequences//with intense homogeneous enhancement post contrast administration//Patient’s symptoms worsened over the next 3 ds //Repeat head CT showed multiple homogeneous enhancing hyperdense lesions with surrounding peri-legional edema in both cerebral hemispheres concerning for CNS lymphoma///Brain MRI the same day showed new lesions in the left Thalamus and globus pallidus with restricted diffusion on DWI images with los signal in ADC images suggestive of true restriction//MR spectroscopy showed elevated Lipid lactate peak///In view of the multiplicity of brain lesion and the onset of newer lesions within a span of one week, the possibility of CNS Toxoplasmosis was raised//CSF *Toxoplasma* IgG and IgM were Highly positive//Patient left against medical advice and subsequently died due to respiratory failure///
Undseth 2014	Multi-organ failure; pneumonitis, myocarditis, hepatitis, and meningoencephalitis//in a 39-yr-old immunocompetent man (HIV negative multiple times) from Norway, with Disseminated Toxoplasmosis with mutliorgan involvement with pneumonia, myocarditis, hepatitis, meningoencephalitis//who presented with Headache, High Fever and Diarrhea, generalized LADP//CSF with slight pleocytosis (11 cells/mm3)//During a LN excision procedure became respiratory and circulatory unstable and transferred to a regional hospital with Hypoxia (needing 70% FiO2), w high fevers//Required Inotropes and subsequently also mechanical Ventilation x 11 ds total/ Corticosteroids were added initially to his antimicrobial regimen//Had elevated inflammatory markers (CRP = 186 mg/dl; unl < 10.0) thrombocytopenia, Very elevated CPK and LDH, transaminitis and elevated γGT, Very elevated Ferritin (27,220 μg/l) and elevated Troponin; Low CD4 (133 cells/μL) and CD8 cells///Initial Brain MRI was normal but second MRI showed meningeal enhancement without intracerebral pathology//Patient was consistently confused and disoriented for a long period////*Toxoplasma* serology with high positive *Toxoplasma* IgM and *Toxoplasma* IgG and low avidity & positive *Toxoplasma* blood PCR & positive CSF *Toxoplasma* PCR//All biopsies (3 LN and Bone marrow biopsies (were positive for *Toxoplasma* cysts by microscopy; with the first POSITIVE results for Toxoplasmosis became available on HD #8//Haemophagocytosis was described//Was started on IV TMP/SMX //Once toxoplasmosis diagnosis was made on d#8 and anti-*Toxoplasma* treatment was started, clinical improvement was seen a few ds later///By D#22 he was overall more stable, LADP had decreased in size; but continued to have high fevers for another 16 ds & had persistent tachycardia with elevated troponin//On d#72 was transferred to a rehabilitation unit//After 1 year he was fully rehabilitated and back to full time work///Shortly after this developed visual disturbances and diagnosed with *Toxoplasma* chorioretinitis and anti-*Toxoplasma* treatment was resumed//The patient’s PLTs normalized after 3 wks of TMP/SMX//The patient had high Ferritin in serum and erythrophagocytosis in LN biopsy; however ferritin levels fell from 27, 220 to 1549 μg/L after 5 ds of TMP/SMX and also LDH decreased from 2663 to 657 IU/L)//*RISK factors: had recently travelled across the Greenland glacier under quite challenging conditions and possibly consumed undercooked food//*Primary infection was treated x7 wks and did NOT receive secondary prophylaxis; However was placed on long term secondary prophylaxis after the ocular toxoplasmosis reactivation 1 year later///
De Souza Giassi 2014	Acute Respiratory failure in a 36-yr-old male (w Hx of type 2 DM) secondary to acute toxoplasmosis with extensive lung involvement; who presented w 2 wks febrile illness, headache and progressive dyspnea few ds prior to admission; w crackles in lung exam, hypoxia to 82–86%; splenomegaly and in Lab values: lymphocytosis, transaminitis and elevated inflammatory markers//Chest CT w diffuse GGO, peribroncho-vascular and septal thickening and nodules, and small pleural effusions//Patient responded to anti-*Toxoplasma* treatment with complete resolution of clinical symptoms without subsequent relapse///Complete resolution also of Chest CT findings after 3 mo of reatment
De Souza Giassi 2014	Acute Respiratory failure in a 56-yr-old female (w Hx of type 2 DM) secondary to acute toxoplasmosis with extensive lung involvement//who presented w 2 wks febrile illness, headache and progressive dyspnea few ds prior to admission; w crackles in lung exam, hypoxia to 82–86%; splenomegaly and in Lab values: lymphocytosis, transaminitis//Chest CT w diffuse GG and peribrochovascular and septal thickening AND mediastinal paratracheal LN enlargement//Patient responded to anti-*Toxoplasma* treatment with complete resolution of clinical symptoms without subsequent relapse
De Souza Giassi 2014	Acute Respiratory failure in a 38-yr-old-female secondary to acute toxoplasmosis with extensive lung involvement who presented w 2 wks febrile illness, dyspnea and cough; w crackles in lung exam, hypoxia to 82–86%; splenomegaly and in Lab values: lymphocytosis, transaminitis//Chest CT w diffuse GGO, peribroncho-vascular and septal thickening and atelectasis and small pleural effusions//Patient responded to anti-Toxoplasma treatment with complete resolution of clinical symptoms without subsequent relapse///
Abhilash, Roshine 2013	High grade intermittent fever of 6 months duration, generalized arthralgia, significant weight loss, cervical and axillary LADP, splenomegaly (4 cm below the left costal margin.)/////in a 32-yr-old immunocompetent HIV negative woman///with LN biopsy showing reactive follicular hyperplasia//Bone Marrow biopsy showing multiple oval cysts suggestive of *T.gondii //Toxoplasma* IgM strongly positive and Toxoplasma IgG positive//Patient responded to pyrimethamine and clindamycin treatment x 2 wks with resolution of fevers and regression of LADP and splenomegaly at the 3 months f/up
Aksoy, Tanir 2013	Diagnosis of acute disseminated encephalomyelitis (ADEM) in a 10-yr-old immunocompetent boy (HIV negative x2) with difficulty walking and urinary and stool incontinence//with normal inflammatory markers, diagnosed with ADEM in the context of recent acute primary Toxoplasmosis infection (with case presenting as the pure myelitis form of ADEM, with imaging findings nevertheless in both brain and spinal cord-but without encephalopathy////with CSF analysis showing pleocytosis, lymphocytosis (210 lymphs/mm3); mildly elevated CSF protein (54 mg/dl)///FLAIR MRI showing high intensity lesions in the periventricular white matter, corona radiata, semiovale center and left parietal lobe; no mass effect and no contrast enhancement//All brain lesions were hyperintense in DWI and ADC(apparent diffusion coefficient) images AND Spinal MRI showing diffuse abnormal signal intensity C4-T6 level with no enhancement (imaging findings consistent with ADEM)///Boy was treated with steroids (high dose for first 5 ds) and on d3 of steroid there was significant reduction in the neurologic findings and on d7 there was complete resolution//The positive serologic results for Toxoplasmosis became available 2 wks after admission (after the improvement of symptoms on steroids only) and was treated with P/S x 4 wks (Patient had a 4-fold rise in *Toxoplasma* IgG titers over a 3 wk interval, with positive *Toxoplasma* IgM and low IgG avidity, indicating recent API//F/up MRI 6 mo later showed disappearance of the initial lesions AND child without neurologic findings
Sobanski 2013	Pulmonary toxoplasmosis, severe retinochoroiditis//in a 47-yr-old immunocompetent (HIV negative) man (case from France); who presented with high fever, dyspnea and flu like symptoms (no duration of symptoms PTA was given); night sweats, N/V, rash and generalized LADP//Hypoxia (92% O2 Sats)//CXR diffuse interstitial infiltrates; Transaminitis; high LDH, lymphocytosis//*Toxoplasma* serology with seroconversion: initially *Toxoplasma* IgM positive, *Toxoplasma* IgG negative and 2 mo later *Toxoplasma* IgG positive (and IgM negative at that time)//Chest CT at that stage showed mediastinal LADP WITHOUT parenchymal infiltrates//BAL: positive *Toxoplasma* DNA PCR// treatment initiated with Spiramycin X 3 weeks (HD #12) and clinically improved within 3 ds (with defervescence)////Relapse of clinical status 1 mo later with recurrence of fevers, night sweats and weight loss AND decreased Visual acuity//PET scan (2 mo after symptoms onset) showed increased LN uptakes AND Inguinal LN biopsy (2 mo after symptoms onset) was positive for *Toxoplasma* DNA PCR (indicating ineffectiveness of Spiramycin treatment)//At that time treated with Pyrimethamine + Clindamycin x 6 weeks with clinical response except for only partial recovery of eyesight///Genotype showed Atypical genotype (Risk: consumption of raw horse meat from Brazil and Canada
Yang 2013	Multi-organ failure, Respiratory failure, HLH, disseminated toxoplasmosis//in a 41-yr-old immunocompetent (HIV negative) male from China; who presented with a 4 d Hx of fever, malaise, dry cough, confusion//Along with high fever, HSM, cytopenias, hyperferritinemia, hypofibrinogenemia, hypertriglyceridemia suggestive of Hemophagocytic syndrome (leukopenia (WBC 2290/ μL) and Thrombocytopenia (PLT = 9.000/μL), transaminitis, elevated LDH, very high Ferritin (23,800μg/L) and high TG; //CXR with interstitial and alveolar infiltrates//HSM//(Risk factors: forest policeman/ hunter, drinking stream water)//Clinical condition rapidly worsened with respiratory failure and need for IMV on HD 3; MOF developed on HD 8 and patient died//diagnosis of Toxoplasmosis made postmortem with Bone Marrow Bx showing large amount of *T.gondii* tachyzoites and Cysts and positive *Toxoplasma* PCR//
Demar 2012	22-yr-old male from French Guiana, admitted in ICU for B/L interstitial pneumonia, no IMV, hypoxia, Chest CT with Bilateral interstitial pneumonia, Cardiomegaly, pericarditis///ABSTRACT: A newly described form of toxoplasmosis, ‘Amazonian toxoplasmosis’ (AT), has been reported since 2002 in French Guiana. It is characterized by severe cases and atypical strains linked to a neotropical forest-based cycle. We report on the cases of AT that required intensive care management. We performed a prospective observational study on hospitalized adults in the Intensive Care Unit (ICU) from 2002 to 2008. Clinical and laboratory data, microbiological findings and outcomes were recorded. Data, including the ICU simplified acute physiology score and the pneumonia severity index, were calculated. Epidemiological risk factors for AT were assessed through questionnaires. Eleven non-immunodeficient patients were admitted to the ICU in Cayenne for life-threatening pneumonia associated with disseminated toxoplasmosis.
Demar 2012	41-yr-old male from French Guiana, admitted in ICU, for hypoxia, B/L interstitial pneumonia//ABSTRACT: A newly described form of toxoplasmosis, ‘Amazonian toxoplasmosis’ (AT), has been reported since 2002 in French Guiana. It is characterized by severe cases and atypical strains linked to a neotropical forest-basedcycle. We report on the cases of AT that required intensive care management. We performed a prospective observational study on hospitalized adults in the Intensive Care Unit (ICU) from 2002 to 2008. Clinical and laboratory data, microbiological findings and outcomes were recorded. Data, including the ICU simplified acute physiology score and the pneumonia severity index, were calculated. Epidemiological risk factors for AT were assessed through questionnaires. Eleven non-immunodeficient patients were admitted to the ICU inCayenne for life-threatening pneumonia associated with disseminated toxoplasmosis.
Demar 2012	36-yr-old male from French Guiana, admitted to ICU, needed IMV for hypoxia, B/L interstitial pneumonia//ABSTRACT: A newly described form of toxoplasmosis, ‘Amazonian toxoplasmosis’ (AT), has been reported since 2002 in French Guiana. It is characterized by severe cases and atypical strains linked to a neotropical forest-based cycle. We report on the cases of AT that required intensive care management. We performed a prospective observational study on hospitalized adults in the Intensive Care Unit (ICU) from 2002 to 2008. Clinical and laboratory data, microbiological findings and outcomes were recorded. Data, including the ICU simplified acute physiology score and the pneumonia severity index, were calculated. Epidemiological risk factors for AT were assessed through questionnaires. Eleven non-immunodeficient patients were admitted to the ICU in Cayenne for life-threatening pneumonia associated with disseminated toxoplasmosis.
Demar 2012	39-yr-old male from French Guiana, admitted to ICU, needed IMV for hypoxia, B/L interstitial pneumonia //ABSTRACT: A newly described form of toxoplasmosis, ‘Amazonian toxoplasmosis’ (AT), has been reported since 2002 in French Guiana. It is characterized by severe cases and atypical strains linked to a neotropical forest-based cycle. We report on the cases of AT that required intensive care management. We performed a prospective observational study on hospitalized adults in the Intensive Care Unit (ICU) from 2002 to 2008. Clinical and laboratory data, microbiological findings and outcomeswere recorded. Data, including the ICU simplified acute physiology score and the pneumonia severity index, were calculated. Epidemiological risk factors for AT were assessed through questionnaires. Eleven non-immunodeficient patients were admitted to the ICU in Cayenne for life-threatening pneumonia associated with disseminated toxoplasmosis.
Demar 2012	19-yr-old male from French Guiana, admitted to ICU, needed IMV, for hypoxia, pneumonia “white lung”, meningismus//ABSTRACT: A newly described form of toxoplasmosis, ‘Amazonian toxoplasmosis’ (AT), has been reported since 2002 in French Guiana. It is characterized by severe cases and atypical strains linked to a neotropical forest-based cycle. We report on the cases of AT that required intensive care management. We performed a prospective observational study on hospitalized adults in the Intensive Care Unit (ICU) from 2002 to 2008. Clinical and laboratory data, microbiological findings and outcomes were recorded. Data, including the ICU simplified acute physiology score and the pneumonia severity index, were calculated. Epidemiological risk factors for AT were assessed through questionnaires. Eleven non-immunodeficient patients were admitted to the ICU in Cayenne for life-threatening pneumonia associated with disseminated toxoplasmosis.
Demar 2012	30-yr-old male from French Guiana, admitted to ICU, needed IMV for hypoxia, B/L interstitial pneumonia; had cardiac arrhythmia//ABSTRACT: A newly described form of toxoplasmosis, ‘Amazonian toxoplasmosis’ (AT), has been reported since 2002 in French Guiana. It is characterized by severe cases and atypical strains linked to a neotropical forest-based cycle. We report on the cases of AT that required intensive care management. We performed a prospective observational study on hospitalized adults in the Intensive Care Unit (ICU) from 2002 to 2008. Clinical and laboratory data, microbiological findings and outcomes were recorded. Data, including the ICU simplified acute physiology score and the pneumonia severity index, were calculated. Epidemiological risk factors for AT were assessed through questionnaires. Eleven non-immunodeficient patients were admitted to the ICU in Cayenne for life-threatening pneumonia associated with disseminated toxoplasmosis.
Demar 2012	38-yr-old male from French Guiana, admitted to ICU, needed IMV for B/L interstitial pneumonia//ABSTRACT: A newly described form of toxoplasmosis, ‘Amazonian toxoplasmosis’ (AT), has been reported since 2002 in French Guiana. It is characterized by severe cases and atypical strains linked to a neotropical forest-based cycle. We report on the cases of AT that required intensive care management. We performed a prospective observational study on hospitalized adults in the Intensive Care Unit (ICU) from 2002 to 2008. Clinical and laboratory data, microbiological findings and outcomes were recorded. Data, including the ICU simplified acute physiology score and the pneumonia severity index, were calculated. Epidemiological risk factors for AT were assessed through questionnaires. Eleven non-immunodeficient patients were admitted to the ICU inCayenne for life-threatening pneumonia associated with disseminated toxoplasmosis.
Demar 2012	28-yr-old female from French Guiana, admitted to ICU, with B/L interstitial infiltrated, had myopericarditis, cardiomegaly, low EF 26%, Hypokinesia/ABSTRACT: A newly described form of toxoplasmosis, ‘Amazonian toxoplasmosis’ (AT), has been reported since 2002 in French Guiana. It is characterized by severe cases and atypical strains linked to a neotropical forest-based cycle. We report on the cases of AT that required intensive care management. We performed a prospective observational study on hospitalized adults in the Intensive Care Unit (ICU) from 2002 to 2008. Clinical and laboratory data, microbiological findings and outcomes were recorded. Data, including the ICU simplified acute physiology score and the pneumonia severity index, were calculated. Epidemiological risk factors for AT were assessed through questionnaires. Eleven non-immunodeficient patients were admitted to the ICU in Cayenne for life-threatening pneumonia associated with disseminated toxoplasmosis.
Demar 2012	22-yr-old female from French Guiana, admitted to ICU, needed IMV for hypoxia, B/L interstitial pneumonia, had pericarditis//ABSTRACT: A newly described form of toxoplasmosis, ‘Amazonian toxoplasmosis’ (AT), has been reported since 2002 in French Guiana. It is characterized by severe cases and atypical strains linked to a neotropical forest-based cycle. We report on the cases of AT that required intensive care management. We performed a prospective observational study on hospitalized adults in the Intensive Care Unit (ICU) from 2002 to 2008. Clinical and laboratory data, microbiological findings and outcomes were recorded. Data, including the ICU simplified acute physiology score and the pneumonia severity index, were calculated. Epidemiological risk factors for AT were assessed through questionnaires. Eleven non-immunodeficient patients were admitted to the ICU in Cayenne for life-threatening pneumonia associated with disseminated toxoplasmosis.
Demar 2012	24-yr-old male from French Guiana, admitted to the ICU for hypoxia and B/L interstitial pneumonia; had meningismus//ABSTRACT: A newly described form of toxoplasmosis, ‘Amazonian toxoplasmosis’ (AT), has been reported since 2002 in French Guiana. It is characterized by severe cases and atypical strains linked to a neotropical forest-based cycle. We report on the cases of AT that required intensive care management. We performed a prospective observational study on hospitalized adults in the Intensive Care Unit (ICU) from 2002 to 2008. Clinical and laboratory data, microbiological findings and outcomes were recorded. Data, including the ICU simplified acute physiology score and the pneumonia severity index, were calculated. Epidemiological risk factors for AT were assessed through questionnaires. Eleven non-immunodeficient patients were admitted to the ICU in Cayenne for life-threatening pneumonia associated with disseminated toxoplasmosis.
Demar 2012	11-yr-old male from French Guiana, admitted to ICU, needed IMV for hypoxia, B/L interstitial pneumonia, had cardiomegaly, pericarditis, low EFABSTRACT: A newly described form of toxoplasmosis, ‘Amazonian toxoplasmosis’ (AT), has been reported since 2002 in French Guiana. It is characterized by severe cases and atypical strains linked to a neotropical forest-based cycle. We report on the cases of AT that required intensive care management. We performed a prospective observational study on hospitalized adults in the Intensive Care Unit (ICU) from 2002 to 2008. Clinical and laboratory data, microbiological findings and outcomes were recorded. Data, including the ICU simplified acute physiology score and the pneumonia severity index, were calculated. Epidemiological risk factors for AT were assessed through questionnaires. Eleven non-immunodeficient patients were admitted to the ICU in Cayenne for life-threatening pneumonia associated with disseminated toxoplasmosis.
Groh 2012	Pneumonia, Congestive heart failure (CHF) (along with transaminitis, pancreatitis, hyponatremia and chorioretinitis) //in an immunocompetent (HIV negative) Chinese man living in French Guiana who presented with a 3 wk Hx of fever, dyspnea and confusion, weight loss//CXR with alveolar infiltrates //Had Tachycardia and findings of CHF, lung crackles in R lung, LADP and chorioretinitis and corneal ulcer//Had elevated cardiac markers, (Troponin and BNP), high LDH, Hyponatremia (Na nadir: 125; likely due to pneumonia associated SIADH), mild transaminitis & pancreatitis (high lipase) //High positive *Toxoplasma* IgG and IgM, low avidity and positive blood *Toxoplasma* PCR, positive mice subinoculation//Genotype analysis with 15 microsatellite markers showed Atypical *T.gondii* strain //ECHO showed low LVEF (35%) with global hypokinesia//Also w chorioretinitis in L eye suggestive of Toxoplasmosis (Risk: consumption of semi-raw Tapir 3 wks before onset of symptoms; *Toxoplasma* PCR was positive also from the frozen Maipour meat)//Patient responded to treatment with Pyrimethamin + Sulfadiazine (later sulfadiazine changed to clindamycin due to hepatotoxicity); anti-*Toxoplasma* treatment given x 6 weeks//
Kanno 2012	Toxoplasmic encephalitis//in a 81-yr-old immunocompetent woman from Japan with Right lower limb discomfort, who subsequently developed memory disturbances)//with Brain MRI FLAIR images showing a high signal lesion in Left parietal region///with f/up MRI showing enlargement of original lesion and development of new lesion contralaterally (along with Left lower limb Dyskinesia, myoclonus, involuntary limb movements incoherent speech; low CD4 cells 286/μL, HIV negative), normal CSF analysis; High Toxoplasma IgG Antibodies, negative *Toxoplasma* IgM and negative CSF *T.gondii* PCR)//with repeat brain MRI due to worsening level of consciousness showing Bilateral temporal and frontal lesions with T2 target signs (on T2 weighted image a three-layered structure with low signal, high signal, and low signal)//(Due to suspicion of *Toxoplasma* encephalitis Pyrimethamine + Sulfadiazine was started with gradual improvement in patient’s level of consciousness, disappearance of involuntary limb movements AND ultimately disappearance of brain MRI lesions //(*(T2 target sign (core is iso-signal or low signal, the exterior is a high signal and the outermost side is iso-signal or iso-signal); an abscess core with an iso-signal or low signal compared with normal white matter of diffusion weighted imaging and the core with high signal on ADC mapping: are distinct imaging findings of toxoplasmic encephalitis)*
Roubille 2012	Myopericarditis w mild pericardial effusion in a 24-yr-old male, who presented with acute chest pain and SOB (with mild pericardial effusion developing after onset of fever), with elevated inflammatory markers, elevated troponin (peak Troponin 17.4 μg/L; unl <0.05)//with cardiac MRI showing LGE (late gadolinium enhancement) and mild pericardial effusion consistent with the diagnosis of myocarditis//Patient’s symptoms had resolved prior to the Toxoplasmosis diagnosis and without anti-*Toxoplasma* treatment; although 4 wks from presentation patient still had elevated troponin (0.4μg/L) (of note patient refused initiation of anti- *Toxoplasma* treatment at that time)
Neves 2011	Disseminated toxoplasmosis//in a 41-yr-old immunocompetent man (HIV negative) from Brazil, presented with a 2 ds Hx of high fever, myalgias, severe headache and nausea; with transaminitis; with his condition rapidly deteriorating with development of dyspnea, cough, intense headache with meningeal signs, nausea and vomiting; cervical LADP during the 2nd week of illness; CXR showed B/L alveolar infiltrates//CSF analysis showed pleocytosis (35 cells/mm3) with lymphocytosis (85% lymphocytes)///Had positive Toxoplasma IgG and IgM with low avidity//Treated with Clindamycin + Pyrimethamine; with rapid clinical improvement; resolution of CXR findings; normalization of CSF findings and resolution of transaminitis//Remained asymptomatic at the 22 mo f/up
Galli Tsiropoulou 2010	The authors report a 5-yr-old boy who presented with cervical lymphadenopathy because of acquired toxoplasmosis accompanied with unilateral facial nerve paralysis. Toxoplasma gondii DNA detection in blood by polymerase chain reaction, as well as elevated specific immunoglobulin M antibodies against it, established the diagnosis. Characteristic brain lesions on magnetic resonance imaging were absent and ophthalmologic examination revealed no inflammatory lesions in the retina and choroid. Treatment with pyrimethamine, sulfadiazine, and folic acid resulted in a complete recovery after 2 months of therapy
Jimenez-Caballero 2010	Case of ADEM in a 76-yr-old immunocompetent male (HIV negative) from Spain; who presented with a 7 ds Hx of fever, malaise, inability to walk, and drowsiness//ALOC, Stupor, nystagmus, dysarthria, R dysmetria, ataxia, global hypereflexia//Toxoplasma IgG and IgM were positive //CSF analysis showed hypoglycorrhachia (CSF Gl = 50/serum 93 mg/dl) and elevated CSF protein, but without CSF pleocytosis///Brain MRI showed multiple Hyperintense T2 lesions//FLAIR images showed pseudonodules with vascular distribution//Given presumed diagnosis of ADEM after acute Toxoplasma infection, patient was initiated on Steroids;(5 ds with a 15 ds tapering with good clinical response and progressively there was recovery in the LOC, hemiparesis, ataxia and dysarthria///At 3 mo f/up he was asymptomatic and brain MRI at 6 mo F/up showed resolution of the brain lesions (at that time there was significant rise in *Toxoplasma* IgG and IgM had become negative)
Nunura 2010	Pneumonia, retinochoroiditis, hepatitis, synovitis and myositis//in a 37-yr-old immunocompetent male (HIV negative) marine on a military operation by the Peruvian Amazon x prior 4 mo///who presented with R hip pain and limping; subsequently on HD 5 developed Fever, Dyspnea, tachypnea, rales in the left hemithorax and decreased Visual acuity//Also had leukocytosis, transaminitis, CXR with diffuse interstitial infiltrates////Chest CT with multiple nodular cavities and pleural effusions//Eye findings suggestive of *Toxoplasma* chorioretinitis//On HD15 results of *Toxoplasma* serology showed positive *Toxoplasma* IgG, IgM, a thick blood smear was positive for tachyzoites//Initially was started on Clarithromycin + TMP/SMX+ Clindamycin +CTX (but on HD5 was changed to Vanco + Imipenem and his fever/cough and dyspnea persisted//After the *Toxoplasma* serology results had become available was started on Pyrimethamine + Sulfadoxine (Clindamycin + TMP/SMX were discontinued) and patient defervested on the 2nd d of this treatment //R hip MRI showed avascular necrosis and mild synovial effusion and R hip muscle biopsy showed T. gondii bradyzoites//Patient 1 year later had only achieved partial recovery from his chorioretinitis and hip disability//*Risk factors: Cooked for the brigade, brigade frequently hunted mammals//drank stream water and ate undercooked meat and had 3 cats)*
Pergola 2010	Acute pericarditis and myocarditis//in a 32-yr-old immunocompetent man (HIV negative) living in France who presented with 2 wk Hx of severe chest pain worsening with deep breathing, fever, arthralgia, malaise, LADP//Had elevated cardiac enzymes (troponin, CPK), high LDH, elevated inflammatory markers//ECHO with moderate pericardial effusion, hyperechoic pericardium slightly detached in the back side// *Toxoplasma* IgM was positive; with further increase in *Toxoplasma* IgM titers within the next 10 ds (*Toxoplasma* IgG still negative at that time; but became *Toxoplasma* IgG positive 10 weeks after D/C)//Patient’s condition clinically improved and patient was discharged on HD12, with resolution of ECHO findings (resolution of pericardial effusion) ///Patient received treatment with Spiramycin x 1 month; in addition to anti-inflammatory medications//
Simanaityte 2010	Toxoplasmic pneumonia with small left pleural effusion.//in a 29-yr-old immunocompetent man (HIV negative) (most likely from France); who presented with a 4 ds Hx of malaise, high fevers, night sweats//subsequently developed headache without meningeal signs; weight loss, generalized LADP; Hypoxia (92% O2 Sats); elevated inflammatory markers, high LDH, //Chest CT showed left lobar pneumonia with small pleural effusion//Given the mononucleosis like syndrome presentation and the LADP *Toxoplasma* serology was sent; with positive *Toxoplasma* IgG and IgM and low avidity//Patient initially received Roxithromycin, and defervested after 4 ds; and subsequently changed to Spiramycin (x 3 weeks total)//2 weeks after D/C patient remaining afebrile, although malaise persisted//
Alapatt 2009	Cerebral toxoplasmosis in a 40-yr-old immunocompetent pregnant woman (HIV negative) from India who presented with a 4 mo Hx of behavioral changes and generalized seizures (during the 5th mo of pregnancy); 3 mo Hx of headaches, left sided weakness and spastic hemiparesis diplopia; with subsequent development of unsteady gait; urine and stool incontinence; confusion and dysphasia; Upper motor neuron palsy of the facial nerve; increased DTRs and positive Babinski on the Right side.//Brain MRI revealed large Hyperintense lesion in the R basal ganglia, periventricular grey matter, left frontal and parietal midbrain and pons; with hydrocephalus AND mass effect; with bulging of the mass on the R wall of the 3d ventricle (initially concerning for glioblastoma)//Underwent brain mass biopsy and ventriculostomy with improvement of the level of consciousness//Delivered vaginally///Histopathology revealed inflammatory pathology, most likely toxoplasmosis/ Serum *Toxoplasma* IgG and IgM were positive//Started P/S x 6 weeks//Repeat Head CT showed marked decrease in the size of the lesion//Continued anti-Toxoplasma treatment x 6 months and remained clinically well without headache, or confusion; Her Left hemiparesis had not fully recovered and remained on anticonvulsants///
Habek 2009	Toxoplasmic encephalitis, rapidly progressing dementia///69 yr old immunocompetent HIV negative male from Croatia, with a 2 mo hx of rapidly progressive cognitive decline with poor speech, dyslexia, dysgraphia, constructional apraxia, verbal and visual memory deficits, positive Babinski and left side and gait apraxia//Brain MRI T2 images showed Hyperintense lesions in both thalami and putamina and head of caudate nucleus without diffusion restriction on DWI sequences (T2 shine through) ///CSF showed elevated protein (1.08 g/L) without pleocytosis and elevated CRP//Clinical course complicated by gram negative sepsis and patient died, with toxoplasmic encephalitis revealed at the autopsy///Brain autopsy showed confluent small and soft partially hemorrhagic lesions in basal ganglia with few cysts containing *T.gondii* bradyzoites *(per authors conclusion: Toxoplasmic encephalitis should be considered in the differential diagnosis as a rare cause of rapidly progressive dementia in immunocompetent patients. Moreover, in the presence of multiple hyperintense lesions on T2 and hypointense lesions on T1 images without diffusion restriction, toxoplasmosis should also be considered even in the absence of CSF pleocytosis, particularly so though in the presence of elevated CSF protein)*
Pino Salinas 2009	23-yr-old male from Colombia Armed Forces, admitted to ICU with Respiratory failure, NYHAIII, myalgias, arthralgias, HA, LADP////ABSTRACT: Toxoplasmosis is a common opportunistic infection in patients infected with HIV/AIDS while in immunocompetent patients this infection causes symptoms only in 10% to 20% of the cases, generally with a benign and autoresolutive course. In the last decade some severe cases with visceral involvement has been reported in immunocompetent patients, though they were isolated or recovered during years. //We present the first Colombian report of an epidemic outbreak caused by Toxoplasma gondii in military personnel deployed to rural areas located at La Macarena, Meta, Colombia. All 18 cases were confirmed by IgG indirect immunofluorescence (IFI) with titers higher than 1:1024 (normal range: 1:16). Their main symptoms were fever, adenopathies and pulmonary and gastrointestinal compromise. One patient developed severe myocardial compromise. All the patients recovered after treatment with pyrimethamine/sulfadoxine and clindamycin for 3 weeks. A possible hypothesis for this out- break was the consumption of contaminated water with oocysts, and probably the severity of the compromise could be elicited by a “wild” strain of the parasite as it is reported in the literature. Unfortunately, it was impossible to isolate and identify the specific strain.
Pino Salinas 2009	31-yr-old male from Colombia Armed forces, who presented with dyspnea NYHA III, HA, myalgias, sweating, LADP//ABSTRACT: Toxoplasmosis is a common opportunistic infection in patients infected with HIV/AIDS while in immunocompetent patients this infection causes symptoms only in 10% to 20% of the cases, generally with a benign and autoresolutive course. In the last decade some severe cases with visceral involvement has been reported in immunocompetent patients, though they were isolated or recovered during years. //We present the first Colombian report of an epidemic outbreak caused by Toxoplasma gondii in military personnel deployed to rural areas located at La Macarena, Meta, Colombia. All 18 cases were confirmed by IgG indirect immunofluorescence (IFI) with titers higher than 1:1024 (normal range: 1:16). Their main symptoms were fever, adenopathies and pulmonary and gastrointestinal compromise. One patient developed severe myocardial compromise. All the patients recovered after treatment with pyrimethamine/sulfadoxine and clindamycin for 3 weeks. A possible hypothesis for this out- break was the consumption of contaminated water with oocysts, and probably the severity of the compromise could be elicited by a “wild” strain of the parasite as it is reported in the literature. Unfortunately, it was impossible to isolate and identify the specific strain.
Pino Salinas 2009	24-yr-old male from Colombia Armed Forces, admitted to ICU with respiratory failure, cough, dyspnea NYHA IV, HA, malaise, LADO///ABSTRACT: Toxoplasmosis is a common opportunistic infection in patients infected with HIV/AIDS while in immunocompetent patients this infection causes symptoms only in 10% to 20% of the cases, generally with a benign and autoresolutive course. In the last decade some severe cases with visceral involvement has been reported in immunocompetent patients, though they were isolated or recovered during years. //We present the first Colombian report of an epidemic outbreak caused by Toxoplasma gondii in military personnel deployed to rural areas located at La Macarena, Meta, Colombia. All 18 cases were confirmed by IgG indirect immunofluorescence (IFI) with titers higher than 1:1024 (normal range: 1:16). Their main symptoms were fever, adenopathies and pulmonary and gastrointestinal compromise. One patient developed severe myocardial compromise. All the patients recovered after treatment with pyrimethamine/sulfadoxine and clindamycin for 3 weeks. A possible hypothesis for this out- break was the consumption of contaminated water with oocysts, and probably the severity of the compromise could be elicited by a “wild” strain of the parasite as it is reported in the literature. Unfortunately, it was impossible to isolate and identify the specific strain.
Pino Salinas 2009	21-yr-old male from Colombia Armed Forces, who presented w cough/dyspnea, NYHAII, HA, malaise, LADP///ABSTRACT: Toxoplasmosis is a common opportunistic infection in patients infected with HIV/AIDS while in immunocompetent patients this infection causes symptoms only in 10% to 20% of the cases, generally with a benign and autoresolutive course. In the last decade some severe cases with visceral involvement has been reported in immunocompetent patients, though they were isolated or recovered during years. //We present the first Colombian report of an epidemic outbreak caused by Toxoplasma gondii in military personnel deployed to rural areas located at La Macarena, Meta, Colombia. All 18 cases were confirmed by IgG indirect immunofluorescence (IFI) with titers higher than 1:1024 (normal range: 1:16). Their main symptoms were fever, adenopathies and pulmonary and gastrointestinal compromise. One patient developed severe myocardial compromise. All the patients recovered after treatment with pyrimethamine/sulfadoxine and clindamycin for 3 weeks. A possible hypothesis for this out- break was the consumption of contaminated water with oocysts, and probably the severity of the compromise could be elicited by a “wild” strain of the parasite as it is reported in the literature. Unfortunately, it was impossible to isolate and identify the specific strain.
Pino Salinas 2009	31-yr-old male from Colombia Armed Forces who presented w cough/dyspnea, NYHA III, LADP, HA, myalgia and a 21-yr old with cough/dyspnea, NYHA II, HA, malaise ///ABSTRACT: Toxoplasmosis is a common opportunistic infection in patients infected with HIV/AIDS while in immunocompetent patients this infection causes symptoms only in 10% to 20% of the cases, generally with a benign and autoresolutive course. In the last decade some severe cases with visceral involvement has been reported in immunocompetent patients, though they were isolated or recovered during years. //We present the first Colombian report of an epidemic outbreak caused by Toxoplasma gondii in military personnel deployed to rural areas located at La Macarena, Meta, Colombia. All 18 cases were confirmed by IgG indirect immunofluorescence (IFI) with titers higher than 1:1024 (normal range: 1:16). Their main symptoms were fever, adenopathies and pulmonary and gastrointestinal compromise. One patient developed severe myocardial compromise. All the patients recovered after treatment with pyrimethamine/sulfadoxine and clindamycin for 3 weeks. A possible hypothesis for this out- break was the consumption of contaminated water with oocysts, and probably the severity of the compromise could be elicited by a “wild” strain of the parasite as it is reported in the literature. Unfortunately, it was impossible to isolate and identify the specific strain.
Gupta 2008	Toxoplasma granuloma of the brainstem in a 27-yr-old immunocompetent male (HIV negative) from India with sudden onset of headache, left sided weakness and vomiting; with a similar prior episode 1 mo earlier that had self-resolved//Had anisocoria, crossed hemiplegia with left hemiparesis and Righ V, VII, Xi, and X cranial nerves involvement//Brain MRI showed on T1 images a hypotense pontine lesion with extension to midbrain; with heterogeneous contrast enhancement and vague ring enhancement//brain biopsy (due to concern for neoplasm) showed an inflammatory lesion (with dense microglia reaction mixed with foamy histiocytes; focal areas of active necrosis; with foci of spotty calcifications along the edge of the necrotic zone, along with few ruptured but calcified *Toxoplasma* cysts characteristic of Toxoplasmosis (Toxoplasma granuloma of the brainstem) (Risk factors: Prior Hx of owing a cat)//(Serial *Toxoplasma* IgG and IgM were within normal range)//Based on the histopathologic findings was started on P/S x4 wks //At 6 mo f/up had complete recovery with no residual neurologic deficits; with repeat brain MRI showing complete resolution of brain lesion//At 2 yrs f/up remained asymptomatic
Rostoff et al. 2008	Myopericarditis with acute heart failure (NYHA IV), large pericardial effusion and B/L pleural effusions in a 67 y F, who presented w severe dyspnea at rest, left pleuritic chest pain, high fever, generalized LADP (w a PMH of suspected myocarditis 3 mo PTA with LVEF 35% but w normal troponin)//w CXR showing B/L pleural effusions and cardiomegaly//w ECHO showing LV systolic dysfunction with LVEF 33% and large pericardial effusion//w Chest CT confirming the presence of pericardial and pleural effusions//w markedly elevated inflammatory markers (CPR 232 mg/dl; ESR 100 mm/h); with slightly high Troponin (0.10–1.12 ng/dl)//With significant clinical improvement of cardiac symptoms after 3 wks of Spiramycin treatment (Spiramycin was used because of sulfa allergy); with normalization of inflammatory markers; resolution of CXR and ECHO findings with increase in LVEF to 50%//Patient was discharged after 7 wks of treatment with no further evidence of Heart Failure///Also with elevated serum CA-125 during acute presentation and normal CA-125 at 1 year follow up
Demar 2007	The hospitalized patients, who did not have any immunodeficiencies, presented with an infectious disease with multivisceral involvement. Serological examination confirmed acute toxoplasmosis. One adult died, and a neonate and a fetus with congenital toxoplasmosis also died. During the investigation, 4 additional acute cases of toxoplasmosis were diagnosed among the 33 villagers. Only 3 inhabitants had serological evidence of previous *T. gondii* infection. In total, we reported 11 cases of toxoplasmosis: 8 multivisceral cases in immunocompetent adults, resulting in 1 death; 2 cases of lethal congenital toxoplasmosis in a neonate and a fetus; and 1 symptomatic case in a child. Molecular analysis demonstrated that identical isolates of only 1 atypical strain were responsible for at least 5 of the 11 cases of toxoplasmosis in the outbreak. No epidemiological sources could be linked to this severe community-wide outbreak of toxoplasmosis.
Leal 2007	41-yr-old immunocompetent male (HIV negative) from Brazil w 8 ds of fever, myalgias, HA; this occurred 20 ds after eating semiraw beef 20 ds earlier,//Progressed to Respiratory failure w B/L interstitial infiltrates///*Toxoplasma* IgM+, Positive *Toxoplasma* PCR from CSF, nl CSF count/ positive mice subinoculation/ w marked clinical radiographic and laboratory improvement after 4 ds of anti- *Toxoplasma* treatment Pyrimethamine/Sulfadiazine/ Steroids (after treatment CD4 increased from 435 to 921 /mm3)//ABSTRACT: Pulmonary toxoplasmosis is rare in immunocompetent subjects. Here, we describe a 41-year-old previously healthy male patient who presented to the emergency department of a hospital with a life-threatening case of pneumonia due to Toxoplasma gondii infection, which responded to specific therapy. Clinical and image-based findings overlap with those for atypical pneumonias, and toxoplasmosis should be considered in the differential diagnosis—especially if immunoglobulin M–specific antibodies are detected.
Mariani 2006	ABSTRACT: We report a case of toxoplasma myocarditis in a young healthy man.///Patient had 2 Toxoplasmosis manifestations: myocardial involvement with complete AV block and cerebral lesions. This patient is the second AV block case related to toxoplasma infection described in the literature.
De Salvador 2005	ABSTRACT: We report a case of severe acute primary pulmonary toxoplasmosis in an immunocompetent young man living in Nice (Southern France). The Toxoplasma DNA extracted from the broncho-alveolar lavage fluid allowed a genetic characterization of the responsible strain which displayed an atypical genotype of Toxoplasma gondii. This unusual genetic composition of the parasite may have influenced, among other factors, the severity of the disease.///The 11-yr-old sister of the patient also developed Acute toxoplasmosis with fever and LADP, but without pulmonary findings//Thus for the severity of the presentation the atypical implicated *T.gondii* genotype might not be solely responsible. //A massive parasite load in the 19-yr-old pt might have overwhelmed the immune response and led to a severe toxoplasmosis. //In such cluster cases differences in inoculum size are more likely explained by ingestion of oocysts rather than ingestions of cysts in infected meat
	
Kaushik 2005	Meningoencephalitis//in a 28-yr-old immunocompetent F from India (HIV negative x2) with 2 mo Hx of low grade fever, Headache and vomiting; with worsening headache and deterioration of level of consciousness, confusion, irritability, neck rigidity and hepatomegaly//Spinal tap showed elevated opening pressure; Pleocytosis (495 cells/mm3); elevated CSF protein 210 mg/dl(unl < 50 mg/dl) and low CSF glucose (24 mg/dl)//Head CT showed multiple ring lesions with hypointensive centre and moderate hydrocephalus, with meningeal enhancement ///T2 weighted images showed moderate dilatation of the lateral, third and fourth ventricles with periventricular hyperintensity, and multiple widely scattered ring lesions with a hypointense centre and a peripheral hyperintense rim in both the cerebral hemispheres including the thalamus and basal ganglia. Lesions were also visualized in the pons, middle cerebellar peduncles and both the cerebellar hemispheres. The majority of these lesions was associated with perifocal edema//Patient was initially started on anti-TB therapy+ steroids for suspected TB meningoencephalitis with multiple tuberculomas)///After 10 ds Brain MRI T1 images showed isointense to Hyperintense exudate in the supra-cellar cistern///Repeat CSF analysis showed decrease in pleocytosis (from 495 to 250 cells/mm3; probably due to the Steroid treatment); increase in CSF protein (From 210 to 440 mg/dl) and increase in CSF glucose (From 24 to 60 mg/dl); probably due to steroids//Failure to respond to anti-TB treatment led to the suspicion of Toxoplasmosis///Toxoplasma IgG was high positive (1294 IU.ml; cutoff < 15 IU/mL) and positive *Toxoplasma* IgM (1.48; cutoff <1.1 AI)//Patient was started on TMP/SMX x 6 weeks; with rapid initiation of clinical improvement within 3 ds of TMP/SMX; with gradual disappearance of fever and headache over 2–3 weeks///Repeat spinal tap after 3 weeks of anti- *Toxoplasma* treatment showed normalization of opening pressure and CSF cell country//Repeat brain MRI after 6 wks showed regression in size of all lesions; Some of these lesions appeared as foci of hypointense signals on the T2 weighted images, which accentuated on the T2 images, consistent with calcification. ///(Absence of LADP make this presentation rare)
Bossi 2002	Disseminated toxoplasmosis,//30-yr-old immunocompetent (HIV negative) man from French Guiana, who presented with a 3 wk Hx of fever, cough, rash, myalgia, myositis, diffuse LADP, HA, confusion (Risk eating undercooked boar meat); with with b/l interstitial infiltrates and b/l pleural and pericardial effusions, HSM, transaminitis and pancreatitis//Chest CT bilateral interstitial infiltrates with b/l pleural and pericardial effusions//Once ABX switched to Spiramycin, his respiratory symptoms and pericarditis improved within 5 ds//CD4 = 332// *Toxoplasma* IgG and IgM highly positive titers//Muscle Biopsy showed *T.gondii* cysts and tachyzoites// treated with Pyrimethamine + Sulfadiazine (x 6 wks) and condition improved; defervested within 5 ds and muscle symptoms resolved within 15 ds//F/up 1 year later was asymptomatic.
Vastava 2002	*Toxoplasma* encephalitis//in a 22-yr-old immunocompetent man (HIV negative) from India, who presented with fever, HA, left sided weakness and gradual LOC (loss of consciousness), with Left hemiparesis//CSF with mild pleocytosis and high protein (112 mg/dl)//High *Toxoplasma* IgG in blood and CSF//Brain MRI: w large hemorrhagic lesion in R parietal region with perifocal edema and mass effect//Multiple lesions with High T2 signal, with multiple nodular and linear enhancing lesions//Started Pyrimethamine + Sulfadiazine w marked clinical improvement within 2 weeks; f/up MRI 4 wks later showed regression of the lesions//6 mo f/up MRI with almost complete regression of the lesions//
Vastava 2002	*Toxoplasma* encephalitis//in a 35-yr-old immunocompetent man (HIV negative) from India, with a 15 ds Hx of ALOC; who presented with severe throbbing headache, sudden onset of vomiting and low grade fever//Brain MRI showed multiple high T2 signal lesions in both hemispheres, with linear radiation and nodular contrast enhancement //Started empirically Pyrimethamine + Sulfadiazine, with marked clinical improvement within 1 week//f/up MRI after 2 weeks showed regression of the lesions and 6 mo f/up MRI showed only small residual lesions with no contrast enhancement
Paspalaki 2001	Polymyositis and myocarditis (severe proximal muscle weakness, elevated muscle enzymes, positive EMG consistent with subacute inflammatory myopathy; ECHO: dilation of Left ventricle w poor contractility suggestive of myocardial involvement)//////(13-yr-old immunocompetent girl with 3 wk hx of high fevers, malaise, mild proximal muscle weakness, weight loos, and vomiting; transaminitis, and a round 2 cm non cystic liver lesion by abdominal US and CT//initially presumed to have bacterial liver abscess; with rapid neurologic deterioration with dysphagia, inability to walk and raise arms, further weight loss, fine tremor of hands, maculopapular rash on palms and soles; with EMG consistent with subacute inflammatory myopathy//with ECHO showing left ventricle dilation with poor contractility///With rising and eventually very high *Toxoplasma* IgG titers (up to 1:109,350 4 weeks later prior to any Tx/steroids) and positive *Toxoplasma* IgM)//Had favorable response to steroids only treatment; with resolution of fever within 2 wks; and improvement of clinical and laboratory findings; with resolution of rash on palms and resolved within 3 months//Authors’ presumed diagnosis with Toxoplasmic acute myositis and myocarditis; the liver lesion might also be attributed to Toxoplasmosis//The improvement with prednisolone along was suggestive of immunologic disturbances contributing to the development of inflammatory myositis//With patient remaining completely asymptomatic at the 4 yr f/up)
Silva 2001	46-yr-old immunocompetent woman (HIV negative) from Brazil; presented with fainting and decreased visual acuity x 8 ds, and inability to perform usual tasks as housewife (with hx of partial seizures due to intracranial calcifications secondary to neruocysticercosis)//with Paresis of Left upper limb; Head CT showed hypodense lesion with vasogenic edema in the R occipital lobe, with heterogeneous contrast uptake around the lesion//MRI revealed an occipital lobe mass measuring 3.5 × 2.9 × 3 cm with perilesional edema and areas of high signal on T1 and T2//Underwent neurosurgical exploration due to concern for CNS neoplasm; and complete surgical excision of two nodular lesions with a yellowish-brown appearance with areas of central necrosis and cavitation; revealing chronic inflammation surrounding the necrotic material; with pseudocysts filled with bradyzoites seen in immunostaining for *T.gondii*//*Toxoplasma* IgG was positive but negative *Toxoplasma* IgM// diagnosis of Brain abscess due to Toxoplasmosis was made ///it was not possible to define whether there was a RLI triggered by an unidentified immunosuppressive factor, or an API with negative IgM in the serum// treatment with P/S was initiated with progressive improvement of initial neurological s/s//At f/up 6 months after diagnosis (without any treatment) reported only hypoesthesia in the feet and NO evidence of immunosuppression 1 year after diagnosis
Sugane 2001	Meningoencephalitis (preceding headache x 2 wks ( associated with high fever and urinary retention)//subsequently facial numbness, gait disturbances progressing to extreme unsteadiness, delirium, positive Kernig’s sign ABSTRACT: We report herein a rare case of *Toxoplasma gondii* meningoencephalitis in a non-AIDS patient. Although T. gondii itself was not detected in nucleated cells in peripheral blood and cerebrospinal fluid under the microscope, the polymerase chain reaction method effectively detected the B1 gene of T. gondii in the cells. A serological examination showed increased levels of the IgG but not the IgM antibody to T. gondii, suggesting reactivation of the infection in the brain.
Chandenier 2000	Acute myocarditis with rapid onset of CHF (congestive heart failure) shortly after acute infection//in a 21-yr-old immunocompetent woman (HIV negative) living in France, who was admitted in the Cardiac ICU with chest pain with deep inspirations, fever and dyspnea.//ECHO showed global hypokinesia with SF 17% (nl range 30–35%) and EF 36% (nl range 60%) but no pericardial effusion; High CPK, LDH, transaminitis, elevated inflammatory markers//Serial *Toxoplasma* serology was positive for *Toxoplasma* IgG, IgM and IgA, confirming recent seroconversion (rising *Toxoplasma* IgG titers))///CHF and myocardial contractility self-normalized within 8 ds, NO anti- *Toxoplasma* treatment was given; (in HD12 suffered bilateral Pulmonary Emboli and recovered ONLY with anti-coagulation treatment)
Chandenier 2000	Cardiac dysfunction, dyspnea, pulmonary edema, transaminitis//in a 9-yr-old immunocompetent girl (HIV negative) living in France, who presented with Acute Pulmonary Edema, Dyspnea and Tachycardia, with 1 wk prior having had fever and dyspnea for which treated with Amoxicillin (fever subsided by dyspnea worsened)//CXR showed Pulmonary edema and cardiomegaly //ECHO showed systolic Left ventricular dysfunction with akinetic basal and median septum and hypokinesia of other ventricular walls//SF 10% (nl 30–35%) and EF 35% (nl 60%)//Cardiac enzymes were normal (CPK, LDH, troponin and Myoglobin), transaminitis//Her clinical condition improved on symptomatic treatment //*Toxoplasma* serology showed positive *Toxoplasma* IgG, IgM and IgA, Blood *Toxoplasma* PCR was negative, but *Toxoplasma* IgG avidity and Agglutination test implied an infection acquired 6–12 mo prior to testing//(*Risk factor: contact with kittens 3 mo prior*) //Pyrimethamine + Sulfadiazine given later (x 4 wks) led to improvement in cardiac function//(at 6 mo f/up the cardiac function was completely normal)
Sanchez 2000	Toxoplasmic encephalitis //in a 61-yr-old immunocompetent male from Recurrent seizures within an 8 mo period (2 episodes 8 mo prior and 1 current episode); multiple nodular brain calcifications along the convexity, paracortical regions and brain base; otherwise normal CSF analysis (normal cells, protein and glucose), +*Toxoplasma* IgG, +IgM, +IgA but high avidity at the time of the 3d seizure episode.///Seizures resolved -with not recurrence over subsequent 6 months-without specific anti- *Toxoplasma* treatment.
Sano 2000	ABSTRACT: There have been several case reports, a total of 22 up to the present, of *toxoplasma* pericarditis. Out of them, in only a few cases the diagnosis was properly made with a proof of the microscopic presence of *Toxoplasma gondii*. This is the first report of *toxoplasma* pericarditis in which the presence of *Toxoplasma gondii* was detected by polymerase chain reaction of pericardial effusion. In addition, the previous reports will be reviewed, and compared to this present case. A 29 yr-old woman, without immunosuppressant disorder, suffering from fever and orthopnea was admitted to our hospital. Blood chemistry findings indicated mild liver dysfunction and inflammation. Chest radiography showed cardiac enlargement. Electrocardiography showed sinus tachycardia and ST elevation. Echocardiography revealed a massive pericardial effusion. Pericardiocentesis demonstrated 638 mL of bloody fluid. Cytologic study of the fluid was class II for malignancy, and polymerase chain reaction to tuberculosis was negative. However, a high titer of the anti- *toxoplasma* antibody of 1: 20,480 (passive hemagglutination) indicated pericarditis caused by *Toxoplasma gondii*. Subsequently, *Toxoplasma gondii* was identified in the pericardial effusion by polymerase chain reaction. Clinical symptoms improved after pericardiocentesis, but 2 months later pericarditis recurred. Treatment was started with 800 mg acetylspiramycin daily but failed to improve the symptoms. Because of the development of pleuritis, treatment was changed to sulfadoxine 1000 mg/pyrimethamine 50 mg. After the treatment with them, her symptoms improved. Only 22 cases of *toxoplasma* pericarditis have been reported worldwide and 15 of those cases were without immunosuppressant disorder. The usual symptoms at the onset of pericarditis without immunosuppressant disorder are fever, dyspnea and chest pain. Seven patients developed cardiac tamponade. Pericardiocentesis was performed in 8 cases and the pericardial fluid was hemorrhagic in 6. Pericardial thickening was detected in 5 cases. The diagnosis of *toxoplasma* infection is very difficult, because asymptomatic infection of *Toxoplasma gondii* is very common. Pericarditis is a disease difficult to confirm the etiology. Detection of *Toxoplasma gondii* in pericardial effusion by the polymerase chain reaction is very useful for its diagnosis.
Calore 2000	ABSTRACT: Skeletal muscle can be the site of inflammatory diseases that lead to muscle weakness, pain, and increased myogenic serum enzymes. Most of these inflammatory myopathies are idiopathic. In some cases, inflammatory myopathies are due to infectious agents. We describe the pathological aspects of muscle biopsies of 2 Brazilian siblings who acquired toxoplasmosis at the same time and in similar conditions. One developed a tetraplegia that was confirmed to be due to inflammatory myositis due to *toxoplasma*. The other developed myocarditis, with heart failure, without skeletal muscle weakness. In both cases many *toxoplasma* organisms were observed in the muscle biopsies, but in case 1 only was there an inflammatory myopathy with myofiber necrosis; the inflammatory cells were predominantly macrophages with some CD4+ cells and rare CD20+ cells. In case 1, expression of CD54 was observed in many inflammatory cells as well in endothelial cells, but only in endothelial cells in case 2. After treatment with clindamycin and corticosteroids both cases had only partial improvement, case 1 with a residual muscle weakness and case 2 with residual cardiac insufficiency (requiring digoxin). These cases show that the presence of the parasite in myofibers is not enough to induce an inflammatory myositis with muscle cell necrosis. This suggests that immunological disturbances may contribute to the development of inflammatory myositis due to *toxoplasma*.
Bossi 1998	ABSTRACT: Cases of severe toxoplasmosis have been reported involving immunocompetent patients living in French Guyana, where oocysts are found in river water and wild animals (2). A poor host adaptation to the uncommon highly virulent tropical strains of T. gondii can explain these unusual clinical presentations. In our case, Guillain-Barre’ syndrome was observed in an immunocompetent patient during a disseminated infection with a new strain of T. gondii. This strain was highly virulent, as confirmed by the rapid death of the mice (within 3 days). Moreover, this strain was not altered by a 10-day treatment with spiramycin (which is ineffective in toxoplasmosis with central nervous system symptoms), and parasitemia remained after this therapy. The combination of Guillain-Barre´ syndrome, massive alteration of the patient’s condition, and skin, pulmonary, liver, pancreatic, and ocular involvement is unique for toxoplasmosis. Only the standard treatment for toxoplasmosis was effective, whereas spiramycin and intravenous IGs were ineffective.//His symptoms started 2 mo PTA= 15 ds after eating undercooked meat of warthog and doe during a 3 ds tour in the tropical forest of French Guyana (fever, HA, maculopapular rash, dry cough, diarrhea//1 mo later admitted with high fever 15 Kg weight loss)
Darde 1998	35-yr-old immunocompetent (HIV negative) military man, presented with malaise, fever, chills, myalgias, HD, B/ conjunctivitis, maculopapular rash and leukopenia after a 4 mo stay in deep forest of French Guyana, HD13 developed LADP, transaminitis, HD15 diarrhea and rales in both lungs//Numerous *T.gondii* trophozoites detected in BAL//Mice subinoculation positive, High Positive *Toxoplasma* IgG, IgM IgA, IgE//transiently low CD4 count = 344/μL//During same period 4 other military personnel returning from French Guyan became sick; ABSTRACT: Disseminated toxoplasmosis with lung involvement is rare in immunocompetent patients. We describe a case with unusual epidemiological aspects associated with a new strain genotype. The unusually severe manifestations in the case we describe could be explained by poor host adaptation to this uncommon parasite strain.
Rosch 1998	Encephalitis, abnormal EEG, hearing loss//in a 9-yr-old immunocompetent boy from Germany who presented with 1week Hx of low-grade fever, headache and clinical signs of encephalitis and unilateral deafness; Anti-*Toxoplasma* treatment with P/S lead to the rapid disappearance of most of the signs and symptoms except for the deafness that persisted (Article in German, only English Abstract)
Iniguez 1997	Miller-Fisher syndrome (ataxia, ophthalmoplegia and areflexia) / in a 30 Yr old immunocompetent patient who presented with Miller-Fisher syndrome with central and peripheral neural involvement, and diplopia with acute Toxoplasmosis
Montoya 1997	Acute toxoplasmosis with skeletal muscle and myocardial involvement and CHF, S/P cardiac arrest and Seizures//ABSTRACT: We report the first case of biopsy-proven toxoplasmic myocarditis and polymyositis simultaneously occurring in the same individual that was diagnosed during life. Results of her toxoplasmic serology were consistent with acute toxoplasmosis. She subsequently developed visual symptoms consistent with toxoplasmic chorioretinitis. She had a positive clinical response to therapeutic agents specific against *Toxoplasma gondii.* Her toxoplasmic serological profile established the diagnosis of acute toxoplasmosis////(1 mo PTA fever and URI symptoms, 2 wks PTA flu vaccination; subsequently developed myalgias, HA, LADP, exertional dyspnea, orthopnea//admitted with acute onset of CHF (EF 30%; hypokinesia, mitral regurgitation, Myocardial Bx: showed myocarditis of unknown etiology)//Initially treated with steroids for presumed viral myocarditis and her symptoms gradually resolved///During steroid tapering developed proximal muscle weakness and Muscle Biopsy showed multiple T. gondii cysts (ECHO at that time EF 60%) Started treatment with Pyrimethamine/Sulfadiazine and Prednisone was reinstituted//////2 mo later developed progressive dyspnea and signs of CHF////Followed by Progressive sinus bradycardia and complete Heart Block associated with seizures and respiratory failure, Hypoxia//required inotropes, CSF was normal, CXR worsening CHF (at that time transferred to Stanford); had hyponatremia, transaminitis, CXR showed B/L interstitial and mild alveolar airspace changes in the RLL and Left retrocardiac region with small b/l effusions (at that time blood PC and mice sub-inocculation were negative)/////subsequently developed visual symptoms consistent with toxo chorioretinitis//She responded to anti-*Toxoplasma* treatment (Pyrimethamine + Sulfadiazine + Steroids initially, then Pyrimethamine + Clindamycin + Steroids)///12 ds prior to onset of Symptoms had eaten raw beef and raw lamb, Had symptoms of myalgia/HA/LADP for 13 ds PTA
Couvreur 1996	ABSTRACT: Objectives: Over a period of 13 years (1982–1995), 49 cases of acquired toxoplasmosis complicated with ocular and/or neurologic or meningeal involvement were observed in our toxoplasmosis laboratory. This series includes 43 cases of isolated ocular lesions, 3 cases of meningoencephalitis (associated with retinochoroiditis in 1 case), 1 case of meningitis with uveitis, 1 case of polyradiculoneuritis and 1 case of facial nerve palsy.Methods: The patients were aged 1 to 62 years. None had either spontaneous or iatrogenic immunodeficiency. There were two steps in the diagnosis. First congenital infection was eliminated on one or several of the following criteria: any possibility of maternal infection during pregnancy ruled out in 26 cases, evidence of recent acquired infection (i.e., clinical and/or serological evidence of recent acquired toxoplasmosis in 17 cases, retinochoroiditis in non-twin siblings in 3 cases). The second step was to confirm the diagnosis of *toxoplasma* infection. Apart from serological evidence of recent infection, confirmation included specific local antibody synthesis in the aqueous humor of the eye and/or in cerebro-spinal fluid or ocular lesions characteristic of toxoplasmosis and absence of other etiology.Results: Ocular lesions were unilateral in 43 cases among 45. A mean follow-up of 37.9 months revealed relapses in 14 among 36 patients (39%). As routine serological examination for toxoplasmosis is compulsory in France since 1978, it was possible to document retrospectively the immune status of the mothers of many of the patients of the present series during pregnancy and to rule out congenital toxoplasmosis in a number of cases. This might explain the discrepancy between the relatively large number of cases in the present series and the fact that complicated acquired toxoplasmosis has been considered hitherto as relatively rare in immunocompetent patients.Conclusion: Based on the epidemiology of ocular toxoplasmosis and the data obtained here, it is suggested that the acquired pattern of ocular toxoplasmosis might be more frequent than estimated up to now.
Duffield 1996	Toxoplasmic myocarditis resulting in recurrent symptomatic AV dissociation in a 25-yr-old immunocompetent patient (HIV Not performed, but was well at 1 yr f/up) presented initially w 10 ds Hx of Fever, Myalgias, lethargy that self-resolved//then 1 wk later acute unwell with hypotension, dizziness, chest tightness, EG showed AV dissociation, normal cardiac enzymes//1 mo later also LADP//Excision of LN showed Toxoplasmosis//Had new episode of Collapse again with AV dissociation, and profound hypotension, ECHO showed impaired LVF, hypotension//Responded to Dual chamber pacing///Positive *Toxoplasma* IgG Dye test and positive *Toxoplasma* IgM///After the 3d recurrence of AV block started Pyrimethamine + Spiramycin and a permanent dual chamber pacemaker was implanted//ABSTRACT: “We believe this case is unique in that Toxoplasma gondii myocarditis resulted in recurrent, symptomatic AV dissociation with interference. During each attack an identical pattern of AV dissociation was present. Resolution of hypotension and symptoms followed AV sequential pacing. The patient was not bradycardic and LV dysfunction occurred only once. Thus, AV dissociation caused hypotension. This pattern suggests that the patient was experiencing a condition similar to pacemaker syndrome, which occurs in some patients with a ventricular demand pacemaker. The severity of the condition is variable, ranging from flushing to hypotensive collapse, and is not always reproducible. There is often a delay of hours or days in the development of symptoms. The condition is thought to be mediated by several mechanisms including the activation of atrial stretch receptors during AV dissociation, causing an inappropriate reduction in peripheral resistance, and retrograde VA conduction. The latter does not occur during AV dissociation with interference. However, VA conduction is not a prerequisite for development of pacemaker syndrome”.
Lesenne 1996	ABSTRACT: Acute myocarditis due to toxoplasmosis infection has been previously reported, usually in patients suffering from immuno-depression. Cardiac involvement by toxoplasmosis is rare in subjects with a normal immunological status. The authors report the case of a 16-yr-old immunocompetent (HIV negative) patient without immuno-depression with acute myocarditis caused by toxoplasmosis simulating myocardial infarction. //Patient’s symptomatology rapidly resolved//Patient had a favorable outcome without complications from the Toxoplasmic myocarditis//Toxoplasmic myocarditis in IC patients has a good prognosis//////(Acute onset of symptoms of chest pain, fever, EKG ST changes, initial diagnosis of myocarditis mimicking acute MI, had LADP, and splenomegaly, elevated CPK and MB fraction//*Toxoplasma* serology initially *Toxoplasma* IgG, IgM and IgA negative with f/up serology showing seroconversion (positive IgG, high positive IgM and IgA) Treated with Spiramycin and f/up ECHO 2 mo later was normal (also the previously abnormal scintigraphy scan was subsequently normalized)
Lescop 1995	Diffuse toxoplasmic encephalitis with rapidly fatal outcome (within HD #6); Brain CT and MRI showing non-specific finding of brain swelling (Figiure 1) and cortical infarcts due to vasculitis; Spine-echo pTw: flatening of the grooves and the periencephalic cisterns; Spine-echo pT1: small haemorrhagic zone in the R parietal cortex, Spin Echo pT1: minimal enhancement after gadolinium injection (Figiure 2)
Micheli 1994	ABSTRACT: We discuss the unusual presentation of acquired toxoplasmosis in a girl with severe and transient hemidystonia as a unique symptom. Serum titers of anti-*toxoplasma* antibodies increased whereas no specific antibody response in the CSF was observed. While symptomatic drugs were inefficacious, specific anti-toxoplasmosis therapy led to complete and permanent recovery within 2 months. ///1 d of fever 4 wks PTA for repetitive contractures of right thumb, with progressive worsening of involuntary movement and within subsequent 20 ds unable to handle objects or write//Toxo IgG serology showed significant rise in titers (CSF toxo IgG was negative)//The neurologic symptoms progressively worsened with athetoid like movements and Ballistic like hyperkinesia on the R//Eye exam, abd Brain CT and Brain MRI were all normal///treatment with symptomatic drugs (benzodiazepines etc) was not effective//When specific anti-*Toxoplasma* treatment with Pyrimethamine + Spiramycin+ Sulphonamides was instituted, the clinical condition progressively improved and within 2 mo the girl completely recovered; while the EEG normalized in 8 mo (of note earlier EEG showed posterior pseudoperiodic synchronous sharp waves during eye closure, 2 later EEG showed no paroxysmal activity excluding epilepsy) (the absence of CSF *Toxoplasma* antibodies supports the hypothesis of focal vasculitis)
Candolfi 1993	Pulmonary Toxoplasmosis (Pneumonia)//in a 33-yr-old immunocompetent (HIV negative) pregnant woman from Cambodia; with a 7 d Hx of dyspnea, fever, dry cough, CXR with reticulondular opacities B/L//BAL showed *T.gondii* by immunoperoxidase staining//*Toxoplasma* IgG and IgM initially negative, but had seroconversion 2 wks later with positive *Toxoplasma* IgG and IgM//Was treated initially with Spiramycin (before the BAL results) and symptoms improved within 1 week; subsequently given Pyrimethamine/Sulfadiazine x 5 wks (until delivery)//*Initially had low CD4 count (CD4: 250) which normalized after anti- Toxoplasma* treatment
Desguerre 1993	Acute respiratory distress with interstitial pneumonia, myocarditis and pericardial effusion and subsequently also meningoencephalitis //in a 2 yr old immunocompetent child (HIV negative) living in Guyana, who presented with respiratory distress, fever; pericardial fluid was drained//Subsequently within 1 mo developed also spastic quadriparesis and chorioretinitis; with emotional liability and limited expressive language; nystagmus//*Toxoplasma* IgG and IgM positive///Treated with Fansidar + Spiramycin but without response///Elevated CSF protein (100mg/dl) without pleocytosis initially, but subsequently developed pleocytosis (CSF WBC 47/mm3,high CSF protein (80 mg/dl) and positive CSF *Toxoplasma* IgG and *Toxoplasma* IgM (this, along with the presentation with acute pneumonia and myocarditis, argues against a case of congenital Toxoplasmosis and favored the diagnosis of acquired toxoplasmosis)//Brain CT: w numerous calcifications and Hypodense areas//Brain MRI with high T2 signal in periventricular white matter, internal capsule, and “centres semi-ovales”//Treated with 6 weeks of P/S (Fansidar) with stabilization of the diseases and then placed in maintenance treatment with weekly Fansidar x 2 years//At 2 years f/up: has persistent asymetric tetraparesis and had 2 seizures and the previously noted MRI white matter increased signals in the periventricular area and internal capsule had persisted but had decreased in intensity
Guerot 1992	Pericarditis//in a 20 yr-old immunocompetent man living in France (HIV negative) who presented with fever and LADP, *Toxoplasma* IgG and IgM were positive///treated with Spiramycin x 8 ds with resolution of fever, that subsequently relapses with onset of new chest pain suggestive of pericarditis, w characteristic EKG findings and cardiomegaly on CXR //ECHO showed a large pericardial effusion without cardiac tamponade///Cardiac symptoms persisted with anti-inflammatory treatment x 10 ds and then Pyrimethamine/Sulfadiazine was given, with rapid resolution of clinical symptoms and pericardial fluid///At 3 months of f/up patient remained well
Lyngberg 1992	Severe exudative pericarditis w cardiac tamponade in a F patient with SLE like musculoskeletal symptoms for the 8 preceding months; with relapsing pericarditis despite 2 anti-*Toxoplasma* treatment courses with Pyrimethamine/Sulfadiazine (x 3 wks and 7 wks respectively)//61-yr-old immunocompetent woman (HIV negative) from Denmark who presented with SLE like symptoms (synovitis R knee, bursitis, finger stiffness and generalized malaise, myalgias, and weight loss (not responding to 8 months treatment with NSAIDS and Sulfasalazine)///Toxoplasmosis reactivation was suspected based on high *Toxoplasma* IgG, *Toxoplasma* IgM Negative// treatment with pyrimethamine/Sulfadiazine given x 3 wks//1 wk after the end of the anti- *Toxoplasma* treatment there was relapse with worsening clinical symptoms, dyspnea, pericarditis symptoms (patient refused further treatment) and pericarditis progressed to cardiac tamponade; Cardiomegaly in CXR and pericarditis EKG findings//ECHO showed severe exudative pericarditis with normal myocardium//Pericardial fluid was *Toxoplasma* POSITIVE (Mice sub-inoculation with pericardial fluid was positive and mice died from Toxoplasmosis)///Pericardiocentesis showed marked leukocytosis// treated with Pyrimethamine/Sulfadiazine + Prednisone (after the cardiac tamponade had already occurred) x 7 weeks and cardiac symptoms gradual resolved////2 months after the 7 wks anti-*Toxoplasma* treatment there was another relapse of Symptoms that was treated again with anti-*Toxoplasma* treatment x 4 wks of P/S and clinical condition improved again with resolution of the SLE like symptoms and decrease in the ANA//Patient remained well at 9 months follow up. (diagnosis *in patients with Rheumatic mimicking disease may be delayed, and false positive Toxoplasma IgG and IgM results in patients with ANA antibodies have been described. Patients with SLE should be screened for Toxoplasmosis)*
Shimoni 1989	Pneumonitis with pulmonary infiltrate in right upper lobe//in a 51-yr-old immunocompetent man (HIV negative) living in Israel with inactive RA + (treated only with indomethacin, without any steroids or immunosuppressive medications), who presented with a 2 wk Hx of generalized LADP and RUL pneumonia, not responding to Amoxicillin//*Toxoplasma* Dye test positive and *Toxoplasma* IgM positive//His general condition otherwise was excellent//Was D/C home on TMP/SMX and at 2 mo f/up was clinically asymptomatic, with resolution of CXR findings//At 1 yr f/up patient continued to remain healthy
Rafiqul Islam 1989	FUO (low grade Fever 100–1011) and malaise x 5 months and lymphadenopathy///(in a 17-yr-old immunocompetent male from Bangladesh//with 5 months of FUO, malaise and generalized LADP, thrombocytopenia //Strongly positive Latex Agglutination test for Toxoplasmosis (no known risk factors for toxoplasmosis)//LN biopsy suggestive of toxoplasmic lymphadenitis//With Response to anti-*Toxoplasma* treatment (P/S x4 wks) with resolution of fever and disappearance of LADP)//(not known risk factors for toxoplasmosis reported)
Oseroff 1988	Fever, polymyositis, myocarditis, hepatitis, nephrotic syndrome and transaminitis//in a 48-yr-old woman with high fever (that lasted x 6 weeks), dyspnea, generalized myalgias, muscle weakness, N/V/D and pitting edema in extremities; with leukocytosis (WBC = 35K); eosinophilia (548--> 1500/mm3), transaminitis, Elevated CPK, with high MB fraction, elevated LDH and elevated inflammatory markers (ESR = 70 mmHg)///Subsequently developed non cardiogenic pulmonary edema (with large proteinuria) requiring IMV//Fever persisted x 6 weeks; Chest CT and Gallium scan were normal////*Toxoplasma* serology showed rising *Toxoplasma* IgG titers over a 3 month period//By the time the Toxoplasmosis diagnosis was made, patient was already clinically improving with just supportive care and NO anti-*Toxoplasma* treatment //No anti-*Toxoplasma* treatment was given//At 2 mo after D/C clinically asymptomatic
McCabe, Remington 1987	Transient R lung pneumonia with pleural effusion at the onset of the illness; with persistence of fever, LAPD and HSM for 7 months///in a 32 yr-old woman, missionary in the jungles of Venezuela, who presented with fevers, chills, malaise, myalgias, headaches and anorexia not responding to malaria treatment ///generalized LADP and HSM present//Transient R pneumonia with pleural effusion detected///ON HD45 subinocculation to mice of LN biopsy tissue was positive for Toxoplasmosis //*Toxoplasma* serology consistent with acute acquired Toxoplasmosis infection with high positive IgG and IgM //5 family members were also *Toxoplasma* positive
Cottrell 1986	Encephalitis//in a 4-yr-old immunocompetent boy, living in England, with a 3 week Hx of recurrent generalized seizures, increasing in frequency and ataxia, tremor, dysarthria//Brain CT and CSF were normal; EEG showed seizure activity/ *Toxoplasma* IgG was positive and “subsequently” *Toxoplasma* IgM also became positive//Before the Toxoplasmosis results were available, child was started on Steroids and Phenobarbital with dramatic decrease in seizures and ataxia within 3 ds////At steroid tapering there was recurrence of seizures and ataxia and had continuous myoclonic jerks and was drowsy///Once *Toxoplasma* results became available started on Pyrimethamine/Sulfadimidine (x6 mo) with improvement within the first 2 wks of anti- *Toxoplasma* treatment, and then rapidly returned to normal; although some myoclonic seizures persisted until clonazepam was added///After d/c of anti- *Toxoplasma* treatment after 6 mo he remained well, and EEG showed improvement although some R sided predominance remained////
Michel 1986	Acute pulmonary toxoplasmosis//in a 21-yr-old immunocompetent Portuguese man living in Belgium, who presented w high fever, shivers, dry cough, myalgia, arthralgia and rash (duration not given); cervical LADP, b/l lung crackles, hepatomegaly//CXR: b/l interstitial infiltrates//Hypoxia (PaO2 52 mmHg); elevated LDH and CPK//PFTS with mild restrictive patterns//BAL with excess lymphocytes///Patient responded to treatment with Erythromycin and after 10 ds of treatment with resolution of fever and hypoxia and normalization of CXR.//With rising and highly positive *Toxoplasma* IgG and IgM serology (with *Toxoplasma* IgG from 1:1000 to 1:4000 and *Toxoplasma* IgM from 1/320 to 1/12(x 3 mo)//after 3 mo of anti- *Toxoplasma* treatment there was normalization of the initially low OKT4 counts and initially high OKT8 counts (before treatment OKT4: 357 & OKT8: 2589 58%; after treatment OKT4: 765 & OKT8 1135 cells/mL 43%)

Abbreviations: BNP: brain natriuretic peptide, b/l: bilateral, CT: computed tomography, DM: diabetes mellitus, ds: days, EF: ejection fraction, f/up: follow-up, HA: headache, HD: hospital day, HIV: human immunodeficiency virus, HLH: hemophagocytic lymphohistiocytosis, HSM: hepatosplenomegaly, hs-troponin: high sensitive troponin, Hx: history, ICU: intensive care unit, IMV: invasive mechanical ventilation, L: left, LADP: lymphadenopathy, LN: lymph node, LVF: left ventricular function, mo: month, nl: normal, P/S: pyrimethamine/sulfadiazine, PTA: prior to admission, R: right, unl: upper normal limit, w: with; wks: weeks; yr: year.

Secondary Analyses

**Table A9:** Secondary Analysis: Characteristics of 46 cases (36 articles) published between 1941–1984.

	N (%)
Articles	36
Cases	46
Top countries of cases (imputed when not reported by the country of authors)	
USA	16 (28%)
Ireland	6 (13%)
France	4 (13%)
Switzerland	3 (7%)
N of cases per article	
22	1/36 (3%)
11	1/36 (3%)
6	2/36 (6%)
3	1/36 (3%)
2	8/36 (22%)
1	23/36 (64%)
Age	
Adult	28 (61%)
Pediatric	13 (28%)
Not reported	5 (11%)
Manifestations	
Pulmonary	8/46 (17%)
CNS	12/46 (26%)
Cardiac	16/46 (35%)
Disseminated	6/46 (13%)
Prolonged duration (myositis, renal, other) > 3 months	4/46 (9%)

**Table A10:** REFERENCES of articles published between 1941–1984.

1. Adams EM, Hafez GR, Carnes M, Wiesner JK, Graziano FM. The development of polymyositis in a patient with toxoplasmosis: clinical and pathologic findings and review of literature. Clin Exp Rheumatol. 1984 Jul-Sep;2(3):205-8. PMID: 6529871.
2. Amato Neto V. Caso fatal de pneumonia intersticial toxoplasmótica [A fatal case of interstitial pneumonia due to toxoplasmosis]. Rev Inst Med Trop Sao Paulo. 1969 Sep-Oct;11(5):377-81. Spanish. PMID: 5356133.
3. Armstrong C, Macmurray FG. Toxoplasmosis found by recovery of toxoplasma gondii from excised maxillary gland. J Am Med Assoc. 1953 Mar 28;151(13):1103-4. doi: 10.1001/jama.1953.02940130049009a. PMID: 13034439.
4. Arribada A, Escobar E. Cardiomyopathies produced by Toxoplasma gondii. Am Heart J. 1968 Sep;76(3):329-39. doi: 10.1016/0002-8703(68)90227-5. PMID: 4951330.
5. Bach MC, Armstrong RM. Acute toxoplasmic encephalitis in a normal adult. Arch Neurol. 1983 Sep;40(9):596-7. doi: 10.1001/archneur.1983.04050080096025. PMID: 6604513.
6. Collart F, Glupzinski Y, Cogan E, Schmerber J: Acute pericarditis of toxoplasmic origin. J Infect 1983; 7: 82-83
7. Cunningham T: Pancreatitis in acute toxoplasmosis. Am J Clin Pathol 1982; 78: 403-405
8. Doffoel M, Coumaros D, Levy P, Jacques C, Saada K, Laidoudi A, Bockel R, Himy-Dahan R, Kien T. Neurotoxoplasmose. A propos d’un cas de méningo-encéphalite acquise [Neurotoxoplasmosis. Description of a case of an acquired meningo-encephalitis (author’s transl)]. Sem Hop. 1980 Apr 18-25;56(15-16):788-90. French. PMID: 6246619.
9. Espersen E. Toxoplasmosis; a survey and a case of acquired pulmonary toxoplasmosis. Acta Tuberc Scand. 1957;34(3-4):163-72. PMID: 13520397.
10. Guignard JP, Torrado A. Interstital nephritis and toxoplasmosis in a 10-year-old child. J Pediatr. 1974 Sep;85(3):381-2. doi: 10.1016/s0022-3476(74)80123-x. PMID: 4610420.
11. Guimares FN: Toxoplasmose humana: meningoencefalomielite toxoplasmica: ocorrencia em adulto e em recemnascido. Mem Inst Oswaldo Cruz 1943; 38: 257-320 (in Portugese)
12. Hafstrom T. Toxoplasmic encephalopathy: a form of meningo-encephalomyelitis in adult toxoplasmosis. Acta Psychiatr Scand. 1959;34(3):311-21. doi: 10.1111/j.1600-0447.1959.tb07581.x. PMID: 14398759.
13. Hakkila J, Frick HM, Halonen PI: Pericarditis and myocarditis caused by toxoplasma: Report of a case and review of the literature. Am Heart J 1958; 55: 758 - 766
14. Israel J, Baufine-Ducrocq H: Les Pericardites aigues d’origine toxoplasmique. Coeur Med Interne 1969; 8: 47-54 (in French)
15. Jones TC, Kean BH, Kimball AC: Pericarditis associated with toxoplasmosis. Ann Intern Med 1965: 62:786-790
16. Kass EH, Andrus SB, Adams RD, Turner FC, Feldman HA. Toxoplasmosis in the human adult. AMA Arch Intern Med. 1952 May;89(5):759-82. doi: 10.1001/archinte.1952.00240050073006. PMID: 14914215.
17. Leak D, Meghji M: Toxoplasmic infection in cardiac disease. Am J Cardiol 1979; 43: 841-849
18. Ludlam GB, Beattie CP, Pulmonary Toxoplasmosis? Lancet. 1963 Nov 30;2(7318):1136-8. doi: 10.1016/s0140-6736(63)90789-x. PMID: 14072910.
19. Mary AS, Hamilton M. Ventricular tachycardia in a patient with toxoplasmosis. Br Heart J. 1973 Mar;35(3):349-52. doi: 10.1136/hrt.35.3.349. PMID: 4692665; PMCID: PMC458615.
20. Mirada A, Rubies-Prat J, Cerda E, Foz M: Pericarditis associated to acquired toxoplasmosis in a child. Cardiology 1976; 61: 303-306
21. Mishra DN, Gupta RC, Atal PR, Shukla SP. Toxoplasmosis in an adult presenting as paraparesis (a case report). J Assoc Physicians India. 1975 Nov;23(11):779-81. PMID: 1223077.
22. Nagaratnam N, Stephen SJ: Chronic constrictive pericarditis associated with toxoplasmosis. Br Heart J 1973; 35: 868-870
23. Niutta R, Fatigante R. Miliare polmonare da toxoplasma gondii in adulto [Pulmonary miliaria caused by Toxoplasma gondii in an adult]. Minerva Med. 1978 Sep 1;69(40):2717-22. Italian. PMID: 714266.
24. Alam M, Duvernoy WFC, Quinn EL, Fisher EJ. Congestive cardiomyopathy as an end stage of Toxoplasma myocarditis. Henry Ford Hospital Medical Journal 1975: Vol. 23: No. 3, 141-144. Available at: https://scholarlycommons.henryford.com/hfhmedjournal/vol23/iss3/6.
25. Pinkerton, H., and Henderson, R.G. Adult toxoplasmosis; previously unrecognized disease entity simulating typhug-spotted fever group F. Am. M. Ass., 1941, 116, 807-814
26. Prado S, Pacheco V, Noemi I, Eimbcke F, Zilleruelo R, Vicuna D, Vera S, Saieh M, Jaeger H, Gomez O, Arretz C: Toxoplasma pericarditis and myocarditis. Rev Chil Pediatr 1978: 49: 179-185 (in Spanish with Eng abstr)
27. Sabin AB: Toxoplasmic encephalitis in children. JAMA 116:801-807, 1941.
28. Sacks JJ, Delgado DG, Lobel HO, Parker RL. Toxoplasmosis infection associated with eating undercooked venison. Am J Epidemiol. 1983 Dec;118(6):832-8. doi: 10.1093/oxfordjournals.aje.a113701. PMID: 6650484.
29. Sante LR. Roentgen manifestations of adult toxoplasmosis. AmericanJournal of Roentgenology and Radium Therapy 1942;47:825-9.
30. Sexton RCJr, Eyles DE, Dillman RE. Adult toxoplasmosis. Am J Med. 1953 Mar;14(3):366-77. doi: 10.1016/0002-9343(53)90047-3. PMID: 13030493.
31. Stagno S, Dykes AC, Amos CS, Head RA, Juranek DD, Walls K. An outbreak of toxoplasmosis linked to cats. Pediatrics. 1980 Apr;65(4):706-12. PMID: 7189277.
32. Townsend JJ, Wolinsky JS, Baringer JR, Johnson PC. Acquired toxoplasmosis. A neglected cause of treatable nervous system disease. Arch Neurol. 1975 May;32(5):335-43. doi: 10.1001/archneur.1975.00490470079012. PMID: 1094993.
33. Vischer TL, Bernheim C, Engelbrecht E. Two cases of hepatitis due to Toxoplasma gondii. Lancet. 1967 Oct 28;2(7522):919-21. doi: 10.1016/s0140-6736(67)90235-8. PMID: 4167857.
34. Walter J, Schwartz J, Bloch R, Schaub S: Existe-t-il une pericardite toxoplasmique? Presse Med 1964; 72: 3047-3048 (in French)
35. Wilkinson EE. The radiologic aspects of toxoplasmosis. J Am Med Womens Assoc (1972). 1973 Feb;28(2):76-80. PMID: 4347594.
